# Supplementary figures and images for: Human dermal fibroblast subpopulations and epithelial mesenchymal transition signals in hidradenitis suppurativa tunnels are normalized by spleen tyrosine kinase antagonism in vivo
Source: PLoS One. 2023 Nov 3;18(11):e0282763. doi: 10.1371/journal.pone.0282763 (PMC10624284; doi:10.1371/journal.pone.0282763)

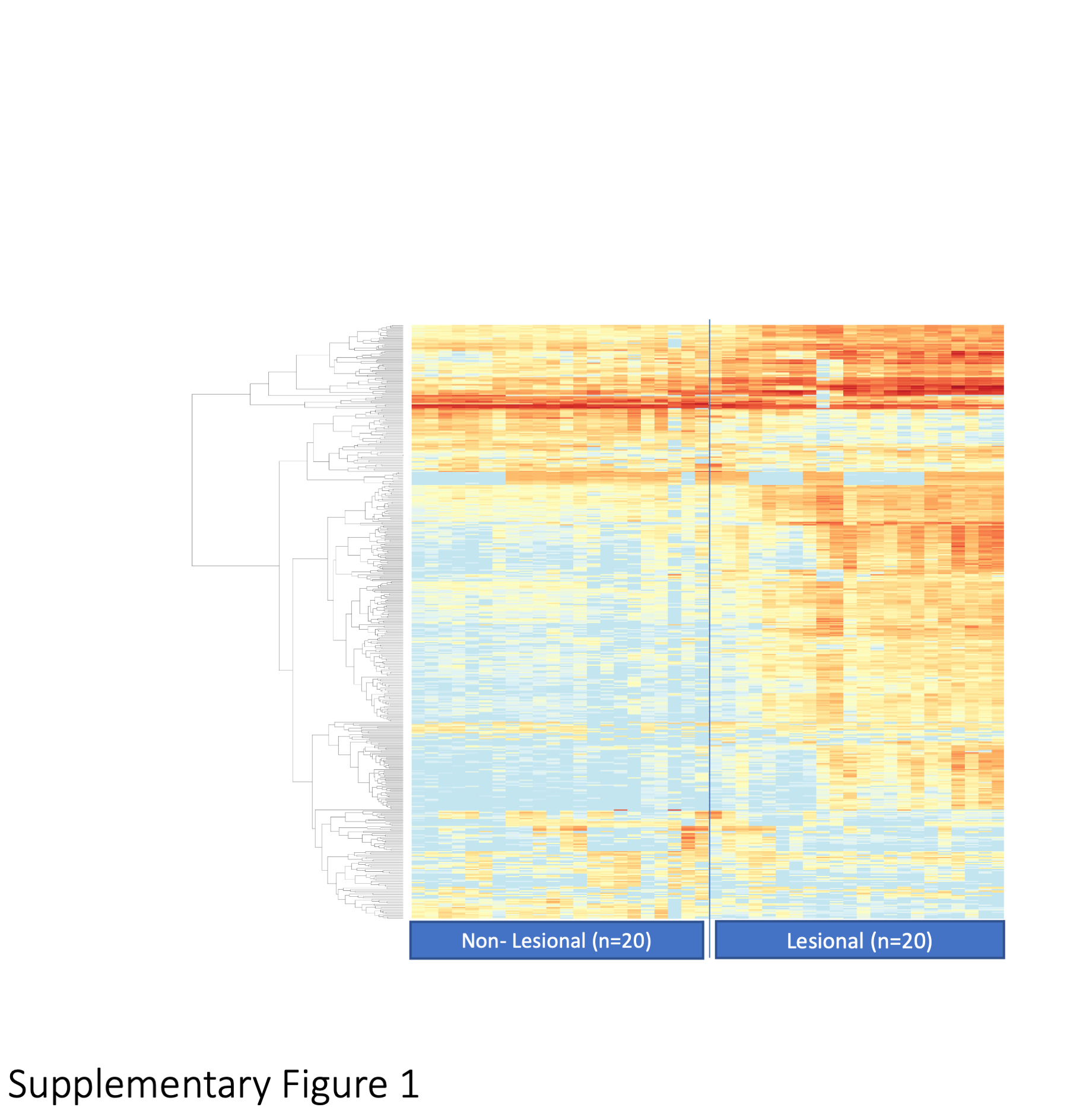

Supplement: S1 Fig — (TIFF) [file pone.0282763.s001.tiff]

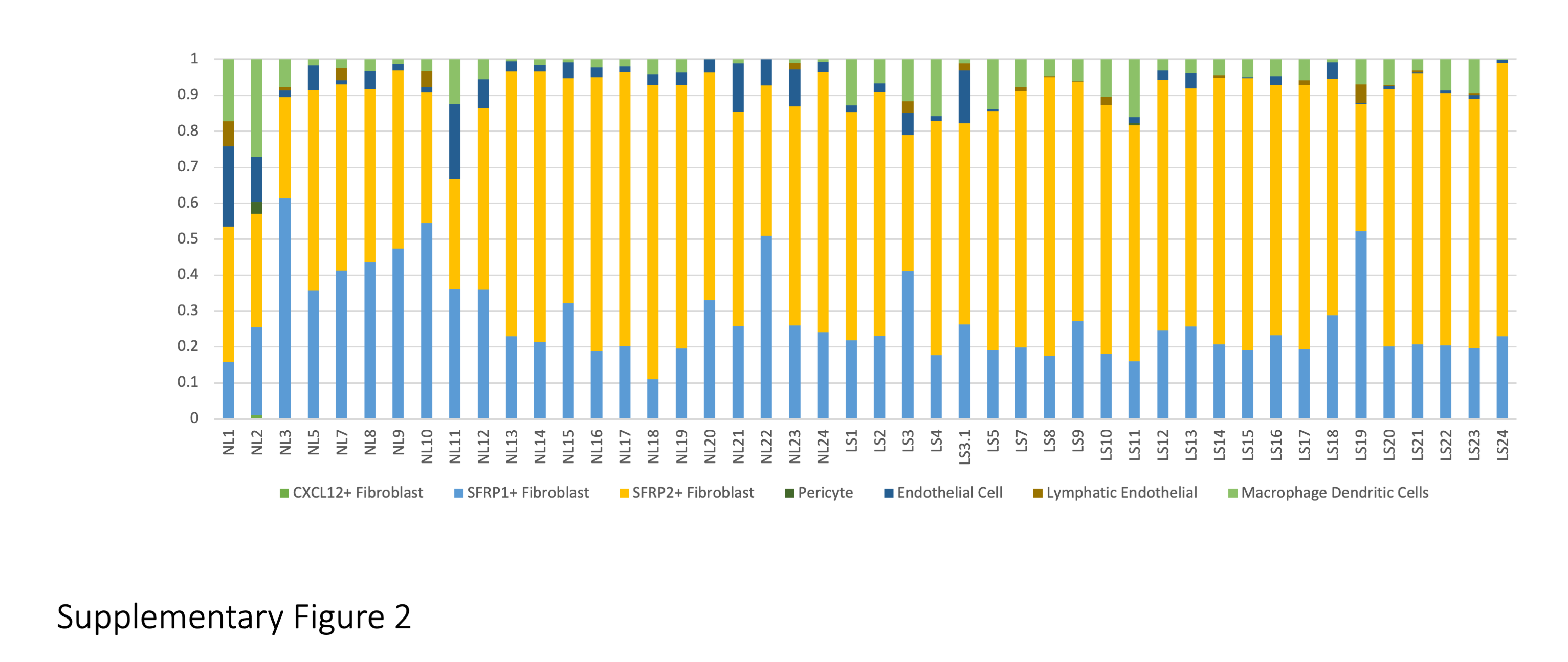

Supplement: S2 Fig — (TIFF) [file pone.0282763.s002.tiff]
