# Supplementary material for: Human dermal fibroblast subpopulations and epithelial mesenchymal transition signals in hidradenitis suppurativa tunnels are normalized by spleen tyrosine kinase antagonism in vivo
Source: PLoS One. 2023 Nov 3;18(11):e0282763. doi: 10.1371/journal.pone.0282763 (PMC10624284; doi:10.1371/journal.pone.0282763)
Supplement: S1 Table — (DOCX) [file pone.0282763.s003.docx]

SUPPLEMENTARY TABLE 1: PATIENT CHARACTERISTICS

Supplementary Table 1: Patient Characteristics for RNAseq and Nanostring Samples

RNAseq

| Patient | Age | Gender | Hurley Stage |
| --- | --- | --- | --- |
| 1 | 35 | F | 3 |
| 2 | 42 | F | 3 |
| 3 | 23 | M | 3 |
| 4 | 26 | M | 3 |
| 5 | 27 | F | 2 |
| 6 | 20 | F | 2 |
| 7 | 42 | F | 3 |
| 8 | 48 | F | 2 |
| 9 | 49 | F | 2 |
| 10 | 52 | M | 2 |
| 11 | 34 | M | 2 |
| 12 | 32 | F | 2 |
| 13 | 39 | M | 3 |
| 14 | 38 | F | 2 |
| 15 | 22 | F | 3 |
| 16 | 21 | M | 2 |
| 17 | 27 | M | 2 |
| 18 | 27 | F | 2 |
| 19 | 46 | M | 2 |
| 20 | 41 | M | 2 |

Nanostring

| Patient | Age | Gender | Hurley Stage |
| --- | --- | --- | --- |
| 1 | 35 | F | 3 |
| 2 | 64 | F | 3 |
| 3 | 58 | M | 3 |
| 4 | 30 | F | 2 |
| 5 | 42 | M | 3 |
| 6 | 23 | M | 2 |
| 7 | 28 | F | 2 |
| 8 | 35 | M | 2 |
| 9 | 27 | F | 3 |
| 10 | 48 | M | 3 |
