# Supplementary material for: Human dermal fibroblast subpopulations and epithelial mesenchymal transition signals in hidradenitis suppurativa tunnels are normalized by spleen tyrosine kinase antagonism in vivo
Source: PLoS One. 2023 Nov 3;18(11):e0282763. doi: 10.1371/journal.pone.0282763 (PMC10624284; doi:10.1371/journal.pone.0282763)
Supplement: S3 Table — (DOCX) [file pone.0282763.s005.docx]

| **Primary Antibody** |  |  |  |
| --- | --- | --- | --- |
| **Protein Target** | **Antibody** | **Species** | **Dilution** |
| E-Cadherin | Abcam 40772 | Rabbit | 1:500 |
| SNAIL/SLUG | Abcam 85936 | Rabbit | 1ug/mL |
| N-Cadherin | Abcam 76011 | Rabbit | 1:50 |
| ZEB1 | Abcam 203829 | Rabbit | 1:100 |
| Twist1 | Thermofisher PA549688 | Mouse | 1:100 |
| SFRP1 | Thermofisher PA595634 | Rabbit | 1:500 |
| SFRP2 | Thermofisher MA526867 | Mouse | 1:150 |
| CXCL12 | Thermofisher 589116 | Rabbit | 1:200 |
| **Secondary Antibodies** |  |  |  |
| **Protein Target** | **Antibody** | **Species** | **Dilution** |
| Goat Anti-mouse | Abcam 6789 | Goat | 1:2000 |
| Goat Anti-rabbit | Abcam 6721 | Goat | 1:200 |

Supplementary Table: Primary and Secondary Antibodies used in Immunohistochemical Staining
