# Supplementary material for: Human dermal fibroblast subpopulations and epithelial mesenchymal transition signals in hidradenitis suppurativa tunnels are normalized by spleen tyrosine kinase antagonism in vivo
Source: PLoS One. 2023 Nov 3;18(11):e0282763. doi: 10.1371/journal.pone.0282763 (PMC10624284; doi:10.1371/journal.pone.0282763)
Supplement: S1 File — (DOCX) [file pone.0282763.s007.docx]

| **Gene** | **Accession** | **Synonyms** | **Target** | **Full Name** |
| --- | --- | --- | --- | --- |
| ABCA1 | NM_005502.3 | ABC1,HDLDT1;ATP-binding cassette, sub-family A (ABC1), member 1 | GGGAGCCTTTGTGGAACTCTTTCATGAGATTGATGACCGGCTCTCAGACCTGGGCATTTCTAGTTATGGCATCTCAGAGACGACCCTGGAAGAAATATTC | ATP binding cassette subfamily A member 1 |
| ABCB11 | NM_003742.2 | BSEP,PFIC2;progressive familial intrahepatic cholestasis 2,bile salt export pump,ATP-binding cassette, sub-family B (MDR/TAP), member 11 | TTCTGAAATTCAGTGCTCCAGAATGGCCCTACATGCTGGTAGGGTCTGTGGGTGCAGCTGTGAACGGGACAGTCACACCCTTGTATGCCTTTTTATTCAG | ATP binding cassette subfamily B member 11 |
| ACAA2 | NM_006111.2 | acetyl-Coenzyme A acyltransferase 2 | AGCTTACGGAGGCCTTCTGAAAGACTTCACTGCTACTGACTTGTCTGAATTTGCTGCCAAGGCTGCCTTGTCTGCTGGCAAAGTCTCACCTGAAACAGTT | acetyl-CoA acyltransferase 2 |
| ACACA | NM_198834.1 | ACAC,ACC;acetyl-Coenzyme A carboxylase alpha | GACATGAACACTGTACTGAACTACATCTTCTCTCACGCTCAAGTCACCAAGAAGAATCTTCTGGTCACAATGCTTATTGATCAGTTGTGTGGCCGGGACC | acetyl-CoA carboxylase alpha |
| ACACB | NM_001093.3 | acetyl-Coenzyme A carboxylase beta | CAGCGGGATCCGCGGCTATATGAAAACAGTGGTGTTGGATCTCCTGAGAAGATACTTGCGTGTTGAGCACCATTTTCAGCAAGCCCACTACGACAAGTGT | acetyl-CoA carboxylase beta |
| ACOX2 | NM_003500.3 | acyl-Coenzyme A oxidase 2, branched chain,acyl-CoA oxidase 2, branched chain | ACTACCAGACACAACAGCAGAAACTCTTTCCTCAGCTGGCCATCAGTTATGCCTTCCATTTCCTGGCAGTCAGCCTCTTGGAGTTCTTCCAGCACTCCTA | acyl-CoA oxidase 2 |
| ACSL4 | NM_004458.2 | FACL4,MRX63,MRX68;fatty-acid-Coenzyme A ligase, long-chain 4,mental retardation, X-linked 63,mental retardation, X-linked 68 | TTCGACTCACTAGCTGTAATAGACATCCCTGGAGCAGATACTCTGGATAAATTATTTGACCATGCTGTATCCAAGTTTGGGAAGAAGGACAGCCTTGGGA | acyl-CoA synthetase long chain family member 4 |
| ACSM3 | NM_005622.3 | SAH;SA (rat hypertension-associated) homolog,SA hypertension-associated homolog (rat) | TCTGTAAATGGAAGGTTCTGGCTAGATTTGACACCCTCAGATGTGATGTGGAATACCTCAGATACGGGCTGGGCAAAGTCTGCATGGAGTAGTGTTTTTT | acyl-CoA synthetase medium chain family member 3 |
| ACTA2 | NM_001613.1 | actin, alpha 2, smooth muscle, aorta | ATTCCTTCGTTACTACTGCTGAGCGTGAGATTGTCCGGGACATCAAGGAGAAACTGTGTTATGTAGCTCTGGACTTTGAAAATGAGATGGCCACTGCCGC | actin alpha 2, smooth muscle |
| ACTR1A | NM_005736.3 | ARP1 (actin-related protein 1, yeast) homolog A (centractin alpha),ARP1 actin-related protein 1 homolog A, centractin alpha,ARP1 actin related protein 1 homolog A | AAAAACCGGGAACGAGCTGCCGAAGTTTTCTTCGAGACCTTCAATGTGCCCGCTCTTTTCATCTCCATGCAAGCTGTACTCAGCCTTTACGCTACAGGCA | actin related protein 1A |
| ACVRL1 | NM_000020.1 | ACVRLK1,ORW2;activin A receptor type II-like 1,activin A receptor type IL | TCTGCCCGACTCACCGCGCTGCGGATCAAGAAGACACTACAAAAAATTAGCAACAGTCCAGAGAAGCCTAAAGTGATTCAATAGCCCAGGAGCACCTGAT | activin A receptor like type 1 |
| ADA2 | NM_001282229.1 | IDGFL,CECR1;cat eye syndrome chromosome region, candidate 1 | GTCTTGCCCACTCCTGTTTACCCTTCAAGTTTCAAGTTCATGTCACTGTCTCAGAGAGGTTTTCCTGTGCTCGCCCTGTTTCTCTCAGGAAGCCTTGCTC | adenosine deaminase 2 |
| ADAM17 | NM_003183.4 | TACE;tumor necrosis factor, alpha, converting enzyme | AGAACGCAGCAATAAAGTTTGTGGGAACTCGAGGGTGGATGAAGGAGAAGAGTGTGATCCTGGCATCATGTATCTGAACAACGACACCTGCTGCAACAGC | ADAM metallopeptidase domain 17 |
| ADAM9 | NM_001005845.1 | CORD9;a disintegrin and metalloproteinase domain 9 (meltrin gamma),cone rod dystrophy 9 | TGTCTTGCCACAGACCCGGTATGTGGAGCTGTTCATTGTCGTAGACAAGGAAAGGTATGACATGATGGGAAGAAATCAGACTGCTGTGAGAGAAGAGATG | ADAM metallopeptidase domain 9 |
| ADCY7 | NM_001114.4 |  | AGAACCTGCTGCTGTCAGTGCTTCCGGCCCACATCTCCATGGGCATGAAGCTGGCCATCATCGAACGGCTCAAGGAGCATGGTGACCGTCGCTGCATGCC | adenylate cyclase 7 |
| ADH1B | NM_000668.4 | ADH2 | ATAATCTTTAGTCATCGAATCCCAGTGGAGGGGACCCTTTTACTTGCCCTGAACATACACATGCTGGGCCATTGTGATTGAAGTCTTCTAACTCTGTCTC | alcohol dehydrogenase 1B (class I), beta polypeptide |
| ADH1C | NM_000669.3 | ADH3 | GATTCCCAGAACCTCTCAATAAACCCTATGCTGCTACTGACTGGACGCACGTGGAAAGGAGCTATTTTTGGAGGCTTTAAGAGTAAAGAATCTGTCCCCA | alcohol dehydrogenase 1C (class I), gamma polypeptide |
| ADH4 | NM_000670.3 |  | ATTCAGATCATTGCTACCTCCCTGTGCCATACTGATGCCACTGTTATCGATTCTAAATTTGAGGGCCTAGCTTTCCCAGTGATCGTTGGCCATGAGGCTG | alcohol dehydrogenase 4 (class II), pi polypeptide |
| ADH6 | NM_000672.3 |  | GAAAGTTGCTACAGGATCTCCCTTTCTCAATAAATTCATCTGCGGTGGAGAAAATCAGCATGAGTACTACAGGCCAAGTCATCAGATGCAAAGCAGCCAT | alcohol dehydrogenase 6 (class V) |
| ADIPOQ | NM_004797.2 | ACDC;adipocyte, C1Q and collagen domain containing | GCTTTCTTCTCTACCATGACACCAACTGATCACCACTAACTCAGAGCCTCCTCCAGGCCAAACAGCCCCAAAGTCAATTAAAGGCTTTCAGTACGGTTAG | adiponectin, C1Q and collagen domain containing |
| ADIPOR1 | NM_015999.3 |  | TTTCACACCCACTGGGCAATAAACTTTCCATTTCCATTCTCCTAGCTGGGGATGGGGCATGGTCAAACTTAGCCATCCCCTCCTCAGCAAGGCATCTACC | adiponectin receptor 1 |
| ADPGK | NR_023318.1 |  | TTCCCATTCGGAGGCAGGCTCCAGGATTGTATTAAACCCAAACAAGCCAGTAGTAGAATGGCACAGAGAGGGAATATCCTTCCACTTCACACCAGTATTG | ADP dependent glucokinase |
| AIM2 | NM_004833.1 |  | ACGTGCTGCACCAAAAGTCTCTCCTCATGTTAAGCCTGAACAGAAACAGATGGTGGCCCAGCAGGAATCTATCAGAGAAGGGTTTCAGAAGCGCTGTTTG | absent in melanoma 2 |
| AKR1B10 | NM_020299.3 | AKR1B11;aldo-keto reductase family 1, member B10 (aldose reductase) | GCTGAAGTGTGACTACCTCCACTCATGTCCCATTTTAGCCAAGCTTATTTAAGATCACAGTGAACTTAGTCCTGTTATAGACGAGAATCGAGGTGCTGTT | aldo-keto reductase family 1 member B10 |
| AKT1 | NM_001014432.1 | v-akt murine thymoma viral oncogene homolog 1 | GGACGGGCACATTAAGATCACAGACTTCGGGCTGTGCAAGGAGGGGATCAAGGACGGTGCCACCATGAAGACCTTTTGCGGCACACCTGAGTACCTGGCC | AKT serine/threonine kinase 1 |
| ALAD | NM_000031.5 | aminolevulinate, delta-, dehydratase | ACCTCCTGGTGGCCTGTGATGTCTGCCTGTGTCCCTACACCTCCCATGGTCACTGCGGGCTCCTGAGTGAAAACGGAGCATTCCGGGCTGAGGAGAGCCG | aminolevulinate dehydratase |
| ALDH3A2 | NM_001031806.1 | SLS,ALDH10;aldehyde dehydrogenase 3 family, member A2 | ATTGTTTGCAGACGCATAACCTGGGGAAAATACATGAATTGTGGCCAAACCTGCATTGCACCCGACTATATTCTCTGTGAAGCATCCCTCCAAAATCAAA | aldehyde dehydrogenase 3 family member A2 |
| ALDH7A1 | NM_001182.3 | ATQ1;aldehyde dehydrogenase 7 family, member A1 | GCTTTAGGCGTCTGCGGTTGCCCTTGGATCTGTTCTCAATCCTCAGTGTGTGTGGCAGCATGTGGTCATAGAGAGCTGGGCAAAGTTCACTTTCTCTTTG | aldehyde dehydrogenase 7 family member A1 |
| ALDH9A1 | NM_000696.3 | ALDH7,ALDH4,ALDH9;aldehyde dehydrogenase 9 family, member A1 | AACCACTTGGGGTATGTGTGGGAATAGGAGCATGGAACTACCCCTTTCAGATTGCCTCTTGGAAGTCGGCTCCAGCATTAGCCTGTGGTAATGCCATGGT | aldehyde dehydrogenase 9 family member A1 |
| ALDOB | NM_000035.3 | aldolase B, fructose-bisphosphate | CATCCAAAGAACAACTGCTGATTGAAACACCTCATTAGCTGAGTGTAGAGAAGTGCATCTTATGAAACAGTCTTAGCAGTGGTAGGTTGGGAAGGAGATA | aldolase, fructose-bisphosphate B |
| AMOTL1 | NM_001301007.1 |  | CTACGACAATGCCGACAAGCTCCACAAGTTTGAAAAAGAACTTCAGAGAATTTCGGAAGCCTATGAAAGTCTGGTCAAGTCTACCACCAAGCGAGAATCG | angiomotin like 1 |
| AMOTL2 | NM_016201.2 |  | ACTGTCAGTTTCTGAAACTTCTGACTGGCCTCCCAGTTATGCCTCCTCCTCAAGTTCCTGGCCCGTGGATGTTAAAGCTGCTCGATTCCCAGGATCTCGG | angiomotin like 2 |
| ANAPC1 | NM_022662.2 |  | ACTTACATTCATTCTATCAGAGATCCTGTCCATAACAGAGTCACCCTGGAACTGAGTAATGGCTCCATGGTTAGGATCACTATTCCTGAAATTGCCACCT | anaphase promoting complex subunit 1 |
| ANAPC10 | NM_001256710.1 |  | GCTGGATTCATGTTCCCTTAACTGACAATCATAAGAAGCCAACTCGTACATTCATGATACAGATTGCTGTTCTAGCCAATCACCAGAATGGAAGAGACAC | anaphase promoting complex subunit 10 |
| ANAPC7 | NM_016238.2 |  | ACACTGGTGACAACTCAAGAGCAATCAGTACCATCTGTTCACTAGAGAAAAAATCCTTATTGCGAGATAACGTGGACCTATTGGGAAGCTTGGCAGATCT | anaphase promoting complex subunit 7 |
| ANGPTL4 | NR_104213.1 | angiopoietin-like 4 | AGAGCCGGGTGGACCCTGAGGTCCTTCACAGCCTGCAGACACAACTCAAGGCTCAGAACAGCAGGATCCAGCAACTCTTCCACAAGGTGGCCCAGCAGCA | angiopoietin like 4 |
| AP1G1 | NM_001030007.1 | CLAPG1,ADTG;adaptor-related protein complex 1, gamma 1 subunit,adaptor related protein complex 1 gamma 1 subunit | GTCCCAGCATTTAACACGGGGACCATCACACAAGTCATTAAAGTTCTGAACCCTCAGAAGCAACAGCTGCGAATGCGGATCAAGCTTACATATAATCACA | adaptor related protein complex 1 subunit gamma 1 |
| AP1S1 | NM_001283.3 | CLAPS1,EKV3;erythrokeratodermia variabilis 3 (Kamouraska type),adaptor related protein complex 1 sigma 1 subunit | CTGGAGCTGATCCACCGATACGTGGAGCTCTTAGACAAATACTTTGGCAGTGTGTGCGAGCTGGACATCATCTTCAACTTTGAGAAGGCCTACTTCATCC | adaptor related protein complex 1 subunit sigma 1 |
| AP2S1 | NM_021575.2 | CLAPS2,HHC3;hypocalciuric hypercalcemia 3 (Oklahoma type),adaptor related protein complex 2 sigma 1 subunit | TGGCTGTGGGCAGAGGCCACCGTGTGTGTCCCGAGTAACCGTGCCGTTGTCGTGTGATGCCATAAGCGTCTGTGCGTGGAGTCCCCAATAAACCTGTGGT | adaptor related protein complex 2 subunit sigma 1 |
| APC | NM_000038.3 | adenomatosis polyposis coli,adenomatous polyposis coli,APC, WNT signaling pathway regulator | AAACAGCCACCACTTCTCCTAGAGGAGCCAAGCCATCTGTGAAATCAGAATTAAGCCCTGTTGCCAGGCAGACATCCCAAATAGGTGGGTCAAGTAAAGC | APC regulator of WNT signaling pathway |
| APCS | NM_001639.3 |  | GTCTGAGGTCTTGACTCAACGAGAGCACTTGAAAATGAAATGACTGTCTAAGAGATCTGGTCAAAGCAACTGGATACTAGATCTTACATCTGCAGCTCTT | amyloid P component, serum |
| APLP2 | NM_001243299.1 | APPL2;amyloid beta (A4) precursor-like protein 2 | GAAGCACGTGAGAGACCCTCGCAAAATGATGTGAAAGGACCAGTTTCTTGAAGTCCAGTGTTTCCACGGCTGGATACCTGTGTGTCTCCATAAAAGTCCT | amyloid beta precursor like protein 2 |
| APOA1 | NM_000039.1 | apolipoprotein A-I | CTGGCCACTGTGTACGTGGATGTGCTCAAAGACAGCGGCAGAGACTATGTGTCCCAGTTTGAAGGCTCCGCCTTGGGAAAACAGCTAAACCTAAAGCTCC | apolipoprotein A1 |
| APOA2 | NM_001643.1 | apolipoprotein A-II | GAAGCTGCTCGCAGCAACTGTGCTACTCCTCACCATCTGCAGCCTTGAAGGAGCTTTGGTTCGGAGACAGGCAAAGGAGCCATGTGTGGAGAGCCTGGTT | apolipoprotein A2 |
| APOA4 | NM_000482.3 | apolipoprotein A-IV | ACCTGTCTGTCTGTCTGTCCCAAAGAAGTTCTGGTATGAACTTGAGGACACATGTCCAGTGGGAGGTGAGACCACCTCTCAATATTCAATAAAGCTGCTG | apolipoprotein A4 |
| APOB | NM_000384.2 | apolipoprotein B (including Ag(x) antigen) | CACCAACTTCTTCCACGAGTCGGGTCTGGAGGCTCATGTTGCCCTAAAAGCTGGGAAGCTGAAGTTTATCATTCCTTCCCCAAAGAGACCAGTCAAGCTG | apolipoprotein B |
| APOC2 | NM_000483.3 | apolipoprotein C-II | TTGCTTCTCATGGATGGCACTGCTTTTCTGAGGACTCAAGGGCCAAGATGGAGGGGCTGACTCAGTCCAGCCAACATTTAATGAGCACCTACTTTATGTA | apolipoprotein C2 |
| APOC3 | NM_000040.1 | apolipoprotein C-III | CGAGCTCCTTGGGTCCTGCAATCTCCAGGGCTGCCCCTGTAGGTTGCTTAAAAGGGACAGTATTCTCAGTGCTCTCCTACCCCACCTCATGCCTGGCCCC | apolipoprotein C3 |
| APOH | NM_000042.2 | B2G1;apolipoprotein H (beta-2-glycoprotein I) | TGCCACAACATGCGATGTTTGGAAATGATACAATTACCTGCACGACACATGGAAATTGGACTAAATTACCAGAATGCAGGGAAGTAAAATGCCCATTCCC | apolipoprotein H |
| APP | NM_000484.3 | AD1;Alzheimer disease,amyloid beta (A4) precursor protein | TAAAGCATTTCGAGCATGTGCGCATGGTGGATCCCAAGAAAGCCGCTCAGATCCGGTCCCAGGTTATGACACACCTCCGTGTGATTTATGAGCGCATGAA | amyloid beta precursor protein |
| AQP8 | NM_001169.2 |  | TCCCAGCAGCTCAGGCAAGAGTCCGATGTTTGTGCCATCTGATCCTGATGTCTGGAGAGATAGCCATGTGTGAGCCTGAATTTGGCAATGACAAGGCCAG | aquaporin 8 |
| ARG1 | NM_000045.3 | arginase, liver | TCAATGACTGAAGTGGACAGACTAGGAATTGGCAAGGTGATGGAAGAAACACTCAGCTATCTACTAGGAAGAAAGAAAAGGCCAATTCATCTAAGTTTTG | arginase 1 |
| ARHGAP35 | NM_004491.4 | GRLF1;glucocorticoid receptor DNA binding factor 1 | ATTCAGAAGAAGATATCGAGCCATCTTACAGCCTGTTTCGAGAAGACACATCACTGCCTTCTCTGTCCAAAGACCATTCTAAGCTCTCTATGGAACTGGA | Rho GTPase activating protein 35 |
| ARHGEF2 | NM_004723.2 | rho/rac guanine nucleotide exchange factor (GEF) 2,Rho/Rac guanine nucleotide exchange factor (GEF) 2 | CTGGCTTCCTGGAGTTGATGGAATAAAGGTTGGGGTGGCCATAATGGTTTGTTGGGGGTGAGGGAAAAAACCCACAGGGACCAGAATGTTTTGTTGTTCT | Rho/Rac guanine nucleotide exchange factor 2 |
| ARHGEF6 | NM_004840.2 | MRX46;mental retardation, X-linked 46,Rac/Cdc42 guanine nucleotide exchange factor (GEF) 6 | GTGCAAACCATCCTTCAGCTGTAAATGTGCTCACTCAGCACAGTGATGAGTTGGAACAATTCATGGAAAATCAAGGTGCATCGAGCCCAGGTATCCTCAT | Rac/Cdc42 guanine nucleotide exchange factor 6 |
| ARRB1 | NM_004041.3 | ARR1;arrestin, beta 1 | CTTGCGGTGTGGACTATGAAGTCAAAGCCTTCTGCGCGGAGAATTTGGAGGAGAAGATCCACAAGCGGAATTCTGTGCGTCTGGTCATCCGGAAGGTTCA | arrestin beta 1 |
| ATF4 | NM_001675.2 | TXREB;activating transcription factor 4 (tax-responsive enhancer element B67) | TTTGAAGGAGTTCGACTTGGATGCCCTGTTGGGTATAGATGACCTGGAAACCATGCCAGATGACCTTCTGACCACGTTGGATGACACTTGTGATCTCTTT | activating transcription factor 4 |
| ATF7IP | NM_018179.3 |  | TGATTTTGCATGTACCTGTTGCAGTATCCTCCCAGCCTCAGCTTCTACAGAGCCATCCAGGGACTTTGGTGACTAATCAACCATCTGGCAATGTTGAATT | activating transcription factor 7 interacting protein |
| ATG101 | NM_021934.4 | C12orf44;chromosome 12 open reading frame 44 | CGTGGAGGTGATGAATCGGCATGAGTACTTGCCCAAGATGCCCACACAGTCGGAGGTGGATAACGTGTTTGACACAGGCTTGCGGGACGTGCAGCCCTAC | autophagy related 101 |
| ATG2B | NM_018036.5 | C14orf103;chromosome 14 open reading frame 103,ATG2 autophagy related 2 homolog B (S. cerevisiae) | CTGTGCTTCAGCCCACTTGGGGAGAGTTCCTTGATCATCATAAAGAACAGCCAGTAAGAGGGTCAACATTTCCATCCAACCTAGTTCACCCAACACCTTT | autophagy related 2B |
| ATOX1 | NM_004045.3 | ATX1 (antioxidant protein 1, yeast) homolog 1,ATX1 antioxidant protein 1 homolog (yeast) | GCTGAAGCTGTCTCTCGGGTCCTCAATAAGCTTGGAGGAGTTAAGTATGACATTGACCTGCCCAACAAGAAGGTCTGCATTGAATCTGAGCACAGCATGG | antioxidant 1 copper chaperone |
| ATP6V1E1 | NM_001039367.1 | ATP6E,ATP6V1E;ATPase, H+ transporting, lysosomal (vacuolar proton pump) 31kD,ATPase, H+ transporting, lysosomal 31kDa, V1 subunit E isoform 1,ATPase, H+ transporting, lysosomal 31kDa, V1 subunit E1 | TTTGGTGTTTGGCCCTGCAGTCCCCACTCTTGAGGCTTAAGGCGCATGTGGCACACCACTCCTTCCAGCAGTAGTCGCTTTACTGTTACCTGTTTAGGCC | ATPase H+ transporting V1 subunit E1 |
| ATP6V1F | NM_001198909.1 | ATPase, H+ transporting, lysosomal 14kDa, V1 subunit F | AGGTGAGGCGCTTCTAGGTTGCTGGGGCTCTGCTGGTTAAGGAACAGGAAGCCTGACCATCTCCCTCCACTACCTCTTCCCTGTGCTGTTACACAGTGTC | ATPase H+ transporting V1 subunit F |
| ATP6V1G2 | NM_130463.2 | ATP6G,ATP6G2;ATPase, H+ transporting, lysosomal (vacuolar proton pump),ATPase, H+ transporting, lysosomal 13kDa, V1 subunit G isoform 2,ATPase, H+ transporting, lysosomal 13kDa, V1 subunit G2 | GAAAACATCCTGACTTCAGTGTCTGGCCGATGTGGGTCCCTCTCTTGACCCTGTCACTTGCTGGCTGTGAAACCAGGACAAGCTACTTAACTTGGTAGCC | ATPase H+ transporting V1 subunit G2 |
| ATP7A | NM_000052.4 | MNK;Menkes syndrome,ATPase, Cu++ transporting, alpha polypeptide | AGCCGCAGCTACTGTGACTTCTCCGATTGTGTGAGCTTTGTTGGAGCCTGCGTACGTGGATTTATCGCTGCCACGGTCTGCGTAGCTCCAGAGGTTTAAC | ATPase copper transporting alpha |
| AXL | NM_021913.2 |  | AGCTGGAGGATTTCCTGAGTGAAGCGGTCTGCATGAAGGAATTTGACCATCCCAACGTCATGAGGCTCATCGGTGTCTGTTTCCAGGGTTCTGAACGAGA | AXL receptor tyrosine kinase |
| BANF1 | NM_001143985.1 |  | GGAGGAAAGGGGTTTTGACAAGGCCTATGTTGTCCTTGGCCAGTTTCTGGTGCTAAAGAAAGATGAAGACCTCTTCCGGGAATGGCTGAAAGACACTTGT | barrier to autointegration factor 1 |
| BAX | NM_138761.3 | BCL2-associated X protein,BCL2 associated X protein | TTTTTCCGAGTGGCAGCTGACATGTTTTCTGACGGCAACTTCAACTGGGGCCGGGTTGTCGCCCTTTTCTACTTTGCCAGCAAACTGGTGCTCAAGGCCC | BCL2 associated X, apoptosis regulator |
| BCAP31 | NM_001139457.1 |  | GCACTTCCACATGAAGCTTTTCCGTGCCCAGAGGAATCTCTACATTGCTGGCTTTTCCTTGCTGCTGTCCTTCCTGCTTAGACGCCTGGTGACTCTCATT | B cell receptor associated protein 31 |
| BCL2 | NM_000657.2 | B-cell CLL/lymphoma 2,BCL2, apoptosis regulator | AGTTCGGTGGGGTCATGTGTGTGGAGAGCGTCAACCGGGAGATGTCGCCCCTGGTGGACAACATCGCCCTGTGGATGACTGAGTACCTGAACCGGCACCT | BCL2 apoptosis regulator |
| BCL2L1 | NM_138578.1 |  | CTAAGAGCCATTTAGGGGCCACTTTTGACTAGGGATTCAGGCTGCTTGGGATAAAGATGCAAGGACCAGGACTCCCTCCTCACCTCTGGACTGGCTAGAG | BCL2 like 1 |
| BIRC3 | NM_182962.1 | API2;baculoviral IAP repeat-containing 3 | GTGAGACTCGCGCCCTCCGGCACGGAAAAGGCCAGGCGACAGGTGTCGCTTGAAAAGACTGGGCTTGTCCTTGCTGGTGCATGCGTCGTCGGCCTCTGGG | baculoviral IAP repeat containing 3 |
| BLK | NM_001715.2 | B lymphoid tyrosine kinase | AGCTTCTTGCTCCAATCAACAAGGCCGGCTCCTTTCTTATCAGAGAGAGTGAAACCAACAAAGGTGCCTTCTCCCTGTCTGTGAAGGATGTCACCACCCA | BLK proto-oncogene, Src family tyrosine kinase |
| BMP10 | NM_014482.1 |  | AGATCGGACCTCCATGCCCTCTGCCAACATCATTAGGAGTTTCAAGAATGAAGATCTGTTTTCCCAGCCGGTCAGTTTTAATGGGCTCCGAAAATACCCC | bone morphogenetic protein 10 |
| BPI | NM_001725.2 | bactericidal/permeability-increasing protein | TCTGTATCCTCCGAGCTGCAACCTTATTTCCAGACTCTGCCAGTAATGACCAAAATAGATTCTGTGGCTGGAATCAACTATGGTCTGGTGGCACCTCCAG | bactericidal permeability increasing protein |
| BRAF | NM_004333.3 | v-raf murine sarcoma viral oncogene homolog B | GTACCTGCAAGGTGTGGAGTTACAGTCCGAGACAGTCTAAAGAAAGCACTGATGATGAGAGGTCTAATCCCAGAGTGCTGTGCTGTTTACAGAATTCAGG | B-Raf proto-oncogene, serine/threonine kinase |
| BRPF3 | NM_015695.2 | bromodomain and PHD finger containing, 3 | CATTGATGGACGCCTGCATCGTATCAGCATCTATGACCCACTCAAAATCATTACTGAAGATGAGCTAACTGCCCAGGATATCACCGAATGCAATAGTAAC | bromodomain and PHD finger containing 3 |
| BST2 | NM_004335.2 |  | TTCGTATGACTATTGCAGAGTGCCCATGGAAGACGGGGATAAGCGCTGTAAGCTTCTGCTGGGGATAGGAATTCTGGTGCTCCTGATCATCGTGATTCTG | bone marrow stromal cell antigen 2 |
| C1S | NM_001734.2 | complement component 1, s subcomponent | ACTGCACTGATTGGGGAGATTGCAAGTCCCAATTATCCCAAACCATATCCAGAGAACTCAAGGTGTGAATACCAGATCCGGTTGGAGAAAGGGTTCCAAG | complement C1s |
| C3 | NM_000064.2 | complement component 3 | CATCTACCTGGACAAGGTCTCACACTCTGAGGATGACTGTCTAGCTTTCAAAGTTCACCAATACTTTAATGTAGAGCTTATCCAGCCTGGAGCAGTCAAG | complement C3 |
| C3AR1 | NM_004054.2 | complement component 3a receptor 1 | CAGTGTCTTCCTGCTTACTGCCATTAGCCTGGATCGCTGTCTTGTGGTATTCAAGCCAATCTGGTGTCAGAATCATCGCAATGTAGGGATGGCCTGCTCT | complement C3a receptor 1 |
| C4BPA | NM_000715.3 | C4BP;complement component 4-binding protein, alpha | CTCTCCCACAATGTGAAATTGTCAAGTGTAAGCCTCCTCCAGACATCAGGAATGGAAGGCACAGCGGTGAAGAAAATTTCTACGCATACGGCTTTTCTGT | complement component 4 binding protein alpha |
| C5 | NM_001735.2 | complement component 5 | ATTCGTTGAATGACGACTTGAAGCCAGCCAAAAGAGAAACTGTCTTAACTTTCATAGATCCTGAAGGATCAGAAGTTGACATGGTAGAAGAAATTGATCA | complement C5 |
| C6 | NM_000065.2 | complement component 6 | ACTCTCCTGTTTGGGCATGTCTTATTCAGTTCCAGCTCATGACGCCCTGTAGCATACCCCTAGGTACCAACTTCCACAGCAGTCTCGTAAATTCTCCTGT | complement C6 |
| C8A | NM_000562.2 | complement component 8, alpha polypeptide,complement component 8 alpha subunit | GAGCTTCGATATGACTCCACCTGTGAACGTCTCTACTATGGAGATGATGAGAAATACTTTCGGAAACCCTACAACTTTCTGAAGTACCACTTTGAAGCCC | complement C8 alpha chain |
| C8B | NM_000066.2 | complement component 8, beta polypeptide | GTTTTGAGGGCCCAGTTCTTGATCACAGGTATTATGCAGGTGGATGCTCCCCGCATTACATCCTGAACACGAGGTTTAGGAAGCCCTACAATGTGGAAAG | complement C8 beta chain |
| C9 | NM_001737.3 | complement component 9 | TTTGACAATGAGTTCTACAATGGACTCTGTAACCGGGATCGGGATGGAAACACTCTGACATACTACCGAAGACCTTGGAACGTGGCTTCTTTGATCTATG | complement C9 |
| CALM1 | NM_006888.3 | CALML2;calmodulin 1 (phosphorylase kinase, delta) | ACCTTGCCTGAAAACAAGCAGATACCGATTGCTTCATCCTATTTATGGACATGTAGGTCTAGTTGCATTTTCACTGGGGGGAGGGGGGAAGGTGAATTAT | calmodulin 1 |
| CAPN5 | NM_004055.4 | VRNI;vitreoretinopathy, neovascular inflammatory | GAACCTGACTTTCCGACTCCAGATCCTGCTGTCTGGATCAGTGTTCTGGCTGGAGGTGCTGGATTCTCTGATTTTGCCTCCTCACTCTGTGTCTGGCTTT | calpain 5 |
| CARD9 | NM_052814.3 | caspase recruitment domain family, member 9 | GAGGCCCGACGCCTCCGGTGCATGGAGGAGAAGGAGATGTTCGAGCTGCAGTGCCTGGCACTACGTAAGGACTCCAAGATGTACAAGGACCGCATCGAGG | caspase recruitment domain family member 9 |
| CASP12 | NR_034061.1 | CASP12P1;caspase 12 pseudogene 1,caspase 12 | GTAATATCAACAAGCTGGTCCACTGAGAGGAGAGCAAGCCTGTTGAAATTGTTTTTGCTTTCACAGCTTTTTCCTGTCCGGGGAGCAGGAGAAGCCATGG | caspase 12 (gene/pseudogene) |
| CASP3 | NM_004346.3 | caspase 3, apoptosis-related cysteine protease,caspase 3, apoptosis-related cysteine peptidase | CCATCGCCAAGTAAGAAAGTGAAGCAAATCAGAAACTTGTGAAGTGGAAATGTTCTAAAGGTGGTGAGGCAATAAAAATCATAGTACTCTTTGTAGCAAA | caspase 3 |
| CASP4 | NM_001225.3 | caspase 4, apoptosis-related cysteine protease,caspase 4, apoptosis-related cysteine peptidase | CACAGAAAAAAGCCACTTAAGGTGTTGGAATCCCTGGGCAAAGATTTCCTCACTGGTGTTTTGGATAACTTGGTGGAACAAAATGTACTGAACTGGAAGG | caspase 4 |
| CASP6 | NM_032992.2 | caspase 6, apoptosis-related cysteine protease,caspase 6, apoptosis-related cysteine peptidase | AAGTTGGACACCAACATAACTGAGGTGGATGCAGCCTCCGTTTACACGCTGCCTGCTGGAGCTGACTTCCTCATGTGTTACTCTGTTGCAGAAGGATATT | caspase 6 |
| CASP7 | NM_001227.3 | caspase 7, apoptosis-related cysteine protease,caspase 7, apoptosis-related cysteine peptidase | ATCAATGACACAGATGCTAATCCTCGATACAAGATCCCAGTGGAAGCTGACTTCCTCTTCGCCTATTCCACGGTTCCAGGCTATTACTCGTGGAGGAGCC | caspase 7 |
| CASP8 | NM_001228.4 | caspase 8, apoptosis-related cysteine protease,caspase 8, apoptosis-related cysteine peptidase | AGATGGACTTCAGCAGAAATCTTTATGATATTGGGGAACAACTGGACAGTGAAGATCTGGCCTCCCTCAAGTTCCTGAGCCTGGACTACATTCCGCAAAG | caspase 8 |
| CAT | NM_001752.2 |  | ATGCTTCAGGGCCGCCTTTTTGCCTATCCTGACACTCACCGCCATCGCCTGGGACCCAATTATCTTCATATACCTGTGAACTGTCCCTACCGTGCTCGAG | catalase |
| CBL | NM_005188.2 | CBL2;Cas-Br-M (murine) ecotropic retroviral transforming sequence,Cbl proto-oncogene, E3 ubiquitin protein ligase | GTTGTGGTAAGGATGCAGGGTATTTCGCAGAACCCAGGACGGGAAGTGCCTTTGGTTCTTGGGTGGAGCTGGAACTGCAGAGCTTTGCACCTAGTCCTTT | Cbl proto-oncogene |
| CCL13 | NM_005408.2 | SCYA13;small inducible cytokine subfamily A (Cys-Cys), member 13,chemokine (C-C motif) ligand 13 | CCAGAATTATATGAAACACCTGGGCCGGAAAGCTCACACCCTGAAGACTTGAACTCTGCTACCCCTACTGAAATCAAGCTGGAGTACGTGAAATGACTTT | C-C motif chemokine ligand 13 |
| CCL19 | NM_006274.2 | SCYA19;small inducible cytokine subfamily A (Cys-Cys), member 19,chemokine (C-C motif) ligand 19 | GACCTCAGCCAAGATGAAGCGCCGCAGCAGTTAACCTATGACCGTGCAGAGGGAGCCCGGAGTCCGAGTCAAGCATTGTGAATTATTACCTAACCTGGGG | C-C motif chemokine ligand 19 |
| CCL2 | NM_002982.3 | SCYA2;small inducible cytokine A2 (monocyte chemotactic protein 1, homologous to mouse Sig-je),chemokine (C-C motif) ligand 2 | CATTCCCCAAGGGCTCGCTCAGCCAGATGCAATCAATGCCCCAGTCACCTGCTGTTATAACTTCACCAATAGGAAGATCTCAGTGCAGAGGCTCGCGAGC | C-C motif chemokine ligand 2 |
| CCL4 | NM_002984.2 | LAG1,SCYA4;small inducible cytokine A4 (homologous to mouse Mip-1b),chemokine (C-C motif) ligand 4 | GAAGCTTCCTCGCAACTTTGTGGTAGATTACTATGAGACCAGCAGCCTCTGCTCCCAGCCAGCTGTGGTATTCCAAACCAAAAGAAGCAAGCAAGTCTGT | C-C motif chemokine ligand 4 |
| CCN2 | NM_001901.2 | CTGF;connective tissue growth factor | ACCACCCTGCCGGTGGAGTTCAAGTGCCCTGACGGCGAGGTCATGAAGAAGAACATGATGTTCATCAAGACCTGTGCCTGCCATTACAACTGTCCCGGAG | cellular communication network factor 2 |
| CCNA2 | NM_001237.2 | CCNA,CCN1 | CGGGACAAAGCTGGCCTGAATCATTAATACGAAAGACTGGATATACCCTGGAAAGTCTTAAGCCTTGTCTCATGGACCTTCACCAGACCTACCTCAAAGC | cyclin A2 |
| CCR1 | NM_001295.2 | SCYAR1,CMKBR1;chemokine (C-C motif) receptor 1 | CATCATTTGGGCCCTGGCCATCTTGGCTTCCATGCCAGGCTTATACTTTTCCAAGACCCAATGGGAATTCACTCACCACACCTGCAGCCTTCACTTTCCT | C-C motif chemokine receptor 1 |
| CCR2 | NM_001123041.2 | CMKBR2;chemokine (C-C motif) receptor 2 | TCTGATCTGCTTTTTCTTATTACTCTCCCATTGTGGGCTCACTCTGCTGCAAATGAGTGGGTCTTTGGGAATGCAATGTGCAAATTATTCACAGGGCTGT | C-C motif chemokine receptor 2 |
| CCR4 | NM_005508.4 | chemokine (C-C motif) receptor 4 | TCCCTTCCTGGCTTTCTGTTCAGCACTTGTTATACTGAGCGCAACCATACCTACTGCAAAACCAAGTACTCTCTCAACTCCACGACGTGGAAGGTTCTCA | C-C motif chemokine receptor 4 |
| CCR7 | NM_001838.2 | CMKBR7,EBI1;chemokine (C-C motif) receptor 7 | TTCCGAAAACCAGGCCTTATCTCCAAGACCAGAGATAGTGGGGAGACTTCTTGGCTTGGTGAGGAAAAGCGGACATCAGCTGGTCAAACAAACTCTCTGA | C-C motif chemokine receptor 7 |
| CCS | NM_005125.1 |  | GGGTCCAGAATGGCTTCGGATTCGGGGAACCAGGGGACCCTCTGCACGTTGGAGTTCGCGGTGCAGATGACCTGTCAGAGCTGTGTGGACGCGGTGCGCA | copper chaperone for superoxide dismutase |
| CD14 | NM_000591.2 | CD14 antigen | GCCCAAGCACACTCGCCTGCCTTTTCCTGCGAACAGGTTCGCGCCTTCCCGGCCCTTACCAGCCTAGACCTGTCTGACAATCCTGGACTGGGCGAACGCG | CD14 molecule |
| CD163 | NM_004244.4 | CD163 antigen | CATCTGTGATTCGGACTTCTCTCTGGAAGCTGCCAGCGTTCTATGCAGGGAATTACAGTGTGGCACAGTTGTCTCTATCCTGGGGGGAGCTCACTTTGGA | CD163 molecule |
| CD180 | NM_005582.2 | LY64;lymphocyte antigen 64 (mouse) homolog, radioprotective, 105kD,CD180 antigen | TTCACCCAACTCCAAGAATTGGATCTGACAGCAACTCACTTGAAAGGGTTACCCTCTGGGATGAAGGGTCTGAACTTGCTCAAGAAATTAGTTCTCAGTG | CD180 molecule |
| CD19 | XM_011545981.1 | CD19 antigen | CACCCCAAGGGGCCTAAGTCATTGCTGAGCCTAGAGCTGAAGGACGATCGCCCGGCCAGAGATATGTGGGTAATGGAGACGGGTCTGTTGTTGCCCCGGG | CD19 molecule |
| CD209 | NM_001144899.1 | CD209 antigen | TAGAGCTTGTTTTTCTGGCCCATCCTTGGAGCTTTATGAGTGAGCTGGTGTGGGATGCCTTTGGGGGTGGACTTGTGTTCCAAGAATCCACTCTCTCTTC | CD209 molecule |
| CD244 | NM_001166663.1 | natural killer cell receptor 2B4,CD244 natural killer cell receptor 2B4,CD244 molecule, natural killer cell receptor 2B4 | CTCCAGGCGCTGGGGCTTTCTCAGTGGCCTTGTCAGCTCACAGCAGGCGTTAACAGCCTCTAATTGAGGAAACTGTGGCTGGACAGGTTGCAAGGCAGTT | CD244 molecule |
| CD34 | NM_001025109.1 | CD34 antigen | AGGTAAACTCCTGTCCTTTACACATTCGGCTCCCTGGAGCCAGACTCTGGTCTTCTTTGGGTAAACGTGTGACGGGGGAAAGCCAAGGTCTGGAGAAGCT | CD34 molecule |
| CD36 | NM_000072.3 | CD36 antigen (collagen type I receptor, thrombospondin receptor),CD36 molecule (thrombospondin receptor) | AGCCAAGGAAAATGTAACCCAGGACGCTGAGGACAACACAGTCTCTTTCCTGCAGCCCAATGGTGCCATCTTCGAACCTTCACTATCAGTTGGAACAGAG | CD36 molecule |
| CD3D | NM_000732.4 | T3D;CD3d antigen, delta polypeptide (TiT3 complex),CD3d molecule, delta (CD3-TCR complex) | TATCTACTGGATGAGTTCCGCTGGGAGATGGAACATAGCACGTTTCTCTCTGGCCTGGTACTGGCTACCCTTCTCTCGCAAGTGAGCCCCTTCAAGATAC | CD3d molecule |
| CD3E | NM_000733.2 | CD3e antigen, epsilon polypeptide (TiT3 complex),CD3e molecule, epsilon (CD3-TCR complex) | AAGTAACAGTCCCATGAAACAAAGATGCAGTCGGGCACTCACTGGAGAGTTCTGGGCCTCTGCCTCTTATCAGTTGGCGTTTGGGGGCAAGATGGTAATG | CD3e molecule |
| CD3G | NM_000073.2 | CD3g antigen, gamma polypeptide (TiT3 complex),CD3g molecule, gamma (CD3-TCR complex) | GAACTAAATGCAGCCACCATATCTGGCTTTCTCTTTGCTGAAATCGTCAGCATTTTCGTCCTTGCTGTTGGGGTCTACTTCATTGCTGGACAGGATGGAG | CD3g molecule |
| CD4 | NM_000616.4 | CD4 antigen (p55),T-cell surface glycoprotein CD4 | TGGCAGGCGGAGAGGGCTTCCTCCTCCAAGTCTTGGATCACCTTTGACCTGAAGAACAAGGAAGTGTCTGTAAAACGGGTTACCCAGGACCCTAAGCTCC | CD4 molecule |
| CD44 | NM_001001392.1 | MIC4,MDU2,MDU3;CD44 antigen (homing function and Indian blood group system) | ACACCATGGACAAGTTTTGGTGGCACGCAGCCTGGGGACTCTGCCTCGTGCCGCTGAGCCTGGCGCAGATCGATTTGAATATAACCTGCCGCTTTGCAGG | CD44 molecule (Indian blood group) |
| CD6 | NM_001254751.1 | CD6 antigen | AACCCTGGACACTGCATTACAGACCCGCCATCCCTGGGCCCTCAGTATCACCCGAGGAGCAACAGTGAGTCGAGCACCTCTTCAGGGGAGGATTACTGCA | CD6 molecule |
| CD68 | NM_001251.2 | CD68 antigen | ACCGGTCCATCTTGCTGCCTCTCATCATCGGCCTGATCCTTCTTGGCCTCCTCGCCCTGGTGCTTATTGCTTTCTGCATCATCCGGAGACGCCCATCCGC | CD68 molecule |
| CD84 | NM_001184879.1 | CD84 antigen (leukocyte antigen),CD84 molecule | TCTGCTAGAACAGTGCCGTGCTTTTCCACAGAAGGTTAGACCCTGAAAGAGATGGCTCAGCACCACCTATGGATCTTGCTCCTTTGCCTGCAAACCTGGC | CD84 molecule |
| CD86 | NM_175862.3 | CD28LG2;CD86 antigen (CD28 antigen ligand 2, B7-2 antigen) | CCAGCTCTGCTCCGTATGCCAAGAGGAGACTTTAATTCTCTTACTGCTTCTTTTCACTTCAGAGCACACTTATGGGCCAAGCCCAGCTTAATGGCTCATG | CD86 molecule |
| CD8A | NM_001768.5 | CD8;CD8 antigen, alpha polypeptide (p32),T-cell surface glycoprotein CD8 alpha chain | GCTCAGGGCTCTTTCCTCCACACCATTCAGGTCTTTCTTTCCGAGGCCCCTGTCTCAGGGTGAGGTGCTTGAGTCTCCAACGGCAAGGGAACAAGTACTT | CD8a molecule |
| CD8B | NM_172099.2 | CD8B1;CD8 antigen, beta polypeptide 1 (p37) | TCAGCTGAGTGTGGTTGATTTCCTTCCCACCACTGCCCAGCCCACCAAGAAGTCCACCCTCAAGAAGAGAGTGTGCCGGTTACCCAGGCCAGAGACCCAG | CD8b molecule |
| CDH2 | NM_001792.3 | NCAD;cadherin 2, type 1, N-cadherin (neuronal) | GGTCATCCCTCCAATCAACTTGCCAGAAAACTCCAGGGGACCTTTTCCTCAAGAGCTTGTCAGGATCAGGTCTGATAGAGATAAAAACCTTTCACTGCGG | cadherin 2 |
| CDH5 | NM_001795.3 | cadherin 5, type 2, VE-cadherin (vascular epithelium),cadherin 5, type 2 (vascular endothelium) | TCTCCCCTTCTCTGCCTCACCTGGTCGCCAATCCATGCTCTCTTTCTTTTCTCTGTCTACTCCTTATCCCTTGGTTTAGAGGAACCCAAGATGTGGCCTT | cadherin 5 |
| CDK4 | NM_000075.2 |  | ACTTTTAACCCACACAAGCGAATCTCTGCCTTTCGAGCTCTGCAGCACTCTTATCTACATAAGGATGAAGGTAATCCGGAGTGAGCAATGGAGTGGCTGC | cyclin dependent kinase 4 |
| CDKN1A | NM_000389.2 | CDKN1;cyclin-dependent kinase inhibitor 1A (p21, Cip1) | CATGTGTCCTGGTTCCCGTTTCTCCACCTAGACTGTAAACCTCTCGAGGGCAGGGACCACACCCTGTACTGTTCTGTGTCTTTCACAGCTCCTCCCACAA | cyclin dependent kinase inhibitor 1A |
| CDKN2C | NM_001262.2 | cyclin-dependent kinase inhibitor 2C (p18, inhibits CDK4) | ATAATGTAAACGTCAATGCACAAAATGGATTTGGAAGGACTGCGCTGCAGGTTATGAAACTTGGAAATCCCGAGATTGCCAGGAGACTGCTACTTAGAGG | cyclin dependent kinase inhibitor 2C |
| CDON | NM_016952.4 | Cdon homolog (mouse) | AAAGTCCCAGCGCACAGAATATGAAAACATACCCTGTGTTAGCCAATCAAGTTACAGCAATGCTGTCACAAAGGGAGTTTTAGAAAACTGGGGAGTCATT | cell adhesion associated, oncogene regulated |
| CEACAM3 | NM_001815.3 | CGM1;carcinoembryonic antigen-related cell adhesion molecule 3 | GCCTTCCTGTGGGGGCCGTCGCCGGCATCGTGACCGGGGTCCTGGTCGGAGTGGCGCTGGTGGCCGCGCTGGTGTGTTTCCTGCTCCTTGCCAAAACTGG | carcinoembryonic antigen related cell adhesion molecule 3 |
| CEBPA | NM_004364.2 | CEBP;CCAAT/enhancer binding protein (C/EBP), alpha,CCAAT/enhancer binding protein alpha | GAGCTGGGAGCCCGGCAACTCTAGTATTTAGGATAACCTTGTGCCTTGGAAATGCAAACTCACCGCTCCAATGCCTACTGAGTAGGGGGAGCAAATCGTG | CCAAT enhancer binding protein alpha |
| CETP | NM_000078.2 | cholesteryl ester transfer protein, plasma | AGAGATCAACGTCATCTCTAACATCATGGCCGATTTTGTCCAGACAAGGGCTGCCAGCATCCTTTCAGATGGAGACATTGGGGTGGACATTTCCCTGACA | cholesteryl ester transfer protein |
| CFH | NM_001014975.2 | HF,HF1,HF2;H factor 1 (complement) | GGAAAAATTGTCAGTAGTGCAATGGAACCAGATCGGGAATACCATTTTGGACAAGCAGTACGGTTTGTATGTAACTCAGGCTACAAGATTGAAGGAGATG | complement factor H |
| CFHR1 | NM_002113.2 | HFL1,CFHL1,CFHR1P,HFL2,CFHL1P;H factor (complement)-like 1,complement factor H-related 1 pseudogene,H factor (complement)-like 2,complement factor H-related 1 | AACAATGAGAACAACATTTCATGTGTAGAACGGGGCTGGTCCACCCCTCCCAAATGCAGGTCCACTGACACTTCCTGTGTGAATCCGCCCACAGTACAAA | complement factor H related 1 |
| CFHR4 | NM_001201550.2 | CFHL4;complement factor H-related 4 | TGTGACTCCTTCAGGAAGTTACTGGGATTACATTCATTGCACACAAGATGGTTGGTCACCAACGGTCCCATGCCTCAGAACATGCTCAAAATCAGATGTA | complement factor H related 4 |
| CFHR5 | NM_030787.3 | CFHL5;complement factor H-related 5 | GAGATACCACCTCATTCCCATTATCAGTATATCCTCCAGGGTCAACAGTGACGTACCGTTGCCAGTCCTTCTATAAACTCCAGGGCTCTGTAACTGTAAC | complement factor H related 5 |
| CFI | NM_000204.3 | IF;I factor (complement) | GTGGGGGAATTTATATTGGTGGCTGTTGGATTCTGACTGCTGCACATTGTCTCAGAGCCAGTAAAACTCATCGTTACCAAATATGGACAACAGTAGTAGA | complement factor I |
| CFLAR | NM_001127183.1 | CASP8AP1;CASP8 and FADD-like apoptosis regulator | TAGAGTGCTGATGGCAGAGATTGGTGAGGATTTGGATAAATCTGATGTGTCCTCATTAATTTTCCTCATGAAGGATTACATGGGCCGAGGCAAGATAAGC | CASP8 and FADD like apoptosis regulator |
| CFTR | NM_000492.3 | CF,ABCC7;cystic fibrosis transmembrane conductance regulator, ATP-binding cassette (sub-family C, member 7) | TTGGCACATTTCGTGTGGATCGCTCCTTTGCAAGTGGCACTCCTCATGGGGCTAATCTGGGAGTTGTTACAGGCGTCTGCCTTCTGTGGACTTGGTTTCC | cystic fibrosis transmembrane conductance regulator |
| CHI3L1 | NM_001276.2 | chitinase 3-like 1 (cartilage glycoprotein-39) | GGTCTCAAAGATTTTCCAAGATAGCCTCCAACACCCAGAGTCGCCGGACTTTCATCAAGTCAGTACCGCCATTTCTGCGCACCCATGGCTTTGATGGGCT | chitinase 3 like 1 |
| CHMP4B | NM_176812.4 | C20orf178;chromosome 20 open reading frame 178,chromatin modifying protein 4B | GTCGACTGGTTGCAGTTGAAATGACCTGAAATGTAGCCTCTGTCCTTGTAAGTCAGTTGACTTGCCGCACATCTCTTTGTGTACTTGTACGGTACTGGCA | charged multivesicular body protein 4B |
| CHUK | NM_001278.3 | TCF16;conserved helix-loop-helix ubiquitous kinase | TAGAACCCATGGAAAACTGGCTACAGTTGATGTTGAATTGGGACCCTCAGCAGAGAGGAGGACCTGTTGACCTTACTTTGAAGCAGCCAAGATGTTTTGT | component of inhibitor of nuclear factor kappa B kinase complex |
| CIITA | NM_000246.3 | MHC2TA;MHC class II transactivator,class II, major histocompatibility complex, transactivator | CTTTCCCCAAACTGGTGCGGATCCTCACGGCCTTTTCCTCCCTGCAGCATCTGGACCTGGATGCGCTGAGTGAGAACAAGATCGGGGACGAGGGTGTCTC | class II major histocompatibility complex transactivator |
| CKAP4 | NM_006825.3 | cytoskeleton-associated protein 4 | TAGATGACCTGAGGAATGATCTGGATAGGTTGTTTGTGAAAGTGGAGAAGATTCACGAAAAGGTCTAAATGAATTGCGTGTGCAGGGCGCGGATTTAAAG | cytoskeleton associated protein 4 |
| COL10A1 | NM_000493.3 | collagen, type X, alpha 1 | AACTTGGTTCATGGAGTGTTTTACGCTGAACGATACCAAATGCCCACAGGCATAAAAGGCCCACTACCCAACACCAAGACACAGTTCTTCATTCCCTACA | collagen type X alpha 1 chain |
| COL14A1 | NM_021110.1 | UND;undulin,collagen, type XIV, alpha 1 | CTTTAAGTCCACCAAGAAACCTGAGAATCTCCAATGTTGGCTCTAACAGTGCTCGATTAACCTGGGACCCAACTTCAAGACAGATCAATGGTTATCGAAT | collagen type XIV alpha 1 chain |
| COL16A1 | NM_001856.3 | collagen, type XVI, alpha 1 | CCCTGCTTGTCCTGCAGCTCGGTTGTAGGGGCCCAGCATCTTGTGTCCTCCACAGGGGCCAGTGGAGATGTGGGTTCCCCTGGCTTTGGTCTGCCTGGCC | collagen type XVI alpha 1 chain |
| COL1A1 | NM_000088.3 | collagen, type I, alpha 1 | CAGAAACATCGGATTTGGGGAACGCGTGTCAATCCCTTGTGCCGCAGGGCTGGGCGGGAGAGACTGTTCTGTTCCTTGTGTAACTGTGTTGCTGAAAGAC | collagen type I alpha 1 chain |
| COL1A2 | NM_000089.3 | OI4;osteogenesis imperfecta type IV,collagen, type I, alpha 2,collagen type I alpha 2 | CCAATGGATTTGCTGGTCCTGCTGGTGCTGCTGGTCAACCTGGTGCTAAAGGAGAAAGAGGAGCCAAAGGGCCTAAGGGTGAAAACGGTGTTGTTGGTCC | collagen type I alpha 2 chain |
| COL3A1 | NM_000090.3 | EDS4A;Ehlers-Danlos syndrome type IV, autosomal dominant,collagen, type III, alpha 1 | TTGGCACAACAGGAAGCTGTTGAAGGAGGATGTTCCCATCTTGGTCAGTCCTATGCGGATAGAGATGTCTGGAAGCCAGAACCATGCCAAATATGTGTCT | collagen type III alpha 1 chain |
| COL4A1 | NM_001845.4 | collagen, type IV, alpha 1 | TGGGCTTAAGTTTTCAAGGACCAAAAGGTGACAAGGGTGACCAAGGGGTCAGTGGGCCTCCAGGAGTACCAGGACAAGCTCAAGTTCAAGAAAAAGGAGA | collagen type IV alpha 1 chain |
| COL4A2 | NM_001846.2 | collagen type IV alpha 2 | GGCATTTCCTTGAAGGGAGAAGAAGGAATCATGGGCTTTCCTGGACTGAGGGGTTACCCTGGCTTGAGTGGTGAAAAAGGATCACCAGGACAGAAGGGAA | collagen type IV alpha 2 chain |
| COL5A1 | NM_000093.3 | collagen type V alpha 1 | AGTGGCACAGAATTGCTCTCAGCGTCCACAAGAAAAATGTCACCTTGATCCTCGACTGTAAAAAGAAGACCACCAAATTCCTCGACCGCAGCGACCACCC | collagen type V alpha 1 chain |
| COL5A3 | NM_015719.3 | collagen type V alpha 3 | TCGAATTCAGCTCTTCTCGAGCGGGATTTCTGCCCCTGTGGGATGTGGCGGCCACTGACTTTGGCCAGACGAACCAAAAGTTTGGGTTTGAACTGGGCCC | collagen type V alpha 3 chain |
| COL6A3 | NM_004369.3 | collagen, type VI, alpha 3 | AGAGCAAGCGAGACATTCTGTTCCTCTTTGACGGCTCAGCCAATCTTGTGGGCCAGTTCCCTGTTGTCCGTGACTTTCTCTACAAGATTATCGATGAGCT | collagen type VI alpha 3 chain |
| COL6A5 | NR_022012.2 | COL29A1;collagen, type XXIX, alpha 1,collagen, type VI, alpha 5,collagen type VI alpha 5 | TGTAAACGTGTTTGCCTTGAGCATCCAAGGGGCTAACAATACCCAGTTAGAAGAAATAGTGTCTTATCCTCCAGAACAGACAATTTCCACGCTGAAGTCC | collagen type VI alpha 5 chain |
| COL7A1 | NM_000094.2 | EBDCT,EBD1,EBR1;epidermolysis bullosa, dystrophic, dominant and recessive,collagen, type VII, alpha 1 | GCTCTGGGGGTGATGTGATCCGCGCCATCCGTGAGCTTAGCTACAAGGGGGGCAACACTCGCACAGGGGCTGCAATTCTCCATGTGGCTGACCATGTCTT | collagen type VII alpha 1 chain |
| COX4I2 | NM_032609.2 | COX4L2;cytochrome c oxidase subunit IV isoform 2,cytochrome c oxidase subunit IV isoform 2 (lung) | TCTTCTTCTTCATTGGATTCGCAGCTCTGGTGATTTGGTGGCAGCGGGTCTACGTATTTCCTCCAAAGCCGATCACCTTGACGGACGAGCGGAAAGCCCA | cytochrome c oxidase subunit 4I2 |
| COX6A1 | NM_004373.2 | COX6A;cytochrome c oxidase subunit VIa polypeptide 1 | ATCAGGACCAAGCCGTTTCCCTGGGGAGATGGTAACCATACTCTATTCCATAACCCTCATGTGAATCCACTTCCAACTGGCTACGAAGATGAATAAAGAG | cytochrome c oxidase subunit 6A1 |
| COX6B1 | NM_001863.4 | COX6B;cytochrome c oxidase subunit Vib,cytochrome c oxidase subunit Vib polypeptide 1 (ubiquitous),cytochrome c oxidase subunit VIb polypeptide 1 (ubiquitous) | AACCAGACTAGAAACTGCTGGCAGAACTACCTGGACTTCCACCGCTGTCAGAAGGCAATGACCGCTAAAGGAGGCGATATCTCTGTGTGCGAATGGTACC | cytochrome c oxidase subunit 6B1 |
| COX7B | NM_001866.2 | cytochrome c oxidase subunit VIIb | CAGAGCCACCAGAAACGTACACCTGATTTTCATGACAAATACGGTAATGCTGTATTAGCTAGTGGAGCCACTTTCTGTATTGTTACATGGACATATGTAG | cytochrome c oxidase subunit 7B |
| COX7C | NM_001867.2 | cytochrome c oxidase subunit VIIc | TCTGCGCCTTTCGCAGAGCTTCCAGCAGCGGTATGTTGGGCCAGAGCATCCGGAGGTTCACAACCTCTGTGGTCCGTAGGAGCCACTATGAGGAGGGCCC | cytochrome c oxidase subunit 7C |
| CPA3 | NM_001870.2 | carboxypeptidase A3 (mast cell) | ACCCACCACGTAGCTGCTAATATGATGGTGGATTTCCGAGTTAGTGAGAAGGAATCCCAAGCCATCCAGTCTGCCTTGGATCAAAATAAAATGCACTATG | carboxypeptidase A3 |
| CPB2 | NM_001872.3 | carboxypeptidase B2 (plasma, carboxypeptidase U),carboxypeptidase B2 (plasma) | TCTAGCTGCTCTTCCTAGAACCTCTAGGCAAGTTCAAGTTCTACAGAATCTTACTACAACATATGAGATTGTTCTCTGGCAGCCGGTAACAGCTGACCTT | carboxypeptidase B2 |
| CPN1 | NM_001308.2 | carboxypeptidase N, polypeptide 1, 50kD,carboxypeptidase N, polypeptide 1 | GTCTTCCAATTTGGACTGGTGTTTACAAGCGGGAAGCTAGGTGGACCTTGGATTTTGGCGGGTGAAGAGGCTAGGTTGTTTAAGGAGGTGGGGCGCGTTT | carboxypeptidase N subunit 1 |
| CPT1A | NM_001876.3 | CPT1;carnitine palmitoyltransferase 1A (liver) | CAGGCCTATTTTGGACGTGGGAAAAATAAGCAGTCTCTTGATGCTGTGGAGAAAGCAGCGTTCTTCGTGACGTTAGATGAAACTGAAGAAGGATACAGAA | carnitine palmitoyltransferase 1A |
| CR1 | XM_006711166.2 | complement component (3b/4b) receptor 1, including Knops blood group system,complement component (3b/4b) receptor 1 (Knops blood group),complement component 3b/4b receptor 1 (Knops blood group) | TTAAAGTACGAATGCCGTCCTGAGTACTACGGGAGGCCATTCTCTATCACATGTCTAGATAACCTGGTCTGGTCAAGTCCCAAAGATGTCTGTAAACGTA | complement C3b/C4b receptor 1 (Knops blood group) |
| CREB1 | NM_004379.3 |  | TTTTGAATGACTTATCTTCTGATGCACCAGGAGTGCCAAGGATTGAAGAAGAGAAGTCTGAAGAGGAGACTTCAGCACCTGCCATCACCACTGTAACGGT | cAMP responsive element binding protein 1 |
| CREB3 | NM_006368.4 | cAMP responsive element binding protein 3 (luman) | GCCTTGTCCACCATGACCACACCTACTCCCTCCCACGGGAAACTGTCTCTATGGATCTAGAGAGTGAGAGCTGTAGAAAAGAGGGGACCCAGATGACTCC | cAMP responsive element binding protein 3 |
| CREBBP | NM_004380.2 | RSTS;Rubinstein-Taybi syndrome | GTGTCAGAGACGAGAGCAAGCAAACGGAGAGGTTCGGGCCTGCTCGCTCCCGCATTGTCGAACCATGAAAAACGTTTTGAATCACATGACGCATTGTCAG | CREB binding protein |
| CRK | NM_016823.2 | v-crk avian sarcoma virus CT10 oncogene homolog | TACTCAAGAGTGGAAGGACCAATCACCTCTGATATTCTGTGGAAGGTTTTGGGGTCAAATTCTGCCCTCTGCATTCTGTGCAACTTGTATAAAAGTCAAG | CRK proto-oncogene, adaptor protein |
| CRKL | NM_005207.3 | v-crk avian sarcoma virus CT10 oncogene homolog-like | TACGCACGTCAAAATCTTTGACCCTCAAAACCCAGATGAAAACGAGTGATTGCTGTTGCCCTGTTTCCTGCTGCTTTGTTGTTCTGCCTGTCCTAGTCTC | CRK like proto-oncogene, adaptor protein |
| CRP | NM_000567.2 | C-reactive protein, pentraxin-related | GTCTTCCCAAGGATTGAGTTATGGACTTTGGGAGTGAGACATCTTCTTGCTGCTGGATTTCCAAGCTGAGAGGACGTGAACCTGGGACCACCAGTAGCCA | C-reactive protein |
| CSF1R | NM_005211.2 | FMS;McDonough feline sarcoma viral (v-fms) oncogene homolog | CATACTGGTACTGCTGTAATGAGCCAAGTGGCAGCTAAAAGTTGGGGGTGTTCTGCCCAGTCCCGTCATTCTGGGCTAGAAGGCAGGGGACCTTGGCATG | colony stimulating factor 1 receptor |
| CSF3R | NM_000760.3 | CD114;colony stimulating factor 3 receptor (granulocyte) | AGGCCCTTTCAGCTCTATGAGATCATCGTGACTCCCTTGTACCAGGACACCATGGGACCCTCCCAGCATGTCTATGCCTACTCTCAAGAAATGGCTCCCT | colony stimulating factor 3 receptor |
| CSNK1A1 | NM_001892.4 | casein kinase 1, alpha 1 | GCATATGAAAGACTCTGCCTGCTTAATTGTGCTAGAAATAACAGCATCTAAAGTGAAGACTTAAGAAAAACTTAGTGACTACTAGATTATCCTTAGGACT | casein kinase 1 alpha 1 |
| CSNK1D | NM_001893.3 | casein kinase 1, delta | GTTGTGTAAAGGCTACCCTTCCGAATTTGCCACATACCTGAATTTCTGCCGTTCCTTGCGTTTTGACGACAAGCCTGACTACTCGTACCTGCGGCAGCTT | casein kinase 1 delta |
| CSNK1E | NM_152221.2 | casein kinase 1, epsilon | GCTCAAAGCAGCCACCAAGCGCCAGAAGTATGAACGGATCAGCGAGAAGAAGATGTCAACGCCCATCGAGGTCCTCTGCAAAGGCTATCCCTCCGAATTC | casein kinase 1 epsilon |
| CSNK1G3 | NM_001031812.1 | casein kinase 1, gamma 3 | TGAATATGACTGGATTGGTAAACAGTTGCCTACTCCAGTGGGTGCAGTTCAGCAAGATCCTGCTCTGTCATCAAACAGAGAAGCACATCAACACAGAGAT | casein kinase 1 gamma 3 |
| CSNK2A1 | NM_177559.2 | casein kinase 2, alpha 1 polypeptide | CCATTCCCACCATTGTTCCTCCACCGTCCCACACTTTAGGGGGTTGGTATCTCGTGCTCTTCTCCAGAGATTACAAAAATGTAGCTTCTCAGGGGAGGCA | casein kinase 2 alpha 1 |
| CSNK2B | NM_001320.5 | casein kinase 2, beta polypeptide | GTTCTGTGGGCTCCGTGGCAATGAATTCTTCTGTGAAGTGGATGAAGACTACATCCAGGACAAATTTAATCTTACTGGACTCAATGAGCAGGTCCCTCAC | casein kinase 2 beta |
| CTBP2 | NM_001329.2 |  | GTTGCAGTGTTGAAACTACAAGAGCTAGAAAACTGAAGATGTCGTCTGCTTACGGAAGCGCTGAAAGACTAGGATGTGATTTATTAACGACCAACTTCTG | C-terminal binding protein 2 |
| CTNNB1 | NM_001098210.1 | CTNNB;catenin (cadherin-associated protein), beta 1 (88kD),catenin (cadherin-associated protein), beta 1, 88kDa,catenin (cadherin-associated protein), beta 1 | TCTTGCCCTTTGTCCCGCAAATCATGCACCTTTGCGTGAGCAGGGTGCCATTCCACGACTAGTTCAGTTGCTTGTTCGTGCACATCAGGATACCCAGCGC | catenin beta 1 |
| CTNND1 | NM_001331.2 | CTNND;catenin (cadherin-associated protein), delta 1 | AAGAAGAGCAGGAAAGATCCCGAAAGGAGGAAGAGGTGGCGAAAAATCAACTGCCCTGCTGGATTTGTCTTTCTCAGCACCTTGGCGAAGCCTTGGGTTT | catenin delta 1 |
| CTSB | NM_147780.2 |  | ACAAAAACGGCCCCGTGGAGGGAGCTTTCTCTGTGTATTCGGACTTCCTGCTCTACAAGTCAGGAGTGTACCAACACGTCACCGGAGAGATGATGGGTGG | cathepsin B |
| CTSD | NM_001909.3 | CPSD;cathepsin D (lysosomal aspartyl protease) | GAAGCCGGCGGCCCAAGCCCGACTTGCTGTTTTGTTCTGTGGTTTTCCCCTCCCTGGGTTCAGAAATGCTGCCTGCCTGTCTGTCTCTCCATCTGTTTGG | cathepsin D |
| CTSL | NM_001912.4 | CTSL1;cathepsin L1 | TGTGGGGCCCATTTCTGTTGCTATTGATGCAGGTCATGAGTCCTTCCTGTTCTATAAAGAAGGCATTTATTTTGAGCCAGACTGTAGCAGTGAAGACATG | cathepsin L |
| CTSW | NM_001335.3 | cathepsin W (lymphopain) | TGCACCGAGGGAGCAATACCTGTGGCATCACCAAGTTCCCGCTCACTGCCCGTGTGCAGAAACCGGATATGAAGCCCCGAGTCTCCTGCCCTCCCTGAAC | cathepsin W |
| CUL1 | NM_003592.2 |  | CTGGTAATGTCTGCATTCAACAATGACGCTGGCTTTGTGGCTGCTCTTGATAAGGCTTGTGGTCGCTTCATAAACAACAACGCGGTTACCAAGATGGCCC | cullin 1 |
| CUL3 | NM_003590.2 |  | CCACACCAAAGTGCAACATCCCACCAGCACCAAGACATGCTTTTGAGATATTCAGAAGGTTCTACTTAGCCAAACACAGTGGTCGACAGCTCACACTCCA | cullin 3 |
| CXCL10 | NM_001565.2 | INP10,SCYB10;small inducible cytokine subfamily B (Cys-X-Cys), member 10,chemokine (C-X-C motif) ligand 10 | GCCATAATTGTTCTTAGTTTGCAGTTACACTAAAAGGTGACCAATGATGGTCACCAAATCAGCTGCTACTACTCCTGTAGGAAGGTTAATGTTCATCATC | C-X-C motif chemokine ligand 10 |
| CXCL11 | NM_005409.4 | SCYB9B,SCYB11;small inducible cytokine subfamily B (Cys-X-Cys), member 11,chemokine (C-X-C motif) ligand 11 | TTCAAAAGAGGACGCTGTCTTTGCATAGGCCCTGGGGTAAAAGCAGTGAAAGTGGCAGATATTGAGAAAGCCTCCATAATGTACCCAAGTAACAACTGTG | C-X-C motif chemokine ligand 11 |
| CXCL12 | NM_199168.3 | SDF1A,SDF1B,SDF1;stromal cell-derived factor 1,chemokine (C-X-C motif) ligand 12 | CCGCCCGCCCGCCCGCCCGCGCCATGAACGCCAAGGTCGTGGTCGTGCTGGTCCTCGTGCTGACCGCGCTCTGCCTCAGCGACGGGAAGCCCGTCAGCCT | C-X-C motif chemokine ligand 12 |
| CXCL16 | NM_001100812.1 | chemokine (C-X-C motif) ligand 16 | CCATGGGTTCAGGAATTGATGAGCTGTCTTGATCTCAAAGAATGTGGACATGCTTACTCGGGGATTGTGGCCCACCAGAAGCATTTACTTCCTACCAGCC | C-X-C motif chemokine ligand 16 |
| CXCL2 | NM_002089.3 | GRO2;GRO2 oncogene,chemokine (C-X-C motif) ligand 2 | ATCACATGTCAGCCACTGTGATAGAGGCTGAGGAATCCAAGAAAATGGCCAGTGAGATCAATGTGACGGCAGGGAAATGTATGTGTGTCTATTTTGTAAC | C-X-C motif chemokine ligand 2 |
| CXCL8 | NM_000584.2 | IL8;interleukin 8,chemokine (C-X-C motif) ligand 8 | ACAGCAGAGCACACAAGCTTCTAGGACAAGAGCCAGGAAGAAACCACCGGAAGGAACCATCTCACTGTGTGTAAACATGACTTCCAAGCTGGCCGTGGCT | C-X-C motif chemokine ligand 8 |
| CXCR1 | NM_000634.2 | CMKAR1,IL8RA;interleukin 8 receptor, alpha,chemokine (C-X-C motif) receptor 1 | GCAGCCACCAGTCCATTGGGCAGGCAGATGTTCCTAATAAAGCTTCTGTTCCGTGCTTGTCCCTGTGGAAGTATCTTGGTTGTGACAGAGTCAAGGGTGT | C-X-C motif chemokine receptor 1 |
| CXCR3 | NM_001504.1 | GPR9;G protein-coupled receptor 9,chemokine (C-X-C motif) receptor 3 | GTGAGTGACCACCAAGTGCTAAATGACGCCGAGGTTGCCGCCCTCCTGGAGAACTTCAGCTCTTCCTATGACTATGGAGAAAACGAGAGTGACTCGTGCT | C-X-C motif chemokine receptor 3 |
| CXCR4 | NM_003467.2 | chemokine (C-X-C motif), receptor 4 (fusin),chemokine (C-X-C motif) receptor 4 | ATTGATGTGTGTCTAGGCAGGACCTGTGGCCAAGTTCTTAGTTGCTGTATGTCTCGTGGTAGGACTGTAGAAAAGGGAACTGAACATTCCAGAGCGTGTA | C-X-C motif chemokine receptor 4 |
| CXCR6 | NM_006564.1 | chemokine (C-X-C motif) receptor 6 | TTACCATGAAGACTATGGGTTCAGCAGTTTCAATGACAGCAGCCAGGAGGAGCATCAAGACTTCCTGCAGTTCAGCAAGGTCTTTCTGCCCTGCATGTAC | C-X-C motif chemokine receptor 6 |
| CYBB | NM_000397.3 | CGD;chronic granulomatous disease,cytochrome b-245, beta polypeptide | TTTGAAGCATGAAAAAAGAGGGTTGGAGGTGGAGAATTAACCTCCTGCCATGACTCTGGCTCATCTAGTCCTGCTCCTTGTGCTATAAAATAAATGCAGA | cytochrome b-245 beta chain |
| CYCS | NM_018947.4 |  | AAATCTATGCTGGTGCAGCTGAGAACTGTATCTTTGTGGGACAGTGAGAAGACTGAGAAGATGTGAATCCATGGTCTCAAAGGTGATAGGGACGATTAGA | cytochrome c, somatic |
| CYFIP1 | NM_014608.2 |  | GCAGCCATGTATAAGTCCCTAGAACTGGCGATTGGACGATTTGAAAGTGAAGATTTGACCTCCATAGTTGAGCTGGATGGCCTGTTGGAAATCAACCGCA | cytoplasmic FMR1 interacting protein 1 |
| CYP1A1 | NM_000499.3 | CYP1;cytochrome P450, subfamily I (aromatic compound-inducible), polypeptide 1,cytochrome P450, family 1, subfamily A, polypeptide 1 | GTATCAGTGACCAATGTCATCTGTGCCATTTGCTTTGGCCGGCGCTATGACCACAACCACCAAGAACTGCTTAGCCTAGTCAACCTGAATAATAATTTCG | cytochrome P450 family 1 subfamily A member 1 |
| CYP1A2 | NM_000761.3 | cytochrome P450, subfamily I (aromatic compound-inducible), polypeptide 2,cytochrome P450, family 1, subfamily A, polypeptide 2 | ACTTCGACCCTTACAATCAGGTGGTGGTGTCAGTGGCCAACGTCATTGGTGCCATGTGCTTCGGACAGCACTTCCCTGAGAGTAGCGATGAGATGCTCAG | cytochrome P450 family 1 subfamily A member 2 |
| CYP27A1 | NM_000784.3 | CYP27;cytochrome P450, subfamily XXVIIA (steroid 27-hydroxylase, cerebrotendinous xanthomatosis), polypeptide 1,cytochrome P450, family 27, subfamily A, polypeptide 1 | TTTGCTACATCCTGTTCGAGAAACGCATTGGCTGCCTGCAGCGATCCATCCCCGAGGACACCGTGACCTTCGTCAGATCCATCGGGTTAATGTTCCAGAA | cytochrome P450 family 27 subfamily A member 1 |
| CYP2C19 | NM_000769.1 | CYP2C;cytochrome P450, subfamily IIC (mephenytoin 4-hydroxylase), polypeptide 19,cytochrome P450, family 2, subfamily C, polypeptide 19 | GACTTTATTGATTGCTTCCTGATCAAAATGGAGAAGGAAAAGCAAAACCAACAGTCTGAATTCACTATTGAAAACTTGGTAATCACTGCAGCTGACTTAC | cytochrome P450 family 2 subfamily C member 19 |
| CYP2C8 | NM_000770.3 | cytochrome P450, subfamily IIC (mephenytoin 4-hydroxylase), polypeptide 8,cytochrome P450, family 2, subfamily C, polypeptide 8 | ACCACTGATACTAAGTTCAGAAACTACCTCATCCCCAAGGGCACAACCATAATGGCATTACTGACTTCCGTGCTACATGATGACAAAGAATTTCCTAATC | cytochrome P450 family 2 subfamily C member 8 |
| CYP2C9 | NM_000771.3 | CYP2C10;cytochrome P450, subfamily IIC (mephenytoin 4-hydroxylase), polypeptide 9,cytochrome P450, family 2, subfamily C, polypeptide 9 | AAGAAGAGCAGATGGCCTGGCTGCTGCTGTGCAGTCCCTGCAGCTCTCTTTCCTCTGGGGCATTATCCATCTTTCACTATCTGTAATGCCTTTTCTCACC | cytochrome P450 family 2 subfamily C member 9 |
| CYP2E1 | NM_000773.3 | CYP2E;cytochrome P450, subfamily IIE (ethanol-inducible), polypeptide 1,cytochrome P450, family 2, subfamily E, polypeptide 1 | AGCCGACATCCTCTTCCGCAAGCATTTTGACTACAATGATGAGAAGTTTCTAAGGCTGATGTATTTGTTTAATGAGAACTTCCACCTACTCAGCACTCCC | cytochrome P450 family 2 subfamily E member 1 |
| CYP2J2 | NM_000775.2 | cytochrome P450, subfamily IIJ (arachidonic acid epoxygenase) polypeptide 2,cytochrome P450, family 2, subfamily J, polypeptide 2 | GAAAAATGGATTGATTATGTCAAGTGGCCAGGCATGGAAGGAGCAAAGAAGGTTCACTCTGACAGCACTAAGGAACTTTGGTTTAGGAAAGAAGAGCTTA | cytochrome P450 family 2 subfamily J member 2 |
| CYP4A11/22 | NM_001010969.3 |  | TCAGGAACGGGTGAAGACATTCCCAAGTGCCTGTCCTTATTGGATATGGGGAGGCAAAGTTCGTGTCCAGCTCTATGACCCTGACTATATGAAGGTGATT | cytochrome P450 family 4 subfamily A member 22 |
| CYP7A1 | NM_000780.3 | CYP7;cytochrome P450, subfamily VIIA (cholesterol 7 alpha-monooxygenase), polypeptide 1,cytochrome P450, family 7, subfamily A, polypeptide 1 | CATACCTGGGCTGTGCTCTGCAATTTGGTGCCAATCCTCTTGAGTTCCTCAGAGCAAATCAAAGGAAACATGGTCATGTTTTTACCTGCAAACTAATGGG | cytochrome P450 family 7 subfamily A member 1 |
| CYP8B1 | NM_004391.2 | cytochrome P450, subfamily VIIIB (sterol 12-alpha-hydroxylase), polypeptide 1,cytochrome P450, family 8, subfamily B, polypeptide 1 | TTTCCATCTGCCCTGGGAGGTTCTTTGCACTCAGTGAGGTGAAGCTCTTTATCCTGCTTATGGTCACACACTTTGACTTAGAGTTGGTGGACCCTGACAC | cytochrome P450 family 8 subfamily B member 1 |
| DAPK1 | NM_001288729.1 | death-associated protein kinase 1 | GAGCCGACCTTAATGCTTGCGACAAGGACGGACACATTGCCCTTCATCTGGCTGTAAGACGGTGTCAGATGGAGGTAATCAAGACTCTCCTCAGCCAAGG | death associated protein kinase 1 |
| DAXX | NM_001350.3 | death-associated protein 6 | CTCTTCCAGGAAGCAATCAGAGGAGCCCTTCACCACTGTCTTAGAGAATGGAGCAGGCATGGTCTCTTCTACTTCCTTCAATGGAGGCGTCTCTCCTCAC | death domain associated protein |
| DDIT3 | NM_004083.4 | DNA-damage-inducible transcript 3 | TTAAAGATGAGCGGGTGGCAGCGACAGAGCCAAAATCAGAGCTGGAACCTGAGGAGAGAGTGTTCAAGAAGGAAGTGTATCTTCATACATCACCACACCT | DNA damage inducible transcript 3 |
| DDR2 | NM_006182.2 | TYRO10,NTRKR3;discoidin domain receptor family, member 2 | TGACAGTCAGCCTTTCCCTGCCAAGTGATTCTAGCATGTTCAACAATAACCGCTCCTCATCACCTAGTGAACAAGGGTCCAACTCGACTTACGATCGCAT | discoidin domain receptor tyrosine kinase 2 |
| DEPDC5 | NM_001136029.2 |  | CCAACGATGAATACAGCCCTCTGCTTTTGCAGGTCAAGTCTCTTAAGGAAGATTTACAGAAGGAAACTATCAGTGTGGACCAGACTGTGACTCAAGTGTT | DEP domain containing 5 |
| DERL1 | NM_001134671.2 | Der1-like domain family, member 1 | GAGTAGTTGGGTTGCTTTGTGTTAGGAGGATCCAGATCATGTTGGCTACAGGGAGATGCTCTCTTTGAGAGGCTCCTGGGCATTGATTCCATTTCAATCT | derlin 1 |
| DERL2 | NM_016041.3 | Der1-like domain family, member 2 | AACTCCAGAAGCACTTTATGAAATGGTACACTGACTAATCCAGAAGACATTTCCAACAGTTTGCCAGTGGTTCCTCACTACACTGGTACTGAAAGTGTAA | derlin 2 |
| DIAPH1 | NM_005219.4 | DFNA1;diaphanous (Drosophila, homolog) 1,diaphanous homolog 1 (Drosophila) | GTCCGAAATGACTATGAGGCCAGACCTCAGTACTATAAGTTGATTGAAGAATGTATTTCCCAGATAGTTCTGCACAAGAACGGGGCTGATCCTGACTTCA | diaphanous related formin 1 |
| DLL1 | NM_005618.3 | delta (Drosophila)-like 1,delta-like 1 (Drosophila) | ACCAGTCGGTGTACGTCATATCCGAGGAGAAGGATGAGTGCGTCATAGCAACTGAGGTGTAAAATGGAAGTGAGATGGCAAGACTCCCGTTTCTCTTAAA | delta like canonical Notch ligand 1 |
| DLL4 | NM_019074.2 | delta-like 4 homolog (Drosophila),delta-like 4 (Drosophila) | AATGACCACTTCGGCCACTATGTGTGCCAGCCAGATGGCAACTTGTCCTGCCTGCCCGGTTGGACTGGGGAATATTGCCAACAGCCTATCTGTCTTTCGG | delta like canonical Notch ligand 4 |
| DOCK1 | NM_001290223.1 | dedicator of cyto-kinesis 1 | TCCAGAAACTCTACTGCTTGATCGAAATCGTCCACAGTGACCTCTTCACACAGCATGACTGCAGAGAGATCCTGCTTCCCATGATGACCGATCAGCTCAA | dedicator of cytokinesis 1 |
| DOCK2 | NM_004946.2 | dedicator of cyto-kinesis 2 | AATACAAAACTACCATCCTTTTGCAGGTGGCGGCTTTGAAATACATCCCATCTGTCCTGCATGATGTAGAAATGGTCTTTGATGCGAAGTTACTCAGCCA | dedicator of cytokinesis 2 |
| DUSP7 | NM_001947.2 |  | CTAAGCAGCCCGTGCGACAACCACGCGTCGAGTGAGCAGCTCTACTTTTCCACGCCCACCAACCACAACCTGTTCCCACTCAATACGCTGGAGTCCACGT | dual specificity phosphatase 7 |
| DUSP8 | NM_004420.2 | C11orf81;chromosome 11 open reading frame 81 | GACAACTACTGTGAAAAACTGCTGCCCTGGCTGGACAAGTCCATCGAGTTCATCGATAAAGCCAAGCTCTCCAGCTGCCAAGTCATCGTCCACTGTCTGG | dual specificity phosphatase 8 |
| DYNLL1 | NM_003746.2 | DNCL1;dynein, cytoplasmic, light polypeptide 1 | GCCATTCTTCTGTTCAAATCTGGTTAAAAGCATGGACTGTGCCACACACCCAGTGATCCATCCAAAAACAAGGACTGCAGCCTAAATTCCAAATACCAGA | dynein light chain LC8-type 1 |
| E2F4 | NM_001950.3 | E2F transcription factor 4, p107/p130-binding | GCCAGAAGCGGCGGATTTACGACATTACCAATGTTTTGGAAGGTATCGGGCTAATCGAGAAAAAGTCCAAGAACAGCATCCAGTGGAAGGGTGTGGGGCC | E2F transcription factor 4 |
| EEF2K | NM_013302.3 |  | ATTTTAGGCTTTGCAGGCCATATAGCCTCTGCTGCAAATGCTCAGCCCTGCTGTTGTAATGTAAAAGCTGCCACAGACACTACATGAACACGAATGAGTG | eukaryotic elongation factor 2 kinase |
| EGFR | NM_201282.1 | ERBB;epidermal growth factor receptor (avian erythroblastic leukemia viral (v-erb-b) oncogene homolog) | ACATCCTGCCGGTGGCATTTAGGGGTGACTCCTTCACACATACTCCTCCTCTGGATCCACAGGAACTGGATATTCTGAAAACCGTAAAGGAAATCACAGG | epidermal growth factor receptor |
| EGR1 | NM_001964.2 |  | GAGGCATACCAAGATCCACTTGCGGCAGAAGGACAAGAAAGCAGACAAAAGTGTTGTGGCCTCTTCGGCCACCTCCTCTCTCTCTTCCTACCCGTCCCCG | early growth response 1 |
| EHMT1 | NM_024757.3 | EHMT1-IT1;euchromatic histone methyltransferase 1,EHMT1 intronic transcript 1 | GGCACCAACACACTAACTCGGATAGCGGAAAATGGGGTTTCAGAAAGAGACTCAGAAGCGGCGAAGCAAAACCACGTCACTGCCGACGACTTTGTGCAGA | euchromatic histone lysine methyltransferase 1 |
| ELN | NM_001081754.1 |  | CCACCGTTGGCTGCCATCCAGTTGGTACCCAAGCACCTGAAGCCTCAAAGCTGGATTCGCTCTAGCATCCCTCCTCTCCTGGGTCCACTTGGCCGTCTCC | elastin |
| ELOVL6 | NM_001130721.1 | ELOVL family member 6, elongation of long chain fatty acids (FEN1/Elo2, SUR4/Elo3-like, yeast) | GCAAACACAAAACCCAAGGCAGCTTAGGGATAATTAGGTTGATTTAACCCAGTAAGTTTATGATCCTTTTAGGGTGAGGACTCACTGAGTGCACCTCCAT | ELOVL fatty acid elongase 6 |
| EOMES | NM_005442.2 | eomesodermin (Xenopus laevis) homolog | ATCCCATGCCCTGGGGTATTACCCAGACCCAACCTTTCCTGCAATGGCAGGGTGGGGAGGTCGAGGTTCTTACCAGAGGAAGATGGCAGCTGGACTACCA | eomesodermin |
| EP300 | NM_001429.2 |  | CCAGCCAGGCCCAACAGAGCAGTCCTGGATTAGGTTTGATAAATAGCATGGTCAAAAGCCCAATGACACAGGCAGGCTTGACTTCTCCCAACATGGGGAT | E1A binding protein p300 |
| EP400 | NM_015409.3 | TNRC12;trinucleotide repeat containing 12 | CCTGCACAGCTGGCCCTCCACGTTCCCACACCTGGAAAGGTGCAGGTGCAGGCCTCTCAGCTTTCCTCCCTGCCACAGATGGTAGCATCGACAAGGCTCC | E1A binding protein p400 |
| EPAS1 | NM_001430.3 |  | TGCACTGAGCTATGTGACTCGGATGGTCTTTCACACGGCACATTTGGACATTTCCAGAACTACCATGAGATGGTTTAGACGGGAATTCATGCAAATGAGG | endothelial PAS domain protein 1 |
| EPHX2 | NM_001979.5 | epoxide hydrolase 2, cytoplasmic | AGGAAGTGCTCCGAGACCGCTAAAGTCTGCCTCCCCAAGAATTTCTCCATAAAAGAAATCTTTGACAAGGCGATTTCAGCCAGAAAGATCAACCGCCCCA | epoxide hydrolase 2 |
| ERC1 | NM_178040.1 | RAB6IP2;RAB6 interacting protein 2 | TGACATGAGAATGAAACCAGGAATGGACTTGGAGTTCAACAGGCTGAGAGGATGCCTCCAATGGACCAGAGAGCTGAGTGTTCTAATATCACAATAGGTG | ELKS/RAB6-interacting/CAST family member 1 |
| ERN1 | NM_001433.2 | ER to nucleus signalling 1 | TACATGGGTAAAAAGCAGGACATCTGGTATGTTATTGACCTCCTGACCGGAGAGAAGCAGCAGACTTTGTCATCGGCCTTTGCAGATAGTCTCTGCCCAT | endoplasmic reticulum to nucleus signaling 1 |
| ERO1A | NM_014584.1 | ERO1L;ERO1 (S. cerevisiae)-like,ERO1-like (S. cerevisiae) | AGAATGTGAACAAGCTGAACGACTTGGAGCAGTGGATGAATCTCTGAGTGAGGAAACACAGAAGGCTGTTCTTCAGTGGACCAAGCATGATGATTCTTCA | endoplasmic reticulum oxidoreductase 1 alpha |
| ERP29 | NM_001034025.1 | C12orf8;chromosome 12 open reading frame 8 | TAACCTGCTGGCTGTGAGTCCCTTGTGGAATATAAGGGGGTAGTGGGAAAAGTGGTACTAACCCACGATTCTGAGCCCTGAGTATGCCTGGACATTGATG | endoplasmic reticulum protein 29 |
| F11R | NM_144503.1 | JAM1;junctional adhesion molecule 1 | GGAAGCAGAGGTGATTCATGGCTCTGTGAATTTGAGGTGAATGGTTCCTTATTGTCTAGGCCACTTGTGAAGAATATGAGTCAGTTATTGCCAGCCTTGG | F11 receptor |
| F5 | NM_000130.2 | coagulation factor V (proaccelerin, labile factor) | GAGACTGTAGGATGCCAATGGGACTAAGCACTGGTATCATATCTGATTCACAGATCAAGGCTTCAGAGTTTCTGGGTTACTGGGAGCCCAGATTAGCAAG | coagulation factor V |
| F8 | NM_019863.2 | F8C;coagulation factor VIII, procoagulant component | CCATTGGGAATGGAGAGTAAAGCAATATCAGATGCACAGATTACTGCTTCATCCTACTTTACCAATATGTTTGCCACCTGGTCTCCTTCAAAAGCTCGAC | coagulation factor VIII |
| FABP4 | NM_001442.2 |  | GGTGGAATGCGTCATGAAAGGCGTCACTTCCACGAGAGTTTATGAGAGAGCATAAGCCAAGGGACGTTGACCTGGACTGAAGTTCGCATTGAACTCTACA | fatty acid binding protein 4 |
| FABP5 | NM_001444.1 | fatty acid binding protein 5 (psoriasis-associated) | GCTTTGATGAATACATGAAGGAGCTAGGAGTGGGAATAGCTTTGCGAAAAATGGGCGCAATGGCCAAGCCAGATTGTATCATCACTTGTGATGGTAAAAA | fatty acid binding protein 5 |
| FADD | NM_003824.2 | Fas (TNFRSF6)-associated via death domain | TGAGACTGCTAAGTAGGGGCAGTGATGGTTGCCAGGACGAATTGAGATAATATCTGTGAGGTGCTGATGAGTGATTGACACACAGCACTCTCTAAATCTT | Fas associated via death domain |
| FAM30A | NR_026800.2 | C14orf110,KIAA0125;chromosome 14 open reading frame 110,KIAA0125 | ACAGTTCTGAAGTCAAAGGCTGATGTCCTGTTTCTCTTTCCCTCTGTGACCGACTCCCTTCCCAGTGGTAACAAGTACCCACAGCTTGGTTTGAATTTCT | family with sequence similarity 30 member A |
| FAP | NM_004460.2 | fibroblast activation protein, alpha | GCATTGGAAGCTATCCTCCAAGCAAGAAGTGTGTTACTTGCCATCTAAGGAAAGAAAGGTGCCAATATTACACAGCAAGTTTCAGCGACTACGCCAAGTA | fibroblast activation protein alpha |
| FAS | NM_000043.4 | FAS1,APT1,TNFRSF6;tumor necrosis factor receptor superfamily, member 6,Fas (TNF receptor superfamily, member 6) | TATCGTCCAAAAGTGTTAATGCCCAAGTGACTGACATCAACTCCAAGGGATTGGAATTGAGGAAGACTGTTACTACAGTTGAGACTCAGAACTTGGAAGG | Fas cell surface death receptor |
| FASN | NM_004104.4 |  | GAGGTGCTTGGCTACGCACGGTCGCTTCCTGGAAATTGGCAAATTCGACCTTTCTCAGAACCACCCGCTCGGCATGGCTATCTTCCTGAAGAACGTGACA | fatty acid synthase |
| FBP1 | NM_000507.3 | FBP;fructose-1,6-bisphosphatase 1 | AGTCATCCTTTGCCACGTGTGTTCTCGTGTCAGAAGAAGATAAACACGCCATCATAGTGGAACCGGAGAAAAGGGGTAAATATGTGGTCTGTTTTGATCC | fructose-bisphosphatase 1 |
| FCAR | NM_002000.2 | Fc fragment of IgA, receptor for | TGCTGAGATTATAGGCATGAGCCACCACGCCTGGCCAGATGCATGTTCAAACCAATCAAATGGTGTTTTCTTATGCAGGACTGATCGATTTGCACCCACC | Fc fragment of IgA receptor |
| FCER1A | NM_002001.2 | FCE1A;Fc fragment of IgE, high affinity I, receptor for; alpha polypeptide | GAATCCCCTACTCTACTGTGTGTAGCCTTACTGTTCTTCGCTCCAGATGGCGTGTTAGCAGTCCCTCAGAAACCTAAGGTCTCCTTGAACCCTCCATGGA | Fc fragment of IgE receptor Ia |
| FCER1G | NM_004106.1 | Fc fragment of IgE, high affinity I, receptor for; gamma polypeptide | AGTGGTCTTGCTCTTACTCCTTTTGGTTGAACAAGCAGCGGCCCTGGGAGAGCCTCAGCTCTGCTATATCCTGGATGCCATCCTGTTTCTGTATGGAATT | Fc fragment of IgE receptor Ig |
| FCGR3A/B | NM_000570.4 | FCGR3,FCG3;Fc fragment of IgG, low affinity IIIb, receptor for (CD16),Fc fragment of IgG, low affinity IIIb, receptor (CD16b) | AAGGTTTGGCAGTGTCAACCATCTCATCATTCTCTCCACCTGGGTACCAAGTCTCTTTCTGCTTGGTGATGGTACTCCTTTTTGCAGTGGACACAGGACT | Fc fragment of IgG receptor IIIb |
| FCRL2 | NM_001159488.1 | SPAP1;SH2 domain-containing phosphatase anchor protein 1,Fc receptor-like 2 | CCTCTTCTGTCTTCGAAGGAGACAGCATCGTTCTGAAATGCCAGGGAGAACAGAACTGGAAAATTCAGAAGATGGCTTACCATAAGGATAACAAAGAGTT | Fc receptor like 2 |
| FGA | NM_021871.2 | fibrinogen, A alpha polypeptide | GAGAGGCGATTTTTCCTCAGCCAATAACCGTGATAATACCTACAACCGAGTGTCAGAGGATCTGAGAAGCAGAATTGAAGTCCTGAAGCGCAAAGTCATA | fibrinogen alpha chain |
| FGB | NM_005141.3 | fibrinogen, B beta polypeptide | CAACACAGATGGGAAGAATTACTGTGGCCTACCAGGTGAATATTGGCTTGGAAATGATAAAATTAGCCAGCTTACCAGGATGGGACCCACAGAACTTTTG | fibrinogen beta chain |
| FGD2 | NM_173558.3 | FGD1 family, member 2 | TCTAACACCCTGCTCCGTGAGGGCCCGGTCCTCAAGATCTCCTTCCGCCGCAACGACCCCATGGAGCGCTACCTTTTCTTGTTCAACAACATGCTGCTCT | FYVE, RhoGEF and PH domain containing 2 |
| FGF19 | NM_005117.2 |  | GATGCAGGGGCTGCTTCAGTACTCGGAGGAAGACTGTGCTTTCGAGGAGGAGATCCGCCCAGATGGCTACAATGTGTACCGATCCGAGAAGCACCGCCTC | fibroblast growth factor 19 |
| FGF2 | NM_002006.4 | FGFB;fibroblast growth factor 2 (basic) | GTCCGGGAGAAGAGCGACCCTCACATCAAGCTACAACTTCAAGCAGAAGAGAGAGGAGTTGTGTCTATCAAAGGAGTGTGTGCTAACCGTTACCTGGCTA | fibroblast growth factor 2 |
| FGF21 | NM_019113.2 |  | GATCACCTGAGGACCCGAGCCATTGATGGACTCGGACGAGACCGGGTTCGAGCACTCAGGACTGTGGGTTTCTGTGCTGGCTGGTCTTCTGCTGGGAGCC | fibroblast growth factor 21 |
| FGG | NM_000509.4 | fibrinogen, gamma polypeptide | GGTGGGGATGCTGGAGATGCCTTTGATGGCTTTGATTTTGGCGATGATCCTAGTGACAAGTTTTTCACATCCCATAATGGCATGCAGTTCAGTACCTGGG | fibrinogen gamma chain |
| FGL2 | NM_006682.2 | fibrinogen-like 2 | CAATTCAGCAGGATCGAGGAGGTGTTCAAAGAAGTCCAAAACCTCAAGGAAATCGTAAATAGTCTAAAGAAATCTTGCCAAGACTGCAAGCTGCAGGCTG | fibrinogen like 2 |
| FLI1 | NM_001167681.2 | Friend leukemia virus integration 1 | GGGCCCTCCGTTATTACTATGATAAAAACATTATGACCAAAGTGCACGGCAAAAGATATGCTTACAAATTTGACTTCCACGGCATTGCCCAGGCTCTGCA | Fli-1 proto-oncogene, ETS transcription factor |
| FLNB | NM_001164317.1 | FLN1L,LRS1;filamin B, beta (actin binding protein 278),Larsen syndrome 1 (autosomal dominant),filamin B, beta | GGTTTGGAGAAATCTGGATGCATTGTCAACAACCTGGCCGAGTTCACTGTGGATCCTAAGGATGCTGGAAAAGCTCCCTTAAAGATATTTGCTCAGGATG | filamin B |
| FLT1 | NM_002019.4 | FLT;fms-related tyrosine kinase 1 (vascular endothelial growth factor/vascular permeability factor receptor),fms-related tyrosine kinase 1 | AAGAAATGGCAAACAATTCTGCAGTACTTTAACCTTGAACACAGCTCAAGCAAACCACACTGGCTTCTACAGCTGCAAATATCTAGCTGTACCTACTTCA | fms related tyrosine kinase 1 |
| FLT4 | NM_002020.1 | fms-related tyrosine kinase 4 | CCAACAACGGCATCCAGCGATTTCGGGAGAGCACCGAGGTCATTGTGCATGAAAATCCCTTCATCAGCGTCGAGTGGCTCAAAGGACCCATCCTGGAGGC | fms related tyrosine kinase 4 |
| FN1 | NM_212482.1 |  | GGGAATGGACATGCATTGCCTACTCGCAGCTTCGAGATCAGTGCATTGTTGATGACATCACTTACAATGTGAACGACACATTCCACAAGCGTCATGAAGA | fibronectin 1 |
| FNIP2 | NM_020840.1 |  | TACTTTATGTCCTGACCTACTTTCTCCGTTGCTCTGAGCTACAAGAGAACCAGCTGACCTGGAGTGGCAATCATGGTGAAGGTGACCAAGTTTTAAATGG | folliculin interacting protein 2 |
| FOXP3 | NM_014009.3 | IPEX;immune dysregulation, polyendocrinopathy, enteropathy, X-linked | GGGCCATCCTGGAGGCTCCAGAGAAGCAGCGGACACTCAATGAGATCTACCACTGGTTCACACGCATGTTTGCCTTCTTCAGAAACCATCCTGCCACCTG | forkhead box P3 |
| FPR1 | NM_002029.3 |  | GCCATGGGAGGACATTGGCCTTTCGGCTGGTTCCTGTGCAAATTCGTCTTTACCATAGTGGACATCAACTTGTTCGGAAGTGTCTTCCTGATCGCCCTCA | formyl peptide receptor 1 |
| FRS2 | NM_006654.3 |  | ATTTGGAGATGCTTCATCCCATCCGTCAAGCAGACATCCTTCTGTGGGAAGTGCTCGCCTGCCTTCAGTAGGGGAAGAATCTACACATCCTTTGCTTGTG | fibroblast growth factor receptor substrate 2 |
| FST | NM_006350.2 |  | GACCAATAATGCCTACTGTGTGACCTGTAATCGGATTTGCCCAGAGCCTGCTTCCTCTGAGCAATATCTCTGTGGGAATGATGGAGTCACCTACTCCAGT | follistatin |
| FURIN | NM_002569.2 | PCSK3,FUR,PACE;paired basic amino acid cleaving enzyme (furin, membrane associated receptor protein),furin (paired basic amino acid cleaving enzyme) | GGGTCCCAGTGGGAGGGGCAGGCTGACATCTGTGTTTCAAGTGGGGCTCGCCATGCCGGGGGTTCATAGGTCACTGGCTCTCCAAGTGCCAGAGGTGGGC | furin, paired basic amino acid cleaving enzyme |
| FZD2 | NM_001466.2 | frizzled (Drosophila) homolog 2,frizzled homolog 2 (Drosophila),frizzled 2, seven transmembrane spanning receptor,frizzled family receptor 2 | GGAGGAGACGCGTTTCGCGCGCCTCTGGATCCTCACCTGGTCGGTGCTGTGCTGCGCTTCCACCTTCTTCACTGTCACCACGTACTTGGTAGACATGCAG | frizzled class receptor 2 |
| FZD5 | NM_003468.2 | C2orf31;frizzled (Drosophila) homolog 5,chromosome 2 open reading frame 31,frizzled homolog 5 (Drosophila),frizzled 5, seven transmembrane spanning receptor,frizzled family receptor 5 | GAGAGAGGGAAGAGGGGCGTTTTCGAGGAAGAACCTGTCCCAGGTCTTCTCCAAGGGGCCCAGCTCACGTGTATTCTATTTTGCGTTTCTTACTGCCTTC | frizzled class receptor 5 |
| GAB1 | NM_002039.3 |  | TTTGTGACATCTGTGGGTTTAATCCAACAGAAGAAGATCCTGTGAAGCCACCTGGCAGCTCTTTACAAGCACCAGCTGATTTACCTTTAGCTATAAATAC | GRB2 associated binding protein 1 |
| GAS1 | NM_002048.2 |  | CTGTGGCTTGGGACAGATAGAAGGGATGGTTGGGGATACTTCCCAAAACTTTTTCCAAGTCAACTTGGTGTAGCCGGTTCCCCGGCCACGACTCTGGGCA | growth arrest specific 1 |
| GATA3 | NM_001002295.1 | GATA-binding protein 3 | GTGCATGACTCACTGGAGGACTTCCCCAAGAACAGCTCGTTTAACCCGGCCGCCCTCTCCAGACACATGTCCTCCCTGAGCCACATCTCGCCCTTCAGCC | GATA binding protein 3 |
| GBP4 | NM_052941.4 |  | TTCTACAAGATATGCCATGGGCCTTTTCACAGGGGACACAGGCTTCTTAAAACAACCCGGCTTCCTCACCCTATGTCCTTTATTTACAAAGCTGTGCTCC | guanylate binding protein 4 |
| GBP5 | NM_052942.3 |  | TTCTGCTTAGGCCTGGAAATAGATGGGCAACTTGTCACACCAGATGAATACCTGGAGAATTCCCTAAGGCCAAAGCAAGGTAGTGATCAAAGAGTTCAAA | guanylate binding protein 5 |
| GCNT1 | NM_001097633.1 | NACGT2;glucosaminyl (N-acetyl) transferase 1, core 2 (beta-1,6-N-acetylglucosaminyltransferase),glucosaminyl (N-acetyl) transferase 1, core 2 | GGGACTGTCAAAATGCTTCCTCCACTCGAAACACCTCTCTTTTCTGGCAGTGCCTACTTCGTGGTCAGTAGGGAGTATGTGGGGTATGTACTACAGAATG | glucosaminyl (N-acetyl) transferase 1 |
| GLI1 | NM_005269.1 | GLI;glioma-associated oncogene homolog 1 (zinc finger protein),glioma-associated oncogene family zinc finger 1 | TAGCCCAAGCCGTGCTAAAGCTCCAGTGAACACATATGGACCTGGCTTTGGACCCAACTTGCCCAATCACAAGTCAGGTTCCTATCCCACCCCTTCACCA | GLI family zinc finger 1 |
| GLI2 | NM_005270.4 | GLI-Kruppel family member GLI2,glioma-associated oncogene family zinc finger 2 | CCAGCTGCGCAAACACATGACCACCATGCACCGGTTCGAGCAGCTCAAGAAGGAGAAGCTCAAGTCACTCAAGGATTCCTGCTCATGGGCCGGGCCGACT | GLI family zinc finger 2 |
| GLI3 | NM_000168.5 | GCPS,PHS;Greig cephalopolysyndactyly syndrome,GLI-Kruppel family member GLI3,glioma-associated oncogene family zinc finger 3 | CATATCACCACTCTCCGATCATAGCTTTGACCTTCAGACCATGATAAGGACGTCTCCCAACTCCTTGGTCACGATTCTCAATAATTCCCGTAGCAGCTCT | GLI family zinc finger 3 |
| GLP1R | NM_002062.3 | glucagon-like peptide 1 receptor | CCTGTTCCTCTACATCATCTACACGGTGGGCTACGCACTCTCCTTCTCTGCTCTGGTTATCGCCTCTGCGATCCTCCTCGGCTTCAGACACCTGCACTGC | glucagon like peptide 1 receptor |
| GNB4 | NM_021629.3 | guanine nucleotide binding protein (G protein), beta polypeptide 4 | TAGATGACAGCCAAATTGTTACAAGTTCAGGAGATACAACTTGTGCTTTATGGGACATCGAAACTGCCCAGCAGACCACCACATTCACTGGGCATTCTGG | G protein subunit beta 4 |
| GNG2 | NM_053064.4 | guanine nucleotide binding protein (G protein), gamma 2 | GTCACCAAGCAGGAGAGTTAAGGATTCACCATGAGCTGGGAAATGCTTTTGCCATGAGTATGAGCAAATTCCCTCTTTCCCTGAATCATGGACATTCTAG | G protein subunit gamma 2 |
| GNLY | NM_012483.2 | LAG2 | TGCCGGCTCCTCGCTTCCTCGATCCAGAATCCACTCTCCAGTCTCCCTCCCCTGACTCCCTCTGCTGTCCTCCCCTCTCACGAGAATAAAGTGTCAAGCA | granulysin |
| GNPTAB | NM_024312.3 | GNPTA;N-acetylglucosamine-1-phosphate transferase, alpha and beta subunits | AACAGATGAAACAAATGACAGTTTGGTGGCTCCACAGGAAAAACAGGTTCATAAAAGCATCTTGCCAAACAGCTTAGGAGTGTCTGAAAGATTGCAGAGG | N-acetylglucosamine-1-phosphate transferase subunits alpha and beta |
| GOT2 | NM_002080.2 | glutamic-oxaloacetic transaminase 2, mitochondrial (aspartate aminotransferase 2),glutamic-oxaloacetic transaminase 2, mitochondrial | GGAGAGTAGGAAACTGTACTTTATCTCGGCATCCTCTTGAATGATAGTGCAAGTTTCTCCAGTTGGGATGTTGTCTCTGCCCGGTTGGACCTCCTCCCTT | glutamic-oxaloacetic transaminase 2 |
| GPC4 | NM_001448.2 |  | CCCTCGCAAATTGAAGCTCCAGGTTACTCGTGCTTTTGTAGCAGCCCGTACTTTCGCTCAAGGCTTAGCGGTTGCGGGAGATGTCGTGAGCAAGGTCTCC | glypican 4 |
| GPR65 | NM_003608.2 |  | GTACAGGCTATGCAATACCTTTGGTCACCATCCTGATCTGCAACCGGAAAGTCTACCAAGCTGTGCGGCACAATAAAGCCACGGAAAACAAGGAAAAGAA | G protein-coupled receptor 65 |
| GPX2 | NM_002083.2 | glutathione peroxidase 2 (gastrointestinal) | CCTCTGGTTGGTGATTCAACTTGGGCTCCAAGACTTGGGTAAGCTCTGGGCCTTCACAGAATGATGGCACCTTCCTAAACCCTCATGGGTGGTGTCTGAG | glutathione peroxidase 2 |
| GPX3 | NM_002084.3 | glutathione peroxidase 3 (plasma) | GTCCAATTGTTCTGCTCTAACTGATACCTCAACCTTGGGGCCAGCATCTCCCACTGCCTCCAAATATTAGTAACTATGACTGACGTCCCCAGAAGTTTCT | glutathione peroxidase 3 |
| GRB10 | NM_001001555.2 | growth factor receptor-bound protein 10 | TCTGCACTTAATACCTGACAGTATGACCGATCTCTGCGCCTTTCTGGGGGCGGGCAAGCTGGCGGTAGATTTGTGATGTCACAGTGCAAACTGCAGTGAC | growth factor receptor bound protein 10 |
| GREM1 | NM_013372.5 | CKTSF1B1,CRAC1;cysteine knot superfamily 1, BMP antagonist 1,gremlin 1, cysteine knot superfamily, homolog (Xenopus laevis),gremlin 1,colorectal adenoma and carcinoma 1 | GTCCTGCTCCTTCTGCAAGCCCAAGAAATTCACTACCATGATGGTCACACTCAACTGCCCTGAACTACAGCCACCTACCAAGAAGAAGAGAGTCACACGT | gremlin 1, DAN family BMP antagonist |
| GSK3B | NM_002093.2 |  | ACTGATTATACCTCTAGTATAGATGTATGGTCTGCTGGCTGTGTGTTGGCTGAGCTGTTACTAGGACAACCAATATTTCCAGGGGATAGTGGTGTGGATC | glycogen synthase kinase 3 beta |
| GZMA | NM_006144.2 | HFSP,CTLA3;granzyme A (granzyme 1, cytotoxic T-lymphocyte-associated serine esterase 3) | AGACCCTACATGGTCCTACTTAGTCTTGACAGAAAAACCATCTGTGCTGGGGCTTTGATTGCAAAAGACTGGGTGTTGACTGCAGCTCACTGTAACTTGA | granzyme A |
| GZMB | NM_004131.3 | CTLA1,CSPB;granzyme B (granzyme 2, cytotoxic T-lymphocyte-associated serine esterase 1) | ACACTACAAGAGGTGAAGATGACAGTGCAGGAAGATCGAAAGTGCGAATCTGACTTACGCCATTATTACGACAGTACCATTGAGTTGTGCGTGGGGGACC | granzyme B |
| GZMH | NM_033423.3 | CTSGL2;granzyme H (cathepsin G-like 2, protein h-CCPX) | AAAAAAGGGACACCTCCAGGAGTCTACATCAAGGTCTCACACTTCCTGCCCTGGATAAAGAGAACAATGAAGCGCCTCTAACAGCAGGCATGAGACTAAC | granzyme H |
| HADH | NM_001184705.2 | HADHSC;L-3-hydroxyacyl-Coenzyme A dehydrogenase, short chain,hydroxyacyl-Coenzyme A dehydrogenase | ATCTTTGCCAGCAACACTTCCTCCTTGCAGATTACAAGCATAGCTAATGCCACCACCAGACAAGACCGATTCGCTGGCCTCCATTTCTTCAACCCAGTGC | hydroxyacyl-CoA dehydrogenase |
| HADHA | NM_000182.4 | hydroxyacyl-Coenzyme A dehydrogenase/3-ketoacyl-Coenzyme A thiolase/enoyl-Coenzyme A hydratase (trifunctional protein), alpha subunit,hydroxyacyl-CoA dehydrogenase/3-ketoacyl-CoA thiolase/enoyl-CoA hydratase (trifunctional protein), alpha subunit | CCATCTTCAGCAACTTGACTGGGCAGCTTGATTACCAAGGTTTTGAAAAGGCCGACATGGTGATTGAAGCTGTGTTTGAGGACCTTAGTCTTAAGCACAG | hydroxyacyl-CoA dehydrogenase trifunctional multienzyme complex subunit alpha |
| HAVCR1 | NM_001173393.1 |  | GGGAATGACACCGTGACAGAGTCTTCAGATGGCCTTTGGAATAACAATCAAACTCAACTGTTCCTAGAACATAGTCTACTGACGGCCAATACCACTAAAG | hepatitis A virus cellular receptor 1 |
| HCAR2 | NM_177551.3 | GPR109A;G protein-coupled receptor 109A | CTAGGCATCTGAAACTTTTGCTTCATCTCTGACGCTCGCAGGACTGAAGATGGGCAAATTGTAGGCATTTCTGCTGAGCAGAGTTGGAGCCAGAGATCTA | hydroxycarboxylic acid receptor 2 |
| HCFC1 | NM_005334.2 | HFC1,MRX3;mental retardation, X-linked 3,host cell factor C1 (VP16-accessory protein) | AACCAGTGGTTTGATGTGGGAGTCATTAAGGGCACCAATGTAATGGTGACACACTATTTCCTGCCACCAGATGATGCTGTCCCATCAGACGATGATTTGG | host cell factor C1 |
| HCK | NM_002110.2 | hemopoietic cell kinase | CCAGGTCGGAGGCAATACATTCTCAAAAACTGAAACCAGCGCCAGCCCACACTGTCCTGTGTACGTGCCGGATCCCACATCCACCATCAAGCCGGGGCCT | HCK proto-oncogene, Src family tyrosine kinase |
| HDAC4 | NM_006037.3 | BDMR;brachydactyly-mental retardation syndrome | TCTGTCAGCTCACTCCAGCTTCACAAATGTGCTGAGAGCATTACTGTGTAGCCTTTTCTTTGAAGACACACTCGGCTCTTCTCCACAGCAAGCGTCCAGG | histone deacetylase 4 |
| HDC | NM_002112.3 |  | GCAGGCACTGCCTTCCTGTGCCCCGAGTTCCGGGGGTTTCTGAAGGGGATTGAGTATGCCGACTCCTTCACCTTTAATCCTTCCAAGTGGATGATGGTGC | histidine decarboxylase |
| HIF1A | NM_001530.2 | hypoxia inducible factor 1, alpha subunit (basic helix-loop-helix transcription factor) | ATGGATGATGACTTCCAGTTACGTTCCTTCGATCAGTTGTCACCATTAGAAAGCAGTTCCGCAAGCCCTGAAAGCGCAAGTCCTCAAAGCACAGTTACAG | hypoxia inducible factor 1 subunit alpha |
| HIF3A | NM_152796.2 |  | ACCTACTGTGACGACAGGATTGCAGAAGTGGCTGGCTATAGTCCCGATGACCTGATCGGCTGTTCCGCCTACGAGTACATCCACGCGCTGGACTCCGATG | hypoxia inducible factor 3 subunit alpha |
| HIKESHI | NM_016401.4 | C11orf73;chromosome 11 open reading frame 73 | TGGAGCCATGAATATTGTCCGAACTCCATCTGTTGCTCAGATTGGAATTTCAGTGGAATTATTAGACAGTATGGCTCAGCAGACTCCTGTAGGTAATGCT | heat shock protein nuclear import factor hikeshi |
| HKDC1 | NM_025130.3 |  | AGTTTGACCTGGACATTGTTGCAGTCGTGAATGATACAGTGGGGACCATGATGACCTGTGGCTATGAAGATCCTAATTGTGAGATTGGCCTGATTGCAGG | hexokinase domain containing 1 |
| HLA-A | NM_002116.5 |  | GGAAGAGCTCAGATAGAAAAGGAGGGAGTTACACTCAGGCTGCAAGCAGTGACAGTGCCCAGGGCTCTGATGTGTCCCTCACAGCTTGTAAAGTGTGAGA | major histocompatibility complex, class I, A |
| HLA-B | NM_005514.6 | AS;ankylosing spondylitis | CCCTGAGATGGGAGCCGTCTTCCCAGTCCACCGTCCCCATCGTGGGCATTGTTGCTGGCCTGGCTGTCCTAGCAGTTGTGGTCATCGGAGCTGTGGTCGC | major histocompatibility complex, class I, B |
| HLA-DPA1 | NM_033554.2 | HLA-DP1A | GGAGAGATCTGAACTCCAGCTGCCCTACAAACTCCATCTCAGCTTTTCTTCTCACTTCATGTGAAAACTACTCCAGTGGCTGACTGAATTGCTGACCCTT | major histocompatibility complex, class II, DP alpha 1 |
| HLA-DQA1 | NM_002122.3 | HLA-DQA | GGTGGCCTGAGTTCAGCAAATTTGGAGGTTTTGACCCGCAGGGTGCACTGAGAAACATGGCTGTGGCAAAACACAACTTGAACATCATGATTAAACGCTA | major histocompatibility complex, class II, DQ alpha 1 |
| HLA-DQA2 | NM_020056.4 | HLA-DXA | GCAGTTGCCTATGTTTAGCAAATTTATAAGTTTTGACCCGCAGAGTGCACTGAGAAATATGGCTGTGGGAAAACACACCTTGGAATTCATGATGAGACAG | major histocompatibility complex, class II, DQ alpha 2 |
| HLA-DRA | NM_019111.3 | HLA-DRA1 | GGCCAACATAGCTGTGGACAAAGCCAACCTGGAAATCATGACAAAGCGCTCCAACTATACTCCGATCACCAATGTACCTCCAGAGGTAACTGTGCTCACG | major histocompatibility complex, class II, DR alpha |
| HLA-E | NM_005516.4 |  | TGTCTTAGGGGACTCTGGCTTCTCTTTTTGCAAGGGCCTCTGAATCTGTCTGTGTCCCTGTTAGCACAATGTGAGGAGGTAGAGAAACAGTCCACCTCTG | major histocompatibility complex, class I, E |
| HLA-F | NM_001098479.1 |  | TGCGCTCCTGGACCGCGGCGGACACCGTGGCTCAGATCACCCAGCGCTTCTATGAGGCAGAGGAATATGCAGAGGAGTTCAGGACCTACCTGGAGGGCGA | major histocompatibility complex, class I, F |
| HLA-G | NM_002127.4 | HLA-G histocompatibility antigen, class I, G | AAGAGCTCAGATTGAAAAGGAGGGAGCTACTCTCAGGCTGCAATGTGAAACAGCTGCCCTGTGTGGGACTGAGTGGCAAGTCCCTTTGTGACTTCAAGAA | major histocompatibility complex, class I, G |
| HMGCS2 | NM_005518.3 | 3-hydroxy-3-methylglutaryl-Coenzyme A synthase 2 (mitochondrial),3-hydroxy-3-methylglutaryl-CoA synthase 2 (mitochondrial) | CACCAACAAGGACCTGGATAAAGCACTTCTAAAGGCCTCTCAGGACATGTTCGACAAGAAAACCAAGGCTTCCCTTTACCTCTCCACTCACAATGGGAAC | 3-hydroxy-3-methylglutaryl-CoA synthase 2 |
| HMOX1 | NM_002133.2 | heme oxygenase (decycling) 1 | CAAGGACCAGAGCCCCTCACGGGCACCAGGGCTTCGCCAGCGGGCCAGCAACAAAGTGCAAGATTCTGCCCCCGTGGAGACTCCCAGAGGGAAGCCCCCA | heme oxygenase 1 |
| HSBP1 | NM_001537.2 |  | CCATCCGAAACCTGCGTCCCTGGTGATGTTCTCAAGCCTCGGAAGTGGCAAATGGAAATGATATGGCCGGTTGCGGTTGTAGGAGAGTTGTGACTTAGGC | heat shock factor binding protein 1 |
| HSD11B1 | NM_181755.1 | HSD11B,HSD11;hydroxysteroid (11-beta) dehydrogenase 1 | GCCTACTACTACTATTCTGCAAACGAGGAATTCAGACCAGAGATGCTCCAAGGAAAGAAAGTGATTGTCACAGGGGCCAGCAAAGGGATCGGAAGAGAGA | hydroxysteroid 11-beta dehydrogenase 1 |
| HSD17B4 | NM_000414.2 | hydroxysteroid (17-beta) dehydrogenase 4 | GGTTGTGAAGACAGCCCTGGATGCTTTTGGAAGAATAGATGTTGTGGTCAACAATGCTGGAATTCTGAGGGATCGTTCCTTTGCTAGGATAAGTGATGAA | hydroxysteroid 17-beta dehydrogenase 4 |
| HSP90AA1 | NM_001017963.2 | HSPC1,HSPCA;heat shock 90kD protein 1, alpha,heat shock 90kDa protein 1, alpha,heat shock protein 90kDa alpha (cytosolic), class A member 1 | CTTGACCAATGACTGGGAAGATCACTTGGCAGTGAAGCATTTTTCAGTTGAAGGACAGTTGGAATTCAGAGCCCTTCTATTTGTCCCACGACGTGCTCCT | heat shock protein 90 alpha family class A member 1 |
| HSP90AB1 | NM_007355.3 | HSPC2,HSPCB;heat shock 90kD protein 1, beta,heat shock 90kDa protein 1, beta,heat shock protein 90kDa alpha (cytosolic), class B member 1 | GGCATTCTCTAAAAATCTCAAGCTTGGAATCCACGAAGACTCCACTAACCGCCGCCGCCTGTCTGAGCTGCTGCGCTATCATACCTCCCAGTCTGGAGAT | heat shock protein 90 alpha family class B member 1 |
| HSPG2 | NM_005529.5 | SJS1;Schwartz-Jampel syndrome 1 (chondrodystrophic myotonia) | CCGCTGTAAGAACAATGTGGTGGGGCGCTTGTGCAATGAATGTGCTGACGGCTCTTTCCACCTGAGTACCCGAAACCCCGATGGCTGCCTCAAGTGCTTC | heparan sulfate proteoglycan 2 |
| HTRA2 | NM_013247.4 | PRSS25;protease, serine, 25 | GATCCTGGACCGGCACCCTTTCTTGGGCCGCGAGGTCCCTATCTCGAACGGCTCAGGATTCGTGGTGGCTGCCGATGGGCTCATTGTCACCAACGCCCAT | HtrA serine peptidase 2 |
| HVCN1 | NM_032369.3 | hydrogen voltage-gated channel 1 | TGTTCCAGGAGCACCAGTTTGAGGCTCTGGGCCTGCTGATTCTGCTCCGGCTGTGGCGGGTGGCCCGGATCATCAATGGGATTATCATCTCAGTTAAGAC | hydrogen voltage gated channel 1 |
| ICAM1 | NM_000201.2 |  | AAATACTGAAACTTGCTGCCTATTGGGTATGCTGAGGCCCCACAGACTTACAGAAGAAGTGGCCCTCCATAGACATGTGTAGCATCAAAACACAAAGGCC | intercellular adhesion molecule 1 |
| ICOS | NM_012092.2 | inducible T-cell costimulator | AACTCTGGCACCCAGGCATGAAGCACGTTGGCCAGTTTTCCTCAACTTGAAGTGCAAGATTCTCTTATTTCCGGGACCACGGAGAGTCTGACTTAACTAC | inducible T cell costimulator |
| IFI27 | NM_005532.3 |  | TCACTGGGAGCAACTGGACTCTCCGGATTGACCAAGTTCATCCTGGGCTCCATTGGGTCTGCCATTGCGGCTGTCATTGCGAGGTTCTACTAGCTCCCTG | interferon alpha inducible protein 27 |
| IFI35 | NM_005533.3 | interferon-induced protein 35 | TGCCCTCTGCTTGCGGGCTCTGCTCTGATCACCTTTGATGACCCCAAAGTGGCTGAGCAGGTGCTGCAACAAAAGGAGCACACGATCAACATGGAGGAGT | interferon induced protein 35 |
| IFI6 | NM_002038.3 | G1P3;interferon, alpha-inducible protein (clone IFI-6-16) | AGCAGCGTCGTCATAGGTAATATTGGTGCCCTGATGGGCTACGCCACCCACAAGTATCTCGATAGTGAGGAGGATGAGGAGTAGCCAGCAGCTCCCAGAA | interferon alpha inducible protein 6 |
| IFNG | NM_000619.2 |  | ATACTATCCAGTTACTGCCGGTTTGAAAATATGCCTGCAATCTGAGCCAGTGCTTTAATGGCATGTCAGACAGAACTTGAATGTGTCAGGTGACCCTGAT | interferon gamma |
| IFNGR1 | NM_000416.2 | IFNGR | CTCTTTGGGTCAGAGTTAAAGCCAGGGTTGGACAAAAAGAATCTGCCTATGCAAAGTCAGAAGAATTTGCTGTATGCCGAGATGGAAAAATTGGACCACC | interferon gamma receptor 1 |
| IGF1 | NM_000618.3 | insulin-like growth factor 1 (somatomedin C) | CGTGGATGAGTGCTGCTTCCGGAGCTGTGATCTAAGGAGGCTGGAGATGTATTGCGCACCCCTCAAGCCTGCCAAGTCAGCTCGCTCTGTCCGTGCCCAG | insulin like growth factor 1 |
| IGF1R | NM_000875.4 | insulin-like growth factor 1 receptor | TCTCCTGAGTCCCTCAAGGATGGAGTCTTCACCACTTACTCGGACGTCTGGTCCTTCGGGGTCGTCCTCTGGGAGATCGCCACACTGGCCGAGCAGCCCT | insulin like growth factor 1 receptor |
| IHH | NM_002181.2 | Indian hedgehog (Drosophila) homolog | GGCAGCCATCCCGGCCATTCTGAGGTATGACATTCCTCCCCGGCCACACTCCTCAAGACACATCCAGAGACTGTTGCTGTCTGTGGGCAGAGTTCTGTGT | Indian hedgehog signaling molecule |
| IKBKB | NM_001556.1 | inhibitor of kappa light polypeptide gene enhancer in B-cells, kinase beta | GTGATCTATACGCAGCTCAGTAAAACTGTGGTTTGCAAGCAGAAGGCGCTGGAACTGTTGCCCAAGGTGGAAGAGGTGGTGAGCTTAATGAATGAGGATG | inhibitor of nuclear factor kappa B kinase subunit beta |
| IL10 | NM_000572.2 |  | AAGGATCAGCTGGACAACTTGTTGTTAAAGGAGTCCTTGCTGGAGGACTTTAAGGGTTACCTGGGTTGCCAAGCCTTGTCTGAGATGATCCAGTTTTACC | interleukin 10 |
| IL10RA | NM_001558.2 | IL10R;interleukin 10 receptor, alpha | TGCCCAGCCCTCCGTCTGTGTGGTTTGAAGCAGAATTTTTCCACCACATCCTCCACTGGACACCCATCCCAAATCAGTCTGAAAGTACCTGCTATGAAGT | interleukin 10 receptor subunit alpha |
| IL11 | NM_000641.2 |  | TGAGACAGAGAACAGGGAATTAAATGTGTCATACATATCCACTTGAGGGCGATTTGTCTGAGAGCTGGGGCTGGATGCTTGGGTAACTGGGGCAGGGCAG | interleukin 11 |
| IL12RB2 | NM_001559.2 | interleukin 12 receptor, beta 2 | CCTCCGTGGGACATTAGAATCAAATTTCAAAAGGCTTCTGTGAGCAGATGTACCCTTTATTGGAGAGATGAGGGACTGGTACTGCTTAATCGACTCAGAT | interleukin 12 receptor subunit beta 2 |
| IL13 | NM_002188.2 |  | TTTCTTTCTGATGTCAAAAATGTCTTGGGTAGGCGGGAAGGAGGGTTAGGGAGGGGTAAAATTCCTTAGCTTAGACCTCAGCCTGTGCTGCCCGTCTTCA | interleukin 13 |
| IL17A | NM_002190.2 | CTLA8,IL17;interleukin 17 (cytotoxic T-lymphocyte-associated serine esterase 8) | TACTACAACCGATCCACCTCACCTTGGAATCTCCACCGCAATGAGGACCCTGAGAGATATCCCTCTGTGATCTGGGAGGCAAAGTGCCGCCACTTGGGCT | interleukin 17A |
| IL18R1 | NM_003855.3 |  | CAGTGGAATTAGGAAAAAACGTAAGGCTCAACTGCTCTGCTTTGCTGAATGAAGAGGATGTAATTTATTGGATGTTCGGGGAAGAAAATGGATCGGATCC | interleukin 18 receptor 1 |
| IL1B | NM_000576.2 |  | GGGACCAAAGGCGGCCAGGATATAACTGACTTCACCATGCAATTTGTGTCTTCCTAAAGAGAGCTGTACCCAGAGAGTCCTGTGCTGAATGTGGACTCAA | interleukin 1 beta |
| IL1R1 | NM_001320984.1 | IL1R,IL1RA;interleukin 1 receptor, type I | TCTACAGAACAAGCCTCCAGGATTCATCAACACAAAGAGAAACTTTGGTTTGTTCCTGCTAAGGTGGAGGATTCAGGACATTACTATTGCGTGGTAAGGA | interleukin 1 receptor type 1 |
| IL1RAP | NM_002182.2 |  | CCGCCTCCCCGAGAACCGCATTAGTAAGGAGAAAGATGTGCTGTGGTTCCGGCCCACTCTCCTCAATGACACTGGCAACTATACCTGCATGTTAAGGAAC | interleukin 1 receptor accessory protein |
| IL20RB | NM_144717.2 | FNDC6;fibronectin type III domain containing 6,interleukin 20 receptor beta,interleukin 20 receptor beta subunit | GGGAAACTGGTGACACTCTACAGTCTGACTGATTCAGTGTTTCTGGAGAGCAGGACATAAATGTATGATGAGAATGATCAAGGACTCTACACACTGGGTG | interleukin 20 receptor subunit beta |
| IL21R | NM_021798.2 |  | CGTGTTTGTGGTCAACAGATGACAACAGCCGTCCTCCCTCCTAGGGTCTTGTGTTGCAAGTTGGTCCACAGCATCTCCGGGGCTTTGTGGGATCAGGGCA | interleukin 21 receptor |
| IL27 | NM_145659.3 | IL30;interleukin 30 | CAGGAGCTGCGGAGGGAGTTCACAGTCAGCCTGCATCTCGCCAGGAAGCTGCTCTCCGAGGTTCGGGGCCAGGCCCACCGCTTTGCGGAATCTCACCTGC | interleukin 27 |
| IL2RA | NM_000417.1 | IL2R,IDDM10;insulin-dependent diabetes mellitus 10,interleukin 2 receptor, alpha | CTTGGTAAGAAGCCGGGAACAGACAACAGAAGTCATGAAGCCCAAGTGAAATCAAAGGTGCTAAATGGTCGCCCAGGAGACATCCGTTGTGCTTGCCTGC | interleukin 2 receptor subunit alpha |
| IL2RB | NM_000878.2 | IL15RB;interleukin 15 receptor, beta,interleukin 2 receptor, beta | GTCCTGCTGCCCGAGCCAGGAACTGTGTGTGTTGCAGGGGGGCAGTAACTCCCCAACTCCCTCGTTAATCACAGGATCCCACGAATTTAGGCTCAGAAGC | interleukin 2 receptor subunit beta |
| IL33 | NM_033439.2 | C9orf26;chromosome 9 open reading frame 26 (NF-HEV) | GCCAGAAATCTTCTAACCTACCAGAGCCTAGATGAGACACCGAATTAACATTAAAATTTCAGTAACTGACTGTCCCTCATGTCCATGGCCTACCATCCCT | interleukin 33 |
| IL4 | NM_000589.2 |  | GACACTCGCTGCCTGGGTGCGACTGCACAGCAGTTCCACAGGCACAAGCAGCTGATCCGATTCCTGAAACGGCTCGACAGGAACCTCTGGGGCCTGGCGG | interleukin 4 |
| IL6 | NM_000600.3 | IFNB2;interleukin 6 (interferon, beta 2) | GGCACTGGCAGAAAACAACCTGAACCTTCCAAAGATGGCTGAAAAAGATGGATGCTTCCAATCTGGATTCAATGAGGAGACTTGCCTGGTGAAAATCATC | interleukin 6 |
| IL6ST | NM_002184.2 | interleukin 6 signal transducer (gp130, oncostatin M receptor) | CAAAACACTTCGAGCACTGTCCAGTATTCTACCGTGGTACACAGTGGCTACAGACACCAAGTTCCGTCAGTCCAAGTCTTCTCAAGATCCGAGTCTACCC | interleukin 6 signal transducer |
| ILK | NM_004517.2 | integrin-linked kinase | TCACACACTGGATGCCGTATGGATCCCTCTACAATGTACTACATGAAGGCACCAATTTCGTCGTGGACCAGAGCCAGGCTGTGAAGTTTGCTTTGGACAT | integrin linked kinase |
| INPP5D | NM_005541.3 | inositol polyphosphate-5-phosphatase, 145kD,inositol polyphosphate-5-phosphatase, 145kDa | ATAATGGCCACATGGATCGAACACTCATGATGTGCCAAGTGCTGTGCTAAGTGCTTTACGAACATTCGTCATATCAGGATGACCTCGAGAGCTGAGGCTC | inositol polyphosphate-5-phosphatase D |
| INSR | NM_000208.2 |  | CACCCGGGGTTCTGTCCGCATCGAGAAGAACAATGAGCTCTGTTACTTGGCCACTATCGACTGGTCCCGTATCCTGGATTCCGTGGAGGATAATTACATC | insulin receptor |
| IQGAP1 | NM_003870.3 |  | GAAAAATCCGAATGCCATGCTTGTAAATCTTGAAGAGCCCTTGGCATCCACTTACCAGGATATACTTTACCAGGCTAAGCAGGACAAAATGACAAATGCT | IQ motif containing GTPase activating protein 1 |
| IRAK3 | NM_007199.1 | interleukin-1 receptor-associated kinase 3 | AGGTAAATATAGATCCTTCTTCAGAAGCTCCAGGGCATTCTTGCAGGAGCAGGCCAGTGGAGAGCAGCTGTTCCTCCAAATTTTCCTGGGATGAATATGA | interleukin 1 receptor associated kinase 3 |
| IRF1 | NM_002198.2 |  | TTAGTCGAGGCAAGACGTGCGCCCGAGCCCCGCCGAACCGAGGCCACCCGGAGCCGTGCCCAGTCCACGCCGGCCGTGCCCGGCGGCCTTAAGAACCCGG | interferon regulatory factor 1 |
| IRF3 | NM_001571.5 |  | TCATGGCCCCAGGACCAGCCGTGGACCAAGAGGCTCGTGATGGTCAAGGTTGTGCCCACGTGCCTCAGGGCCTTGGTAGAAATGGCCCGGGTAGGGGGTG | interferon regulatory factor 3 |
| IRF4 | NM_002460.1 | MUM1 | GGGCACTGTTTAAAGGAAAGTTCCGAGAAGGCATCGACAAGCCGGACCCTCCCACCTGGAAGACGCGCCTGCGGTGCGCTTTGAACAAGAGCAATGACTT | interferon regulatory factor 4 |
| IRF8 | NM_002163.2 | ICSBP1;interferon consensus sequence binding protein 1 | AGTTTAAAGAAGGGGACAAAGCTGAACCAGCCACTTGGAAGACGAGGTTACGCTGTGCTTTGAATAAGAGCCCAGATTTTGAGGAAGTGACGGACCGGTC | interferon regulatory factor 8 |
| IRS1 | NM_005544.2 |  | TTGATGGTGGCATCAAACTACCGATTTAAAACTGGAAGTTGCTGGTACTCAAACCAAAAGTTCATACTCTGGCGACACGAAGGGTTTCCTTTGAGCAACG | insulin receptor substrate 1 |
| ISG15 | NM_005101.3 | G1P2;interferon, alpha-inducible protein (clone IFI-15K) | CCCGGCAGCACGGTCCTGCTGGTGGTGGACAAATGCGACGAACCTCTGAGCATCCTGGTGAGGAATAACAAGGGCCGCAGCAGCACCTACGAGGTACGGC | ISG15 ubiquitin like modifier |
| ISG20 | NM_002201.5 | interferon stimulated exonuclease gene 20kDa | AGCCCGCCGAGGGCTGCCCCGCCTGGCTGTGTCAGACTGAAGCCCCATCCAGCCCGTTCCGCAGGGACTAGAGGCTTTCGGCTTTTTGGGACAGCAACTA | interferon stimulated exonuclease gene 20 |
| ITCH | NM_001257138.1 | itchy (mouse homolog) E3 ubiquitin protein ligase,itchy E3 ubiquitin protein ligase homolog (mouse) | GAGGTGACAAAGAGCCAACAGAGACAATAGGAGACTTGTCAATTTGTCTTGATGGGCTACAGTTAGAGTCTGAAGTTGTTACCAATGGTGAAACTACATG | itchy E3 ubiquitin protein ligase |
| ITGA1 | NM_181501.1 | integrin, alpha 1 | AAGTGGCAAGACTATAAGGAAAGAGTATGCACAACGTATTCCATCAGGTGGGGATGGTAAGACACTGAAATTTTTTGGCCAGTCTATCCACGGAGAAATG | integrin subunit alpha 1 |
| ITGA4 | NM_000885.4 | CD49D;integrin, alpha 4 (antigen CD49D, alpha 4 subunit of VLA-4 receptor) | GCCCACTGCCAACTGGCTCGCCAACGCTTCAGTGATCAATCCCGGGGCGATTTACAGATGCAGGATCGGAAAGAATCCCGGCCAGACGTGCGAACAGCTC | integrin subunit alpha 4 |
| ITGA5 | NM_002205.2 | FNRA;integrin, alpha 5 (fibronectin receptor, alpha polypeptide) | GCCAGCTGCACTGATGCTGCCCCTCATCTCTCTGCCCAACCCTTCCCTCACCTTGGCACCAGACACCCAGGACTTATTTAAACTCTGTTGCAAGTGCAAT | integrin subunit alpha 5 |
| ITGA9 | NM_002207.2 | integrin, alpha 9 | CATGTCTCCAACCTCCTTTGTATATGGCGAGTCCGTGGACGCAGCCAACTTCATTCAGCTGGATGACCTGGAGTGTCACTTTCAGCCCATCAATATCACC | integrin subunit alpha 9 |
| ITGB1 | NM_002211.3 | FNRB,MSK12,MDF2;integrin, beta 1 (fibronectin receptor, beta polypeptide, antigen CD29 includes MDF2, MSK12) | TGGGTGGTGCACAAATTCAACATTTTTACAGGAAGGAATGCCTACTTCTGCACGATGTGATGATTTAGAAGCCTTAAAAAAGAAGGGTTGCCCTCCAGAT | integrin subunit beta 1 |
| ITGB3 | NM_000212.2 | GP3A;integrin, beta 3 (platelet glycoprotein IIIa, antigen CD61) | GAATAAGCCTTGGAATTAGATATGGGGCAATGACTGAGCCCTGTCTCACCCATGGATTACTCCTTACTGTAGGGAATGGCAGTATGGTAGAGGGATAAAT | integrin subunit beta 3 |
| ITPR2 | NM_002223.2 | inositol 1,4,5-triphosphate receptor, type 2,inositol 1,4,5-trisphosphate receptor, type 2 | CCCATAGACACAGATGAAGAGAGGCCTGTTATGTTAAAGATTGGAACCTGCCAAACCAAAGAAGATAAAGAAGCGTTCGCAATCGTGTCTGTTCCACTGT | inositol 1,4,5-trisphosphate receptor type 2 |
| JAG1 | NM_000214.2 | AGS,JAGL1;Alagille syndrome,jagged 1 | TTGCTTGTGGAGGCGTGGGATTCCAGTAATGACACCGTTCAACCTGACAGTATTATTGAAAAGGCTTCTCACTCGGGCATGATCAACCCCAGCCGGCAGT | jagged canonical Notch ligand 1 |
| JAG2 | NM_145159.1 | jagged 2 | ATTTTTGTAAAGTTTCCGTGCGTGGCACTCGCTGTATGAAAGGAGAGAGCAAAGGGTGTCTGCGTCGTCACCAAATCGTAGCGTTTGTTACCAGAGGTTG | jagged canonical Notch ligand 2 |
| JAK1 | NM_002227.1 | JAK1B | GAGAACACCAAGCTCTGGTATGCTCCAAATCGCACCATCACCGTTGATGACAAGATGTCCCTCCGGCTCCACTACCGGATGAGGTTCTATTTCACCAATT | Janus kinase 1 |
| JAK2 | NM_004972.3 |  | TTGATGTCAGTATTAAGCAAGCAAACCAAGAGGGTTCAAATGAAAGCCGAGTTGTAACTATCCATAAGCAAGATGGTAAAAATCTGGAAATTGAACTTAG | Janus kinase 2 |
| JMJD4 | NM_001161465.1 |  | ATGACTCTCAGAGACTACATCACCTACTGGAAAGAGTACATACAGGCGGGCTACTCCTCTCCCAGGGGCTGTCTCTACCTCAAAGACTGGCACTTGTGCA | jumonji domain containing 4 |
| JUN | NM_002228.3 | v-jun avian sarcoma virus 17 oncogene homolog,jun oncogene | ACACAGCCAGCCAGCCAGGTCGGCAGTATAGTCCGAACTGCAAATCTTATTTTCTTTTCACCTTCTCTCTAACTGCCCAGAGCTAGCGCCTGTGGCTCCC | Jun proto-oncogene, AP-1 transcription factor subunit |
| KANSL1 | NM_001193465.1 | KIAA1267;KIAA1267 | GGTCATATTTCAGAGTCACTGTCTACCAAATCATGTGGAGCACTCAGACCTGTCAATGGAGTTATTAACACTCTTCAGCCTGTCTTGGCAGACCACATTC | KAT8 regulatory NSL complex subunit 1 |
| KAT6A | NM_001099412.1 | ZNF220,RUNXBP2,MYST3;runt-related transcription factor binding protein 2,MYST histone acetyltransferase (monocytic leukemia) 3,K(lysine) acetyltransferase 6A | AAACTCGCAAACCCGCTTTATACTGAGTGGATTTTGGAGGCCATCAAAAAAGTGAAAAAGCAGAAACAGCGTCCTTCAGAAGAAAGGATATGCAATGCTG | lysine acetyltransferase 6A |
| KDM2A | NM_012308.1 | FBXL11;F-box and leucine-rich repeat protein 11,lysine (K)-specific demethylase 2A | TCTCACAGAGCTCAATATGGCAGGTTGCAATAAATTGACAGACCAGACCCTGATCTACCTACGGCGCATTGCCAACGTCACCTTGATCGACCTTCGAGGA | lysine demethylase 2A |
| KDM3B | NM_016604.3 | C5orf7,JMJD1B;chromosome 5 open reading frame 7,jumonji domain containing 1B,lysine (K)-specific demethylase 3B | AAGAGCAAGGCCAGCCTACCCAACTTTCTTGACCACATCATTGCCTCAGTGGTAGAAAATAAGAAAACCTCAGATGCTTCAAAGCGGGCCTGCAACTTGA | lysine demethylase 3B |
| KDM5D | NM_001146705.1 | HYA,HY,SMCY,JARID1D;Jumonji, AT rich interactive domain 1D (RBP2-like),Smcy homolog, Y-linked (mouse),jumonji, AT rich interactive domain 1D,lysine (K)-specific demethylase 5D | AAATATGTTCCTGACACCTTCCACAGACCACAGCCCTTTCTTGAAAGGAAACCAAAATAGCTTACAACACAAGGATTCAGGCTCTTCAGCTGCTTGTCCT | lysine demethylase 5D |
| KDR | NM_002253.2 | kinase insert domain receptor (a type III receptor tyrosine kinase) | CAATCACACAATTAAAGCGGGGCATGTACTGACGATTATGGAAGTGAGTGAAAGAGACACAGGAAATTACACTGTCATCCTTACCAATCCCATTTCAAAG | kinase insert domain receptor |
| KIF3A | NM_007054.5 |  | GTTGGTGCAACTAATATGAACGAACATAGTTCCCGTTCCCATGCCATCTTTACAATTACTATAGAATGCAGTGAAAAAGGCATTGATGGTAACATGCATG | kinesin family member 3A |
| KIR2DL3 | NM_015868.2 | killer cell immunoglobulin-like receptor, two domains, long cytoplasmic tail, 3 | CCTTCTGCACAGAGAAGGGAAGTTTAAGGACACTTTGCACCTCATTGGAGAGCACCATGATGGGGTCTCCAAGGCCAACTTCTCCATCGGTCCCATGATG | killer cell immunoglobulin like receptor, two Ig domains and long cytoplasmic tail 3 |
| KIR3DL1/2 | NM_001322168.1 | killer cell immunoglobulin-like receptor, three domains, long cytoplasmic tail, 2 | AATCAGGAGAGAGAGTCATCCTGCAATGTTGGTCAGATATCATGTTTGAGCACTTCTTTCTGCACAAAGAGGGGATCTCTAAGGACCCCTCACGCCTCGT | killer cell immunoglobulin like receptor, three Ig domains and long cytoplasmic tail 2 |
| KLF5 | NM_001730.3 | BTEB2;Kruppel-like factor 5 (intestinal) | AACGTCTTCCTCCCTGACATCACTCACCTGAGAACTGGCCTCTACAAATCCCAGAGACCGTGCGTAACACACATCAAGACAGAACCTGTTGCCATTTTCA | Kruppel like factor 5 |
| KLKB1 | NM_000892.3 | KLK3;kallikrein B, plasma (Fletcher factor) 1 | ATAGTGTTACAGGAACCCTGCCAAAAGTACATCGAACAGGTGCAGTTTCTGGACATTCCTTGAAGCAATGTGGTCATCAAATAAGTGCTTGCCATCGAGA | kallikrein B1 |
| KLRB1 | NM_002258.2 | NKR;killer cell lectin-like receptor subfamily B, member 1 | TGAGTTAAACTTACCCACAGACTCAGGCCCAGAAAGTTCTTCACCTTCATCTCTTCCTCGGGATGTCTGTCAGGGTTCACCTTGGCATCAATTTGCCCTG | killer cell lectin like receptor B1 |
| KLRD1 | NM_007334.2 | CD94;killer cell lectin-like receptor subfamily D, member 1 | TGGTCTTGAACTCCTGGCCTCAAGGGATTCTCCCACCTTGGATTCCCAAAGTGCTGGGATTATAGGTGTGAACCACCATCCCTGGCCCTCTTCACATTCT | killer cell lectin like receptor D1 |
| KLRK1 | NM_007360.3 | D12S2489E;DNA segment on chromosome 12 (unique) 2489 expressed sequence,killer cell lectin-like receptor subfamily K, member 1 | ATAGAAGGCTTTTATCCACAAGAATCAAGATCTTCCCTCTCTGAGCAGGAATCCTTTGTGCATTGAAGACTTTAGATTCCTCTCTGCGGTAGACGTGCAC | killer cell lectin like receptor K1 |
| KMT2D | NM_003482.3 | TNRC21,MLL2;trinucleotide repeat containing 21,myeloid/lymphoid or mixed-lineage leukemia 2,lysine (K)-specific methyltransferase 2D | CCAACATTAATTTTCCTAATCTCAAGCAAGACTACCCAGACTGGTCAAGCCGTTGCAAACAAATCATGAAGCTCTGGAGAAAGGTTCCAGCAGCTGACAA | lysine methyltransferase 2D |
| KNG1 | NM_000893.2 | KNG,BDK;kininogen | TATTGTACCGCATAACTGAAGCCACTAAGACGGTTGGCTCTGACACGTTTTATTCCTTCAAGTACGAAATCAAGGAGGGGGATTGTCCTGTTCAAAGTGG | kininogen 1 |
| KRAS | NM_033360.2 | KRAS2;v-Ki-ras2 Kirsten rat sarcoma 2 viral oncogene homolog,v-Ki-ras2 Kirsten rat sarcoma viral oncogene homolog,Kirsten rat sarcoma viral oncogene homolog | GGACGAATATGATCCAACAATAGAGGATTCCTACAGGAAGCAAGTAGTAATTGATGGAGAAACCTGTCTCTTGGATATTCTCGACACAGCAGGTCAAGAG | KRAS proto-oncogene, GTPase |
| LAG3 | NM_002286.5 | lymphocyte-activation gene 3 | CTTTTGGTGACTGGAGCCTTTGGCTTTCACCTTTGGAGAAGACAGTGGCGACCAAGACGATTTTCTGCCTTAGAGCAAGGGATTCACCCTCCGCAGGCTC | lymphocyte activating 3 |
| LAMA3 | NM_000227.3 | LAMNA;laminin, alpha 3 (nicein (150kD), kalinin (165kD), BM600 (150kD), epilegrin),laminin, alpha 3 | CAGAGGACTGGTGTTTCACACGGGCACTAAGAACTCCTTTATGGCTCTTTATCTTTCAAAAGGACGTCTGGTCTTTGCACTGGGGACAGATGGGAAAAAA | laminin subunit alpha 3 |
| LAMC1 | NM_002293.3 | LAMB2;laminin, gamma 1 (formerly LAMB2) | TCTTGATAGGAAAGTGTCTGACCTGGAGAATGAAGCCAAGAAGCAGGAGGCTGCCATCATGGACTATAACCGAGATATCGAGGAGATCATGAAGGACATT | laminin subunit gamma 1 |
| LAMTOR2 | NM_001145264.1 | ROBLD3;roadblock domain containing 3 | TACGGGGACACTGACGCCCGGGTCACCGCTGCCATAGCCAGTAACATCTGGGCCGCCTACGACCGGAACGGGAACCAAGCGTTTAATGAAGACAATCTCA | late endosomal/lysosomal adaptor, MAPK and MTOR activator 2 |
| LAMTOR4 | NM_001008395.2 | C7orf59;chromosome 7 open reading frame 59 | CTGGACGGGCCCAGGCTTGGGGACTCTGAGCTGTGTTAAGGAGAACAAGGGCAAGGAGACCTCCCTTTGTGCTCCCTCACTCCCTAATAAACATGAGTCT | late endosomal/lysosomal adaptor, MAPK and MTOR activator 4 |
| LATS1 | NM_004690.2 | LATS (large tumor suppressor, Drosophila) homolog 1,LATS, large tumor suppressor, homolog 1 (Drosophila) | TAAAGAATCCTTAGTTCCTCAGAGGCATGGCCCGCCACTAGGAGAAAGTGTGGCCTATCATTCTGAGAGTCCCAACTCACAGACAGATGTAGGAAGACCT | large tumor suppressor kinase 1 |
| LATS2 | NM_014572.2 | LATS (large tumor suppressor, Drosophila) homolog 2,LATS, large tumor suppressor, homolog 2 (Drosophila) | TGCATATCTAGTATAATAGGAAGTGTGAGCAAGGTGATGATGTGGCTGTGATTTCCGACGTCTGGTGTGTGGAGAGTACTGCATGAGCAGAGTTCTTCTA | large tumor suppressor kinase 2 |
| LDLRAP1 | NM_015627.2 |  | CTCACCAACCAGCTCATTGAGAACGTGTCCATATACAGGATCTCCTATTGCACAGCAGACAAGATGCACGACAAGGTGTTTGCATACATCGCCCAGAGCC | low density lipoprotein receptor adaptor protein 1 |
| LEP | NM_000230.2 | OBS,OB;leptin (murine obesity homolog),leptin (obesity homolog, mouse) | GTAGTTCTTGTCTGATTGGCTCACCCAAGCAAGGCCAAAATTACCAAAAATCTTGGGGGGTTTTTACTCCAGTGGTGAAGAAAACTCCTTTAGCAGGTGG | leptin |
| LEPR | NM_001003679.1 |  | TTCCAGACTTGTGTGCAGTCTATGCTGTTCAGGTGCGCTGTAAGAGGCTAGATGGACTGGGATATTGGAGTAATTGGAGCAATCCAGCCTACACAGTTGT | leptin receptor |
| LGALS3 | NM_001177388.1 | LGALS2;lectin, galactoside-binding, soluble, 3 | CACGGTGAAGCCCAATGCAAACAGAATTGCTTTAGATTTCCAAAGAGGGAATGATGTTGCCTTCCACTTTAACCCACGCTTCAATGAGAACAACAGGAGA | galectin 3 |
| LGMN | NM_001008530.2 | PRSC1;protease, cysteine, 1 (legumain) | GATCATTCACCGCAATGGGATTCCTGACGAACAGATCGTTGTGATGATGTACGATGACATTGCTTACTCTGAAGACAATCCCACTCCAGGAATTGTGATC | legumain |
| LILRB2 | NM_001278405.1 | leukocyte immunoglobulin-like receptor, subfamily B (with TM and ITIM domains), member 2 | GATACGACCAGAGCTTGTGAAGAACGGCCAGTTCCACATCCCATCCATCACCTGGGAACACACAGGGCGATATGGCTGTCAGTATTACAGCCGCGCTCGG | leukocyte immunoglobulin like receptor B2 |
| LIPA | NM_000235.3 | lipase A, lysosomal acid, cholesterol esterase | AAGCCTTTGACTGGGGAAGCAGTGCCAAGAATTATTTTCATTACAACCAGAGTTATCCTCCCACATACAATGTGAAGGACATGCTTGTGCCGACTGCAGT | lipase A, lysosomal acid type |
| LIPC | NM_000236.2 | lipase, hepatic | TCGAATCAATCATCCGGACACGTTACAGGAGTGCGGCTTCAACTCCTCCCTGCCTCTGGTGATGATAATCCACGGGTGGTCGGTGGACGGCGTGCTAGAA | lipase C, hepatic type |
| LIPG | NM_006033.2 | lipase, endothelial | GGAAACTGACATTTTGTACAGAAGACCCTGAGAACACCAGCATATCCCCAGGCCGGGAGCTCTGGTTTCGCAAGTGTCGGGATGGCTGGAGGATGAAAAA | lipase G, endothelial type |
| LOX | NM_002317.4 |  | CGCTACACAGGACATCATGCGTATGCCTCAGGCTGCACAATTTCACCGTATTAGAAGGCAAAGCAAAACTCCCAATGGATAAATCAGTGCCTGGTGTTCT | lysyl oxidase |
| LOXL1 | NM_005576.2 | lysyl oxidase-like 1 | ACCAACAACGTGGTGAGATGCAACATTCACTACACAGGTCGCTACGTTTCTGCAACAAACTGCAAAATTGTCCAATCCTGATCTCCGGGAGGGACAGATG | lysyl oxidase like 1 |
| LOXL2 | NM_002318.2 | lysyl oxidase-like 2 | GGTTTGCCATCCTCCTCTAGTTAAAAGTAAGGGGGAAAAGAGTAAACGCGCGACTCCAGCGCGCGGCTACCTACGCTTGGTGCTTGCTTTCTCCAGCCAT | lysyl oxidase like 2 |
| LOXL4 | NM_032211.6 | lysyl oxidase-like 4 | GTCAGTTTAGTTAAGGATGGAACCTGGGAAAGGCCACCATTCCTGCTTGATGGGGCTCTGATTTGCTCTTGCTCAAGTGGAATAAAACCCCATGGTCTTC | lysyl oxidase like 4 |
| LPA | NM_005577.2 | LP;lipoprotein, Lp(a) | AATGTCCAGTGATGGAATCAACTCTCCTCACAACTCCCACGGTGGTCCCAGTTCCAAGCACAGAGCTTCCTTCTGAAGAAGCACCAACTGAAAACAGCAC | lipoprotein(a) |
| LPAR5 | NM_020400.4 | GPR93,GPR92;G protein-coupled receptor 92 | TCACCACTGAGGCCTAATACAAGAGTTCCTATGGACAGAACTACATTCTTTCTCGCATAGTGACTTGTGACAATTTAGACTTGGCATCCAGCATGGGATA | lysophosphatidic acid receptor 5 |
| LPCAT1 | NM_024830.3 | AYTL2;acyltransferase like 2 | GTCTTCAGCCCGAGTTCCAGGGGCCTCGGGGGCTGTTTGTATCTTGTTCCTTTGTGAAGTGTGTTGCAGAACCGACGCTTACTGTGCGAGAATCGGAGGG | lysophosphatidylcholine acyltransferase 1 |
| LPL | NM_000237.2 | LIPD | TTTTCCGCGGCACGAATCAGACTCATCTACACAGCAGTATGAATGATGTTTTAGAATGATTCCCTCTTGCTATTGGAATGTGGTCCAGACGTCAACCAGG | lipoprotein lipase |
| LRP1 | NM_002332.2 | APR,A2MR,IGFBP3R1,IGFBP-3R;alpha-2-macroglobulin receptor,low density lipoprotein receptor-related protein 1 | GAGAGCTGCCGCAGCCTGGACCCCTTCAAGCCGTTCATCATTTTCTCCAACCGCCATGAAATCCGGCGCATCGATCTTCACAAAGGAGACTACAGCGTCC | LDL receptor related protein 1 |
| LRP2 | NM_004525.2 | low density lipoprotein receptor-related protein 2 | TCAGTGTTGTGTATTACACTGTGCGAGGGGAGGGCTCTAGGTTTGGTGCTATCAAACGTGCCTACATCCCCAACTTTGAATCCGGCCGCAATAATCTTGT | LDL receptor related protein 2 |
| LRP6 | NM_002336.1 | low density lipoprotein receptor-related protein 6 | CTTAGATTATCCAGAAGGCATGGCAGTAGACTGGCTTGGGAAGAACTTGTACTGGGCAGACACAGGAACGAATCGAATTGAGGTGTCAAAGTTGGATGGG | LDL receptor related protein 6 |
| LRRC32 | NM_001128922.1 | D11S833E,GARP;glycoprotein A repetitions predominant | CGACAGCTTTCTTGAGCACCTGACCTCCCTGTGCTTCCTGAACCTCAGCAGAAACTGCTTGCGGACCTTTGAGGCCCGGCGCTTAGGCTCCCTGCCCTGC | leucine rich repeat containing 32 |
| LTA | NM_000595.2 | TNFB;lymphotoxin alpha (TNF superfamily, member 1) | CTGATCAAGTCACCGGAGCTTTCAAAGAAGGAATTCTAGGCATCCCAGGGGACCACACCTCCCTGAACCATCCCTGATGTCTGTCTGGCTGAGGATTTCA | lymphotoxin alpha |
| LTB | NM_002341.1 | TNFC | AGGAACAGGCGTTTCTGACGAGCGGGACGCAGTTCTCGGACGCCGAGGGGCTGGCGCTCCCGCAGGACGGCCTCTATTACCTCTACTGTCTCGTCGGCTA | lymphotoxin beta |
| LYN | NM_002350.3 | v-yes-1 Yamaguchi sarcoma viral related oncogene homolog | CCACGAATCACTTTTCCCTGTATCAGCGACATGATTAAACATTACCAAAAGCAGGCAGATGGCTTGTGCAGAAGATTGGAGAAGGCTTGTATTAGTCCCA | LYN proto-oncogene, Src family tyrosine kinase |
| MAF | NM_005360.4 | v-maf avian musculoaponeurotic fibrosarcoma oncogene homolog | TAATGACTTCGATCTGATGAAGTTTGAAGTGAAAAAGGAACCGGTGGAGACCGACCGCATCATCAGCCAGTGCGGCCGTCTCATCGCCGGGGGCTCGCTG | MAF bZIP transcription factor |
| MAML1 | NM_014757.4 | mastermind (drosophila)-like 1,mastermind-like 1 (Drosophila) | ATGGCTTATCTTCCCCAGCAGCTGTCCCATATAAGTCACGAGCAGAACTCCCTGTTTCTGATGAAGCCAAAGCCAGGAAATATGCCTTTCCGATCACTGG | mastermind like transcriptional coactivator 1 |
| MAML2 | NM_032427.2 | mastermind (Drosophila)-like 2,mastermind-like 2 (Drosophila) | TATCAAAAGAATTCGTGTTGGGGAGAATCTCTCTGCAGGACAAGGTGGCCTCCAAATAAACAATGGACAAAGTCAGATTATGTCAGGGACCTTGCCTATG | mastermind like transcriptional coactivator 2 |
| MAML3 | NM_018717.4 | TNRC3;trinucleotide repeat containing 3,mastermind-like 3 (Drosophila) | TGGAAGCCATCAACAATTTGCCCAGTAACATGCCACTGCCTTCAGCTTCTCCTCTTCACCAACTTGACCTGAAACCTTCTTTGCCCTTGCAGAACAGTGG | mastermind like transcriptional coactivator 3 |
| MAP1LC3A | NM_032514.2 |  | ACCAGCACAGCATGGTGAGTGTGTCCACGCCCATCGCGGACATCTACGAGCAGGAGAAAGACGAGGACGGCTTCCTCTATATGGTCTACGCCTCCCAGGA | microtubule associated protein 1 light chain 3 alpha |
| MAP2K1 | NM_002755.2 | PRKMK1 | ACGGAATGGACAGCCGACCTCCCATGGCAATTTTTGAGTTGTTGGATTACATAGTCAACGAGCCTCCTCCAAAACTGCCCAGTGGAGTGTTCAGTCTGGA | mitogen-activated protein kinase kinase 1 |
| MAP3K1 | NM_005921.1 | MEKK1;MEKK1 | ACTTCAGAGACTTCTCCAGCCAGTTGTAGACACCATCCTAGTCAAATGTGCAGATGCCAATAGCCGCACAAGTCAGCTGTCCATATCAACACTGTTGGAA | mitogen-activated protein kinase kinase kinase 1 |
| MAPK1 | NM_138957.2 | PRKM2,PRKM1 | ACTGCCAGAGAACCCTGAGGGAGATAAAAATCTTACTGCGCTTCAGACATGAGAACATCATTGGAATCAATGACATTATTCGAGCACCAACCATCGAGCA | mitogen-activated protein kinase 1 |
| MAPK10 | NM_138981.2 | PRKM10 | ATGGATGCCAACTTATGTCAAGTGATTCAGATGGAATTAGACCATGAGCGAATGTCTTACCTGCTGTACCAAATGTTGTGTGGCATTAAGCACCTCCATT | mitogen-activated protein kinase 10 |
| MAPK11 | NM_002751.6 | PRKM11 | CACCCTGATGGGCGCCGACCTGAACAACATCGTCAAGTGCCAGGCGCTGAGCGACGAGCACGTTCAATTCCTGGTTTACCAGCTGCTGCGCGGGCTGAAG | mitogen-activated protein kinase 11 |
| MAPK8 | NM_002750.2 | PRKM8 | TCTCTGTAGATGAAGCTCTCCAACACCCGTACATCAATGTCTGGTATGATCCTTCTGAAGCAGAAGCTCCACCACCAAAGATCCCTGACAAGCAGTTAGA | mitogen-activated protein kinase 8 |
| MAPK9 | NM_001135044.1 | PRKM9 | ATCATGAGCGACAGTAAATGTGACAGTCAGTTTTATAGTGTGCAAGTGGCAGACTCAACCTTCACTGTCCTAAAACGTTACCAGCAGCTGAAACCAATTG | mitogen-activated protein kinase 9 |
| MARCO | NM_006770.3 |  | CTCCAAGTGGTTCCTCTTGAGGGGAGCATTTCTGCTGGCTCCAGGACTTTGGCCATCTATAAAGCTTGGCAATGAGAAATAAGAAAATTCTCAAGGAGGA | macrophage receptor with collagenous structure |
| MASP1 | NM_001031849.2 | CRARF,PRSS5;mannan-binding lectin serine protease 1 (C4/C2 activating component of Ra-reactive factor),mannan-binding lectin serine peptidase 1 (C4/C2 activating component of Ra-reactive factor) | TATTTCTTCAAAGACCAAGTGCTCGTCAGCTGTGACACAGGCTACAAAGTGCTGAAGGATAATGTGGAGATGGACACATTCCAGATTGAGTGTCTGAAGG | mannan binding lectin serine peptidase 1 |
| MBTPS2 | NM_015884.3 | KFSD;membrane-bound transcription factor protease, site 2,keratosis follicularis spinulosa decalvans | TGGGTGGCAGTGTACTTTTGGCTGCCAATGTGACCCTGGGACTCTGGATGGTTACAGCACGGTAATGTTTGCACTCATCTGACAGAATCCCTGAGTTACA | membrane bound transcription factor peptidase, site 2 |
| MECP2 | NM_001110792.1 | RTT,MRX16,MRX79;mental retardation, X-linked 16,mental retardation, X-linked 79,Rett syndrome,methyl CpG binding protein 2 (Rett syndrome),methyl CpG binding protein 2 | CGCAGAAAAGTACAAACACCGAGGGGAGGGAGAGCGCAAAGACATTGTTTCATCCTCCATGCCAAGGCCAAACAGAGAGGAGCCTGTGGACAGCCGGACG | methyl-CpG binding protein 2 |
| MEF2A | NM_005587.2 |  | CAGTGACCAGCCCCAATGCTTTGTCCTACACTAACCCAGGGAGTTCACTGGTGTCCCCATCTTTGGCAGCCAGCTCAACGTTAACAGATTCAAGCATGCT | myocyte enhancer factor 2A |
| MEFV | NM_000243.2 | MEF;Mediterranean fever,MEFV, pyrin innate immunity regulator | TGTAAGCGCCACCTGAAGCAGGTCCAGCTGCTCTTCTGTGAGGATCACGATGAGCCCATCTGCCTCATCTGCAGTCTGAGTCAGGAGCACCAAGGCCACC | MEFV innate immuity regulator, pyrin |
| MET | NM_001127500.1 | met proto-oncogene | GGCTGACCATATGTGGCTGGGACTTTGGATTTCGGAGGAATAATAAATTTGATTTAAAGAAAACTAGAGTTCTCCTTGGAAATGAGAGCTGCACCTTGAC | MET proto-oncogene, receptor tyrosine kinase |
| MFAP3 | NM_001242336.1 | microfibrillar associated protein 3 | AGAACTGGCAAGAAGTGTCCCTCTTCCACCTCTTATTCTAAACTGTCGAGCCTTTGTTGAGGAGATGTTTGAGGCTGTGCGAGTGGACGACCCTGATGAC | microfibril associated protein 3 |
| MKI67 | NM_002417.2 | antigen identified by monoclonal antibody Ki-67 | AGCAGATGTAGAGGGAGAACTCTTAGCGTGCAGGAATCTAATGCCATCAGCAGGCAAAGCCATGCACACGCCTAAACCATCAGTAGGTGAAGAGAAAGAC | marker of proliferation Ki-67 |
| MLXIPL | NM_032951.2 | WBSCR14;Williams Beuren syndrome chromosome region 14,MLX interacting protein-like | GCCACTGTGCTCCAGCACCGTGACCTTGGGTGACTCGTCCGCTGTCTTTGGACCGCTGTGTTTCAATCTGCAAAATGGGGATGGGGAAGGTTCAATCAGC | MLX interacting protein like |
| MMP1 | NM_002421.3 | CLG;matrix metalloproteinase 1 (interstitial collagenase) | AAATGGGCTTGAAGCTGCTTACGAATTTGCCGACAGAGATGAAGTCCGGTTTTTCAAAGGGAATAAGTACTGGGCTGTTCAGGGACAGAATGTGCTACAC | matrix metallopeptidase 1 |
| MMP10 | NM_002425.1 | STMY2;matrix metalloproteinase 10 (stromelysin 2),stromelysin 2 | GCAGCAAAAGAGGAGGACTCCAACAAGGATCTTGCCCAGCAATACCTAGAAAAGTACTACAACCTCGAAAAGGATGTGAAACAGTTTAGAAGAAAGGACA | matrix metallopeptidase 10 |
| MMP11 | NM_005940.3 | STMY3;matrix metalloproteinase 11 (stromelysin 3),stromelysin 3 | AGCAGCCAAGGCCCTGATGTCCGCCTTCTACACCTTTCGCTACCCACTGAGTCTCAGCCCAGATGACTGCAGGGGCGTTCAACACCTATATGGCCAGCCC | matrix metallopeptidase 11 |
| MMP12 | NM_002426.3 | matrix metalloproteinase 12 (macrophage elastase) | ATGTTGACATCAACACATTTCGCCTCTCTGCTGATGACATACGTGGCATTCAGTCCCTGTATGGAGACCCAAAAGAGAACCAACGCTTGCCAAATCCTGA | matrix metallopeptidase 12 |
| MMP13 | NM_002427.2 | matrix metalloproteinase 13 (collagenase 3) | CTGGCGCCTGCATCCTCAGCAGGTTGATGCGGAGCTGTTTTTAACGAAATCATTTTGGCCAGAACTTCCCAACCGTATTGATGCTGCATATGAGCACCCT | matrix metallopeptidase 13 |
| MMP14 | NM_004995.2 | matrix metalloproteinase 14 (membrane-inserted),matrix metallopeptidase 14 (membrane-inserted) | GACAAGATTGATGCTGCTCTCTTCTGGATGCCCAATGGAAAGACCTACTTCTTCCGTGGAAACAAGTACTACCGTTTCAACGAAGAGCTCAGGGCAGTGG | matrix metallopeptidase 14 |
| MMP16 | NM_005941.4 | C8orf57;matrix metalloproteinase 16 (membrane-inserted),chromosome 8 open reading frame 57,matrix metallopeptidase 16 (membrane-inserted) | TTGACATGGCAGTCAGAGCAGGTGAATACAGATGCTCTTTCTGATTGCAAAGGTTATAGAGCTAAATTAGACAGCATTATGTGATACTAGCAAAGACAAC | matrix metallopeptidase 16 |
| MMP2 | NM_004530.2 | CLG4,CLG4A;matrix metalloproteinase 2 (gelatinase A, 72kDa gelatinase, 72kDa type IV collagenase) | CCCGGAGGGGCCTGGCAGCCGTGCCTTCAGCTCTACAGCTAATCAGCATTCTCACTCCTACCTGGTAATTTAAGATTCCAGAGAGTGGCTCCTCCCGGTG | matrix metallopeptidase 2 |
| MMP3 | NM_002422.3 | STMY1,STMY;matrix metalloproteinase 3 (stromelysin 1, progelatinase),stromelysin 1 | AGGCATAGAGACAACATAGAGCTAAGTAAAGCCAGTGGAAATGAAGAGTCTTCCAATCCTACTGTTGCTGTGCGTGGCAGTTTGCTCAGCCTATCCATTG | matrix metallopeptidase 3 |
| MMP7 | NM_002423.3 | MPSL1;matrix metalloproteinase 7 (matrilysin, uterine) | GTGCCAGATGTTGCAGAATACTCACTATTTCCAAATAGCCCAAAATGGACTTCCAAAGTGGTCACCTACAGGATCGTATCATATACTCGAGACTTACCGC | matrix metallopeptidase 7 |
| MMP8 | NM_002424.2 | CLG1;matrix metalloproteinase 8 (neutrophil collagenase) | CTCTCTCCTAGAGTCCAAACCCAAATGGGCCAGTTGGATCTGATGTTCGTCAGTTCTTTACTTCTATTTCCTGGGGTACTCAGGAGGGCACACACTATAG | matrix metallopeptidase 8 |
| MMP9 | NM_004994.2 | CLG4B;matrix metalloproteinase 9 (gelatinase B, 92kDa gelatinase, 92kDa type IV collagenase) | CACTACTGTGCCTTTGAGTCCGGTGGACGATGCCTGCAACGTGAACATCTTCGACGCCATCGCGGAGATTGGGAACCAGCTGTATTTGTTCAAGGATGGG | matrix metallopeptidase 9 |
| MMRN1 | NM_007351.2 | MMRN;multimerin | GCAGACATCCTTTTACTGGTGACAACTGCACTATCAAGCTTGTGGAAGAAAATGCTTTAGCTCCAGATTTTTCCAAAGGATCTTACAGATATGCACCCAT | multimerin 1 |
| MMUT | NM_000255.2 | MUT;methylmalonyl Coenzyme A mutase | GGTGAGTGGAGCATATCGCCAGGAATTTGGAGAAAGTAAAGAGATAACATCTGCTATCAAGAGGGTTCATAAATTCATGGAACGTGAAGGTCGCAGACCT | methylmalonyl-CoA mutase |
| MOB1B | NM_173468.3 | MOBKL1A;MOB1, Mps One Binder kinase activator-like 1A (yeast),MOB1 Mps One Binder homolog B (yeast) | AGAGGGTTCTCACCAGTATGAGCTCTTAAAACACGCAGAAGCCACACTTGGCAGTGGCAACCTTCGGATGGCTGTCATGCTTCCTGAAGGGGAAGATCTC | MOB kinase activator 1B |
| MS4A1 | NM_152866.2 | CD20;membrane-spanning 4-domains, subfamily A, member 1 | CTTCTGATGATCCCAGCAGGGATCTATGCACCCATCTGTGTGACTGTGTGGTACCCTCTCTGGGGAGGCATTATGTATATTATTTCCGGATCACTCCTGG | membrane spanning 4-domains A1 |
| MS4A2 | NM_000139.3 | FCER1B,IGER,APY;IgE responsiveness (atopic),membrane-spanning 4-domains, subfamily A, member 2 (Fc fragment of IgE, high affinity I, receptor for; beta polypeptide),membrane-spanning 4-domains, subfamily A, member 2 | GCAAATCTTGCTCTCCCACAGGAGCCTTCCAGTGTGCCTGCATTTGAAGTCTTGGAAATATCTCCCCAGGAAGTATCTTCAGGCAGACTATTGAAGTCGG | membrane spanning 4-domains A2 |
| MS4A4A | NM_024021.2 | MS4A4;membrane-spanning 4-domains, subfamily A, member 4,membrane-spanning 4-domains, subfamily A, member 4A | GTTTGAGGCCACCAAAAGATCAACAGACAAATGCTCCAGAAATCTATGCTGACTGTGACACAAGAGCCTCACATGAGAAATTACCAGTATCCAACTTCGA | membrane spanning 4-domains A4A |
| MTMR4 | NM_004687.4 |  | CCTGTTTTCAAGGGGAGTAAGATTGGTAACATTTGGGGAGACTGTATCTGTCTACTTAGCGTGGCTGTTTTGAGGGACTGTCCCATCAGTGAACAAACTG | myotubularin related protein 4 |
| MTOR | NM_004958.3 | FRAP,FRAP2,FRAP1;FK506 binding protein 12-rapamycin associated protein 1,mechanistic target of rapamycin (serine/threonine kinase),mechanistic target of rapamycin | TCACTCTTGCCCTCCGAACGCTTGGCAGCTTTGAATTTGAAGGCCACTCTCTGACCCAATTTGTTCGCCACTGTGCGGATCATTTCCTGAACAGTGAGCA | mechanistic target of rapamycin kinase |
| MUC5B | NM_002458.1 | MUC5;mucin 5, subtype B, tracheobronchial | GGGACCCGATGGGTTTCCTAAATTTCCCGGGGAGCGGTGGGTCAGCAACTGCCAGTCCTGCGTGTGTGACGAGGGTTCAGTGTCGGTGCAGTGCAAGCCC | mucin 5B, oligomeric mucus/gel-forming |
| MYD88 | NM_002468.3 | myeloid differentiation primary response gene (88),myeloid differentiation primary response 88 | ACGTTTTTCTAGGTACAGCTCCCAGGAACAGCTAGGTGGGAAAGTCCCATCACTGAGGGAGCCTAACCATGTCCCTGAACAAAAATTGGGCACTCATCTA | MYD88 innate immune signal transduction adaptor |
| MYL2 | NM_000432.3 | myosin, light polypeptide 2, regulatory, cardiac, slow,myosin, light chain 2, regulatory, cardiac, slow | GTGGCTATGGGTACTTCGTGGCCGCACATCCTACAGTTGGAAATCCATCCAGAGGCCATGTTCCAATAAACAGGAGGTCGTGTATTTGGTCACGACATTT | myosin light chain 2 |
| MYLK | NM_053032.2 | myosin, light polypeptide kinase | ATTATTAGTGATGTTTGCGGGGATGACGATGCCAAGTACACCTGCAAGGCTGTCAACAGTCTTGGAGAAGCCACCTGCACAGCAGAGCTCATTGTGGAAA | myosin light chain kinase |
| NCAM1 | NM_000615.5 |  | GGTATTTGCCTATCCCAGTGCCACGATCTCATGGTTTCGGGATGGCCAGCTGCTGCCAAGCTCCAATTACAGCAATATCAAGATCTACAACACCCCCTCT | neural cell adhesion molecule 1 |
| NCEH1 | NM_020792.4 | AADACL1;arylacetamide deacetylase-like 1 | TTTGAGGATGGCTTTCACGGATGTATGATTTTCACTAGCTGGCCCACCAACTTCTCAGTGGGAATCCGGACTAGGAATAGTTACATCAAGTGGCTAGATC | neutral cholesterol ester hydrolase 1 |
| NCF1 | NM_000265.4 | neutrophil cytosolic factor 1 (47kD, chronic granulomatous disease, autosomal 1) | TGAGCCTGCCCACCAAGATCTCCCGCTGTCCCCACCTCCTCGACTTCTTCAAGGTGCGCCCTGATGACCTCAAGCTCCCCACGGACAACCAGACAAAAAA | neutrophil cytosolic factor 1 |
| NCF4 | NM_000631.4 | neutrophil cytosolic factor 4 (40kD),neutrophil cytosolic factor 4, 40kDa | CGAGAGTGACTTTGAACAGCTTCCGGATGATGTTGCCATCTCGGCCAACATTGCTGACATCGAGGAGAAGAGAGGCTTCACCAGCCACTTTGTTTTCGTC | neutrophil cytosolic factor 4 |
| NCK2 | NM_001004720.2 |  | CCCGACGGCTTCTCTGCGAGTCTCTCTTTATGTTCAGGTCGCTTGGTCGGTTCGTCTCCCATTTGCCATCCAGGCCTCACACCCACACTCGAGCCCACCC | NCK adaptor protein 2 |
| NCKAP1L | NM_005337.4 | HEM1;hematopoietic protein 1 | CTGTTTAGCAGTTTGAAAGGGTATGGCAAGAGAGTGGCAGACATAAAGGAGAGCAAGGAACATGTAATTGCAAACAGTGGCCAGTTTCATTGTCAACGGC | NCK associated protein 1 like |
| NCOA2 | NM_006540.2 |  | GCTGGGAGGACCTGGTAAGAAGGTGTATTCAGAAGTTCCATGCGCAGCATGAAGGAGAATCTGTGTCCTATGCTAAGAGGCATCATCATGAAGTACTGAG | nuclear receptor coactivator 2 |
| NCOR2 | NM_006312.3 | nuclear receptor co-repressor 2 | GCAGCATCCCAAGAACTTTGGCCTGATCGCATCATTCCTGGAGAGGAAGACAGTGGCTGAGTGCGTCCTCTATTACTACCTGACTAAGAAGAATGAGAAC | nuclear receptor corepressor 2 |
| NCR1 | NM_004829.5 | LY94;lymphocyte antigen 94 (mouse) homolog (activating NK-receptor; NK-p46) | CGATGTTTTGGCTCCTATAACAACCATGCCTGGTCTTTCCCCAGTGAGCCAGTGAAGCTCCTGGTCACAGGCGACATTGAGAACACCAGCCTTGCACCTG | natural cytotoxicity triggering receptor 1 |
| NCSTN | NM_015331.2 |  | GTGCCCAAATGATGGGTTTGGTGTTTACTCCAATTCCTATGGGCCAGAGTTTGCTCACTGCAGAGAAATACAGTGGAATTCGCTGGGCAATGGTTTGGCT | nicastrin |
| NDUFA1 | NM_004541.3 | NADH dehydrogenase (ubiquinone) 1 alpha subcomplex, 1 (7.5kD, MWFE),NADH dehydrogenase (ubiquinone) 1 alpha subcomplex, 1, 7.5kDa | AAGGAAAAAAGGGTTGCTCATTTTGGGTATCACTGGAGTCTGATGGAAAGAGATAGGCGCATCTCTGGAGTTGATCGTTACTATGTGTCAAAGGGTTTGG | NADH:ubiquinone oxidoreductase subunit A1 |
| NDUFB5 | NM_002492.3 | NADH dehydrogenase (ubiquinone) 1 beta subcomplex, 5 (16kD, SGDH),NADH dehydrogenase (ubiquinone) 1 beta subcomplex, 5, 16kDa | ATTGATGCATGTGAGAGGAGATGGACCCTGGTATTACTATGAGACAATTGACAAGGAACTTATTGATCATTCTCCGAAAGCAACTCCTGACAATTAAGCA | NADH:ubiquinone oxidoreductase subunit B5 |
| NDUFB6 | NM_002493.5 | NADH dehydrogenase (ubiquinone) 1 beta subcomplex, 6 (17kD, B17),NADH dehydrogenase (ubiquinone) 1 beta subcomplex, 6, 17kDa | CGCTTAAGTAACTAGTCCGTAGTTCGAGGGTGCGCCGTGTCCTTTTGCGTTGGTACCAGCGGCGACATGACGGGGTACACTCCGGATGAGAAACTGCGGC | NADH:ubiquinone oxidoreductase subunit B6 |
| NDUFB8 | NM_001284367.1 | NADH dehydrogenase (ubiquinone) 1 beta subcomplex, 8 (19kD, ASHI),NADH dehydrogenase (ubiquinone) 1 beta subcomplex, 8, 19kDa | CCAGAAGAACGGGCCGCCGCCGCCAAGAAGTATAATATGCGTGTGGAAGACTACGAACCTTACCCGGATGATGGCATGGGGTATGGCGACTACCCGAAGC | NADH:ubiquinone oxidoreductase subunit B8 |
| NDUFC1 | NM_002494.2 | NADH dehydrogenase (ubiquinone) 1, subcomplex unknown, 1 (6kD, KFYI),NADH dehydrogenase (ubiquinone) 1, subcomplex unknown, 1, 6kDa | GGCTCCCGAGCGGCCCTTCAGTGCGATCAAAGTTCTACGTGCGAGAGCCGCCGAATGCCAAACCTGACTGGCTGAAAGTTGGGTTCACCTTGGGCACCAC | NADH:ubiquinone oxidoreductase subunit C1 |
| NDUFS3 | NM_004551.1 | NADH dehydrogenase (ubiquinone) Fe-S protein 3 (30kD) (NADH-coenzyme Q reductase),NADH dehydrogenase (ubiquinone) Fe-S protein 3, 30kDa (NADH-coenzyme Q reductase) | GTCCTGCTTCAATGAGTTAGAGGTCTGTATCCATCCTGATGGCGTCATCCCAGTGCTGACTTTCCTCAGGGATCACACCAATGCACAGTTCAAATCTCTG | NADH:ubiquinone oxidoreductase core subunit S3 |
| NDUFS5 | NM_004552.2 | NADH dehydrogenase (ubiquinone) Fe-S protein 5 (15kD) (NADH-coenzyme Q reductase),NADH dehydrogenase (ubiquinone) Fe-S protein 5, 15kDa (NADH-coenzyme Q reductase) | GTTCGGCCTTAACATAGATCGATGGTTGACAATCCAGAGTGGTGAACAGCCCTACAAGATGGCTGGTCGATGCCATGCTTTTGAAAAAGAATGGATAGAA | NADH:ubiquinone oxidoreductase subunit S5 |
| NEDD8 | NM_006156.2 | neural precursor cell expressed, developmentally down-regulated 8 | AGGTGGTCTTAGGCAGTGATGGACCCTCCATTTTACCTCTTTACCCTGTCGCTCATAATGAGGCATCATATATCCTCTCACTCTCTGGGACACCATAGCC | NEDD8 ubiquitin like modifier |
| NF1 | NM_000267.2 |  | TACCAGATCCCACAGACTGATATGGCTGAATGTGCAGAAAAGCTATTTGACTTGGTGGATGGTTTTGCTGAAAGCACCAAACGTAAAGCAGCAGTTTGGC | neurofibromin 1 |
| NFAM1 | NM_145912.5 |  | ATGTAGTTCAATAGTTTTCTTGGCAGACAGCTTATGCAGGAGAGGTGGGCCGTTGGCTCAGTCCAGCCAGGCTGAGTGGCGTCACTCCATTCCTTGCAAT | NFAT activating protein with ITAM motif 1 |
| NFKB1 | NM_003998.2 | nuclear factor of kappa light polypeptide gene enhancer in B-cells 1 | AGGGTATAGCTTCCCACACTATGGATTTCCTACTTATGGTGGGATTACTTTCCATCCTGGAACTACTAAATCTAATGCTGGGATGAAGCATGGAACCATG | nuclear factor kappa B subunit 1 |
| NID1 | NM_002508.2 | NID;nidogen (enactin) | AGGGCTCCAGTATCCTTTTGCTGTGACGAGCTACGGGAAGAATCTGTATTTCACAGACTGGAAGATGAATTCCGTGGTTGCTCTCGATCTTGCAATTTCC | nidogen 1 |
| NID2 | NM_007361.3 |  | GGCCTGTTTGGCTGGCTCTTTGCTTTAGAAAAACCTGGCTCTGAGAACGGCTTCAGCCTCGCAGGTGCTGCCTTTACCCATGACATGGAAGTTACATTCT | nidogen 2 |
| NKG7 | NM_005601.3 | natural killer cell group 7 sequence | CTGTGGCGGTCCCCGTCCTGGCTATGAAACCTTGTGAGCAGAAGGCAAGAGCGGCAAGATGAGTTTTGAGCGTTGTATTCCAAAGGCCTCATCTGGAGCC | natural killer cell granule protein 7 |
| NLRC4 | NM_001199138.1 | CARD12;caspase recruitment domain family, member 12 | TGGAATGTTCTGAATCGCGAAGAAGTAAACATCATTTGCTGCGAGAAGGTGGAGCAGGATGCTGCTAGAGGGATCATTCACATGATTTTGAAAAAGGGTT | NLR family CARD domain containing 4 |
| NLRP3 | NM_001079821.2 | C1orf7,CIAS1,DFNA34;cold autoinflammatory syndrome 1,deafness, autosomal dominant 34 | AGTGGGGTTCAGATAATGCACGTGTTTCGAATCCCACTGTGATATGCCAGGAAGACAGCATTGAAGAGGAGTGGATGGGTTTACTGGAGTACCTTTCGAG | NLR family pyrin domain containing 3 |
| NOS1 | NM_000620.4 | NOS;nitric oxide synthase 1 (neuronal) | CAGCCAATGTGCAGTTCACAGAGATATGCATACAGCAGGGCTGGAAACCGCCTAGAGGCCGCTTCGATGTCCTGCCGCTCCTGCTTCAGGCCAACGGCAA | nitric oxide synthase 1 |
| NOS3 | NM_000603.4 | nitric oxide synthase 3 (endothelial cell) | CCGGACCACCTCGTCCCTGTGGAAAGACAAGGCAGCAGTGGAAATCAACGTGGCCGTGCTGCACAGTTACCAGCTAGCCAAAGTCACCATCGTGGACCAC | nitric oxide synthase 3 |
| NOTCH1 | NM_017617.3 | TAN1;Notch (Drosophila) homolog 1 (translocation-associated),Notch homolog 1, translocation-associated (Drosophila),notch 1 | AGGCAAAGCTGGCTCACCTTCCGCACGCGGATTAATTTGCATCTGAAATAGGAAACAAGTGAAAGCATATGGGTTAGATGTTGCCATGTGTTTTAGATGG | notch receptor 1 |
| NOTCH2 | NM_024408.3 | Notch (Drosophila) homolog 2,Notch homolog 2 (Drosophila),notch 2 | AAGAGTCACCAAATTTTGAGAGTTATACTTGCTTGTGTGCTCCTGGCTGGCAAGGTCAGCGGTGTACCATTGACATTGACGAGTGTATCTCCAAGCCCTG | notch receptor 2 |
| NOTCH3 | NM_000435.2 | CADASIL;Notch (Drosophila) homolog 3,Notch homolog 3 (Drosophila),notch 3 | CTTTGGAGTCTGCCGTGATGGCATCAACCGCTACGACTGTGTCTGCCAACCTGGCTTCACAGGGCCCCTTTGTAACGTGGAGATCAATGAGTGTGCTTCC | notch receptor 3 |
| NOTCH4 | NM_004557.3 | INT3;Notch (Drosophila) homolog 4,Notch homolog 4 (Drosophila),notch 4 | ACCCCTAAGTTGGAACCAAGAATTGCAGGCATATGGGATGTAAGATGTTCTTTCCTATATATGGTTTCCAAAGGGTGCCCCTATGATCCATTGTCCCCAC | notch receptor 4 |
| NPC1 | NM_000271.3 | Niemann-Pick disease, type C1 | GACCTCTGCTGACTTTATTGACGCTCTGAAGAAAGCCCGACTTATAGCCAGTAATGTCACCGAAACCATGGGCATTAACGGCAGTGCCTACCGAGTATTT | NPC intracellular cholesterol transporter 1 |
| NPHP4 | NM_015102.3 | nephronophthisis 4 | TCAGCGCTACAGAACCTGTGACGTTTAACCCTCAGAAGGAAGAATCAGATTGTCTACAAAGCAACGAGATGGTGCTACAGTTTCTTGCCTTTAGCAGAGT | nephrocystin 4 |
| NR1H2 | NM_007121.4 | UNR;ubiquitously-expressed nuclear receptor,nuclear receptor subfamily 1, group H, member 2 | TGACGCAAGGGCGGGGTTGCCGGAAGAAGTGGCGAAGTTACTTTTGAGGGTATTTGAGTAGCGGCGGTGTGTCAGGGGCTAAAGAGGAGGACGAAGAAAA | nuclear receptor subfamily 1 group H member 2 |
| NR1H3 | NM_005693.2 | nuclear receptor subfamily 1, group H, member 3 | CCCATATTTTCTGTTTTCTTGGCCGGATGGCTGAGGCCTGGTGGCTGCCTCCTAGAAGTGGAACAGACTGAGAAGGGCAAACATTCCTGGGAGCTGGGCA | nuclear receptor subfamily 1 group H member 3 |
| NR1H4 | NM_001206977.1 | nuclear receptor subfamily 1, group H, member 4 | GGGCGCGTCAGCAGGGAGGATCAAAGGGGATGAGCTGTGTGTTGTTTGTGGAGACAGAGCCTCTGGATACCACTATAATGCACTGACCTGTGAGGGGTGT | nuclear receptor subfamily 1 group H member 4 |
| NRIP3 | NM_020645.2 | C11orf14;chromosome 11 open reading frame 14 | AGAAGTCTGAGGAGGATGACATGATTTTGGTTTCTTGCCAGTGTGCTGGAAAGGATGTGAAAGCCTTGGTTGACACAGGCTGCCTATATAATCTCATCTC | nuclear receptor interacting protein 3 |
| NRP2 | NM_003872.2 |  | TCTCACCTGGGTTTTCTTAGCCCTCTACTTTTCAAGACACCAAGTGAGAGGCCAACCAGACCCACCGTGCGGAGGTCGTTTGAATTCCAAAGATGCTGGC | neuropilin 2 |
| NUMB | NM_001005743.1 | C14orf41;numb (Drosophila) homolog,chromosome 14 open reading frame 41,numb homolog (Drosophila) | TCGCCAAGGCTCTTTCCGAGGTTTTCCTGCTCTTAGCCAGAAGATGTCACCCTTTAAACGCCAACTATCCCTACGCATCAATGAGTTGCCTTCCACTATG | NUMB endocytic adaptor protein |
| OAS1 | NM_001032409.1 | OIAS;2',5'-oligoadenylate synthetase 1 (40-46 kD),2'-5'-oligoadenylate synthetase 1, 40/46kDa | CTCCTGACGGTCTATGCTTGGGAGCGAGGGAGCATGAAAACACATTTCAACACAGCCCAGGGATTTCGGACGGTCTTGGAATTAGTCATAAACTACCAGC | 2'-5'-oligoadenylate synthetase 1 |
| OASL | NM_198213.1 | 2'-5'-oligoadenylate synthetase-like | GGCGTTTCTGAGCTGTTTCCACAGCTTCCAGGAGGCAGCCAAGCATCACAAAGATGTTCTGAGGCTGATATGGAAAACCATGTGGCAAAGCCAGGACCTG | 2'-5'-oligoadenylate synthetase like |
| OCLN | NM_002538.3 |  | GTTGGAGACTATGATAGACAGAAAACATAGAAGGCTGATGCCAAGTTGTTTGAGAAATTAAGTATCTGACATCTCTGCAATCTTCTCAGAAGGCAAATGA | occludin |
| OGT | NM_181673.2 | O-linked N-acetylglucosamine (GlcNAc) transferase (UDP-N-acetylglucosamine:polypeptide-N-acetylglucosaminyl transferase) | CCATAGCTTCTTACCGCACGGCTCTGAAACTTAAGCCTGATTTTCCTGATGCTTATTGTAACTTGGCTCATTGCCTGCAGATTGTCTGTGATTGGACAGA | O-linked N-acetylglucosamine (GlcNAc) transferase |
| OSBPL5 | NM_020896.3 | oxysterol binding protein-like 5 | AGCCACCGTCCACCCAGACCAAGACCTGTTCCCACTGAACGGGTCTTCCCTGGAGAACGATGCATTCTCAGACAAGTCGGAGAGAGAGAACCCTGAGGAG | oxysterol binding protein like 5 |
| P2RY13 | NM_176894.1 | GPR94,GPR86;G protein-coupled receptor 86,purinergic receptor P2Y, G-protein coupled, 13 | CACGAGCTCCAACACGACCATCGTAGGGTGAAGCCCACGTTTTCTTCCATGGCCTCAAAGGCCCTAGAACTTGCCTACCTTTCTGGCCTTACCTCCTAGC | purinergic receptor P2Y13 |
| P3H3 | NM_014262.4 | LEPREL2;leprecan-like 2 | GGCGGCTACATGAGGCCCATGCTCAGGTGGGCAATCTGTCCCAGGCTATAGAAAATGTCCTGAGTGTCCTGCTCTTCTACCCGGAGGATGAGGCTGCCAA | prolyl 3-hydroxylase 3 |
| P4HB | NM_000918.3 | PO4DB,ERBA2L;procollagen-proline, 2-oxoglutarate 4-dioxygenase (proline 4-hydroxylase), beta polypeptide (protein disulfide isomerase; thyroid hormone binding protein p55),procollagen-proline, 2-oxoglutarate 4-dioxygenase (proline 4-hydroxylase), beta polypeptide (protein disulfide isomerase-associated 1),procollagen-proline, 2-oxoglutarate 4-dioxygenase (proline 4-hydroxylase), beta polypeptide,prolyl 4-hydroxylase, beta polypeptide | GCCCACCCTGGTGGGGCTTGTTTCCTGAAACCATGATGTACTTTTTCATACATGAGTCTGTCCAGAGTGCTTGCTACCGTGTTCGGAGTCTCGCTGCCTC | prolyl 4-hydroxylase subunit beta |
| PANX1 | NM_015368.3 |  | TGGCCCACACTCGAGCTCTCTTTACATTGTTAGTTGTCAACCTTGGCTGATGGAAATCCCGTAACCACTATTTGTTGCACTGTGCCTTGAAGGGCAGCAG | pannexin 1 |
| PARP1 | NM_001618.3 | PPOL,ADPRT;ADP-ribosyltransferase (NAD+; poly (ADP-ribose) polymerase),poly (ADP-ribose) polymerase family, member 1 | AAGGTTTGGGCAAAACTACCCCTGATCCTTCAGCTAACATTAGTCTGGATGGTGTAGACGTTCCTCTTGGGACCGGGATTTCATCTGGTGTGAATGACAC | poly(ADP-ribose) polymerase 1 |
| PARP4 | NM_006437.3 | ADPRTL1;ADP-ribosyltransferase (NAD+; poly (ADP-ribose) polymerase)-like 1 | CATGGTTAATGTCTGTGAAACTAATTTGTCCAAACCCAACCCACCATCCCTGGCCAAATACCGAGCTTTGAGGTGCAAAATTGAGCATGTTGAACAGAAT | poly(ADP-ribose) polymerase family member 4 |
| PC | NM_000920.3 |  | CCCAGACAACGTGGTCTTCAAGTTCTGTGAAGTGGCCAAAGAGAATGGCATGGATGTCTTCCGTGTGTTTGACTCCCTCAACTACTTGCCCAACATGCTG | pyruvate carboxylase |
| PCCB | NM_000532.4 | propionyl Coenzyme A carboxylase, beta polypeptide | CAGATTTTGGAATGGCTGCTGATAAGAATAAGTTTCCTGGAGACAGCGTGGTCACTGGACGAGGCCGAATCAATGGAAGATTGGTTTATGTCTTCAGTCA | propionyl-CoA carboxylase subunit beta |
| PCK1 | NM_002591.2 | phosphoenolpyruvate carboxykinase 1 (soluble) | GGGCACATCAACATGATGGAGCTTTTCAGCATCTCCAAGGAATTCTGGGAGAAGGAGGTGGAAGACATCGAGAAGTATCTGGAGGATCAAGTCAATGCCG | phosphoenolpyruvate carboxykinase 1 |
| PDE2A | NM_001143839.1 | phosphodiesterase 2A, cGMP-stimulated | CATCCTCTGCTTCCCCATCAAGAACGAGAACCAGGAGGTCATCGGTGTGGCCGAGCTGGTGAACAAGATCAATGGGCCATGGTTCAGCAAGTTCGACGAG | phosphodiesterase 2A |
| PDE3B | NM_000922.3 | phosphodiesterase 3B, cGMP-inhibited | AGCTGGTGTTTTGTCCAGTCTGAGTCCTGTGAATTCTTCCAACCATGGACCAGTGTCTACTGGCTCTCTAACTAATCGATCACCCATAGAATTTCCTGAT | phosphodiesterase 3B |
| PDGFB | NM_033016.2 | SIS;platelet-derived growth factor beta polypeptide (simian sarcoma viral (v-sis) oncogene homolog),platelet-derived growth factor beta polypeptide | GATTCACCTCTTCCTCTGGTTCCTTTCATCTCTCTACCTCCACCCTGCATTTTCCTCTTGTCCTGGCCCTTCAGTCTGCTCCACCAAGGGGCTCTTGAAC | platelet derived growth factor subunit B |
| PDGFRB | NM_002609.3 | PDGFR;platelet-derived growth factor receptor, beta polypeptide | CCCCTTCCTCCATCCCTCTGTTCTCCTGAGCCTTCAGGAGCCTGCACCAGTCCTGCCTGTCCTTCTACTCAGCTGTTACCCACTCTGGGACCAGCAGTCT | platelet derived growth factor receptor beta |
| PDHA1 | NM_000284.3 | PDHA;pyruvate dehydrogenase (lipoamide) alpha 1,pyruvate dehydrogenase alpha 1 | TAAGAGTGACCCTATTATGCTTCTCAAGGACAGGATGGTGAACAGCAATCTTGCCAGTGTGGAAGAACTAAAGGAAATTGATGTGGAAGTGAGGAAGGAG | pyruvate dehydrogenase E1 alpha 1 subunit |
| PECAM1 | NM_000442.3 | platelet/endothelial cell adhesion molecule 1 | ATCTGCACTGCAGGTATTGACAAAGTGGTCAAGAAAAGCAACACAGTCCAGATAGTCGTATGTGAAATGCTCTCCCAGCCCAGGATTTCTTATGATGCCC | platelet and endothelial cell adhesion molecule 1 |
| PELI1 | NM_020651.3 | pellino (Drosophila) homolog 1,pellino homolog 1 (Drosophila) | TTAGCCCGTGTGGGCATGTGTGTTCAGAAAAGACAACTGCCTATTGGTCCCAGATCCCACTTCCTCATGGTACTCATACTTTTCATGCAGCCTGTCCCTT | pellino E3 ubiquitin protein ligase 1 |
| PELI2 | NM_021255.2 | pellino (Drosophila) homolog 2,pellino homolog 2 (Drosophila) | GACTGTGGTGGTGGAGTACACACATGATAAGGATACGGATATGTTTCAGGTGGGCAGATCAACAGAAAGCCCTATCGACTTCGTTGTCACAGACACGATT | pellino E3 ubiquitin protein ligase family member 2 |
| PGM1 | NM_001172818.1 |  | TGTGAGGGAAAGAGGACCTGCGGGCTTAGATCAATCTCAATTCCTTTTCATGCCCTCCTGCATTGCTGCTGCGTGGGTATTTGTCTCCTTAGCCATCAGG | phosphoglucomutase 1 |
| PHLPP1 | NM_194449.3 | PLEKHE1,PHLPP;pleckstrin homology domain containing, family E (with leucine rich repeats) member 1,PH domain and leucine rich repeat protein phosphatase | CATCCTATGTGGGACCTGCCTGATAGTATCATCTGTGAAAGACAGCTTGACCGGAAAGATGCATGTTCTGCCACTAATTGGTGGAAAAGTAGAAGAAGTG | PH domain and leucine rich repeat protein phosphatase 1 |
| PHYKPL | NM_153373.3 | AGXT2L2;alanine-glyoxylate aminotransferase 2-like 2 | ACCATGGGCAAGTCCATTGGCAACGGCCACCCTGTTGCCTGCGTGGCCGCAACCCAGCCTGTGGCGAGGGCATTTGAAGCCACCGGCGTTGAGTACTTCA | 5-phosphohydroxy-L-lysine phospho-lyase |
| PIDD1 | NM_145886.2 | LRDD,PIDD;leucine-rich repeats and death domain containing,p53-induced death domain protein | GGGCGAAGAGTTCTTTGCGGCCTTCGAGCGCGGCATCGACGTGGATGCTGACCGCCCTGACTGTGTGGAGGGCAGAATCTGCTTTGTCTTCTACTCGCAC | p53-induced death domain protein 1 |
| PIK3C3 | NM_002647.2 | phosphoinositide-3-kinase, class 3,phosphatidylinositol 3-kinase, catalytic subunit type 3 | GGACTCCTTGGAGCTGTTATCCTCTCATTACACCAACCCAACTGTGAGGCGTTATGCTGTTGCCCGGTTGCGACAGGCCGATGATGAGGATTTGTTGATG | phosphatidylinositol 3-kinase catalytic subunit type 3 |
| PIK3CA | NM_006218.2 | phosphoinositide-3-kinase, catalytic, alpha polypeptide,phosphatidylinositol-4,5-bisphosphate 3-kinase, catalytic subunit alpha | CCTCAGGCTTGAAGAGTGTCGAATTATGTCCTCTGCAAAAAGGCCACTGTGGTTGAATTGGGAGAACCCAGACATCATGTCAGAGTTACTGTTTCAGAAC | phosphatidylinositol-4,5-bisphosphate 3-kinase catalytic subunit alpha |
| PIK3CB | NM_006219.1 | PIK3C1;phosphoinositide-3-kinase, catalytic, beta polypeptide,phosphatidylinositol-4,5-bisphosphate 3-kinase, catalytic subunit beta | CCGCCAGTGTTGTGAGGATGCATATCTGATTTTACGACGGCATGGGAATCTCTTCATCACTCTCTTTGCGCTGATGTTGACTGCAGGGCTTCCTGAACTC | phosphatidylinositol-4,5-bisphosphate 3-kinase catalytic subunit beta |
| PIK3R4 | NM_014602.1 | phosphoinositide-3-kinase, regulatory subunit 4 | GGGAAGACCACCACTACCAGATCTATTCTTACATACAGCCGAATTGGAGGACGAGTCAAGACGCTCACATTCTGCCAAGGCTCCCACTATTTAGCCATAG | phosphoinositide-3-kinase regulatory subunit 4 |
| PIK3R5 | NM_001142633.1 | phosphoinositide-3-kinase, regulatory subunit 5 | TTTCTCTCAAGTTTCCTGAGTCTCCAGAAAAACAGCACTAACGCTGGACCTGTCTACTCTCAGAACCCGGCACAGATTCTCTCTTGATCTCCTTTTGGAA | phosphoinositide-3-kinase regulatory subunit 5 |
| PKHD1 | NM_138694.3 | TIGM1;TIG multiple domains 1,polycystic kidney and hepatic disease 1 (autosomal recessive),PKHD1, fibrocystin/polyductin | TATAATAAGAAACAACGTGATCATCCAGGTTTCTGGTGCCGAGGGACTCTCCAATCCTGAAATGTTGACACCATCTGGCATCTATATCTGCAGTCCCACC | PKHD1 ciliary IPT domain containing fibrocystin/polyductin |
| PLCB2 | NM_004573.2 | phospholipase C, beta 2 | TCCTCCGTGTGGATCCTAAGGGCTACTACTTATACTGGACGTATCAAAGTAAGGAGATGGAGTTTCTGGATATCACCAGCATCCGGGATACTCGCTTTGG | phospholipase C beta 2 |
| PLCB3 | NM_000932.2 | phospholipase C, beta 3 (phosphatidylinositol-specific) | GAAGCTGATGACGGTGGTGTCTGGGCCAGACCCAGTGAACACAGTGTTCTTGAACTTCATGGCCGTGCAGGATGACACAGCCAAGGTCTGGTCTGAGGAG | phospholipase C beta 3 |
| PLCG1 | NM_002660.2 | PLC1;phospholipase C, gamma 1 (formerly subtype 148),phospholipase C, gamma 1 | GTGATGCTAGGGAACTCGGAGTTCGACAGCCTTGTTGACCTCATCAGCTACTATGAGAAACACCCGCTATACCGCAAGATGAAGCTGCGCTATCCCATCA | phospholipase C gamma 1 |
| PLCG2 | NM_002661.2 | phospholipase C, gamma 2 (phosphatidylinositol-specific) | GCTTGAAAATCTTACACCAGGAAGCGATGAATGCGTCCACGCCCACCATTATCGAGAGTTGGCTGAGAAAGCAGATATATTCTGTGGATCAAACCAGAAG | phospholipase C gamma 2 |
| PLG | NM_000301.3 |  | GGAAGTGGTTCTTCTACTTCTTTTATTTCTGAAATCAGGTCAAGGAGAGCCTCTGGATGACTATGTGAATACCCAGGGGGCTTCACTGTTCAGTGTCACT | plasminogen |
| PLIN4 | NM_001080400.1 | KIAA1881;KIAA1881 | CGTGGCCAAGGGGGCTGTGCAAACTGGGCTGAAAACGACCCAAAATATCGCGACAGGTACAAAGAACACCTTTGGCAGTGGGGTGACCAGTGCTGTGAAT | perilipin 4 |
| PLPP4 | NM_001030059.1 | PPAPDC1,PPAPDC1A;phosphatidic acid phosphatase type 2 domain containing 1,phosphatidic acid phosphatase type 2 domain containing 1A | TTGAATTTGCAAGTGAAGGACAACAATCTCTGAGAGACGTGTGGAAGAGGCTGTGAAGGTGGGGTTTGGGGAGCTTGGCCGATTCGTCTATCTGAAATGT | phospholipid phosphatase 4 |
| PLTP | NM_006227.2 |  | GGTGCAGATCCCACTACCTGAGGGCATCAACTTTGTGCATGAGGTGGTGACGAACCATGCGGGATTCCTCACCATCGGGGCTGATCTCCACTTTGCCAAA | phospholipid transfer protein |
| PNOC | NM_001284244.1 |  | CGGGGCCCGGAAGTCGGCCAGGAAGTTGGCCAATCAGAAGCGGTTCAGTGAGTTTATGAGGCAATACTTGGTCCTGAGCATGCAGTCCAGCCAGCGCCGG | prepronociceptin |
| PNPLA3 | NM_025225.2 | C22orf20,ADPN;chromosome 22 open reading frame 20,adiponutrin,patatin-like phospholipase domain containing 3 | CCACCAGCTCATCTCCGGCAAAATAGGCATCTCTCTTACCAGAGTGTCTGATGGGGAAAACGTTCTGGTGTCTGACTTTCGGTCCAAAGACGAAGTCGTG | patatin like phospholipase domain containing 3 |
| POSTN | NM_001135935.1 | periostin, osteoblast specific factor | AGAGACGGTCACTTCACACTCTTTGCTCCCACCAATGAGGCTTTTGAGAAACTTCCACGAGGTGTCCTAGAAAGGATCATGGGAGACAAAGTGGCTTCCG | periostin |
| PPARA | NM_001001928.2 | PPAR;peroxisome proliferative activated receptor, alpha,peroxisome proliferator-activated receptor alpha | CAAGAAATGGGAAACATCCAAGAGATTTCGCAATCCATCGGCGAGGATAGTTCTGGAAGCTTTGGCTTTACGGAATACCAGTATTTAGGAAGCTGTCCTG | peroxisome proliferator activated receptor alpha |
| PPARD | NM_006238.4 | peroxisome proliferative activated receptor, delta,peroxisome proliferator-activated receptor delta | AGGCCCGCAGCATCCTCACCGGCAAAGCCAGCCACACGGCGCCCTTTGTGATCCACGACATCGAGACATTGTGGCAGGCAGAGAAGGGGCTGGTGTGGAA | peroxisome proliferator activated receptor delta |
| PPARG | NM_005037.5 | peroxisome proliferative activated receptor, gamma,peroxisome proliferator-activated receptor gamma | CAGATCCAGTGGTTGCAGATTACAAGTATGACCTGAAACTTCAAGAGTACCAAAGTGCAATCAAAGTGGAGCCTGCATCTCCACCTTATTATTCTGAGAA | peroxisome proliferator activated receptor gamma |
| PPARGC1A | NM_013261.3 | PPARGC1;peroxisome proliferative activated receptor, gamma, coactivator 1,peroxisome proliferative activated receptor, gamma, coactivator 1, alpha,peroxisome proliferator-activated receptor gamma, coactivator 1 alpha | GAACAAGCACTTCGGTCATCCCAGTCAAGCTGTTTTTGACGACGAAGCAGACAAGACCGGTGAACTGAGGGACAGTGATTTCAGTAATGAACAATTCTCC | PPARG coactivator 1 alpha |
| PPM1A | NM_021003.4 | protein phosphatase 1A (formerly 2C), magnesium-dependent, alpha isoform,protein phosphatase, Mg2+/Mn2+ dependent, 1A | TTGAAATGGAGGATGCACATACGGCTGTGATCGGTTTGCCAAGTGGACTTGAATCGTGGTCATTCTTTGCTGTGTATGATGGGCATGCTGGTTCTCAGGT | protein phosphatase, Mg2+/Mn2+ dependent 1A |
| PPP2CA | NM_002715.2 | protein phosphatase 2 (formerly 2A), catalytic subunit, alpha isoform,protein phosphatase 2, catalytic subunit, alpha isozyme | GACATTTAATCATGCCAATGGCCTCACGTTGGTGTCTAGAGCTCACCAGCTAGTGATGGAGGGATATAACTGGTGCCATGACCGGAATGTAGTAACGATT | protein phosphatase 2 catalytic subunit alpha |
| PRAP1 | NM_145202.4 |  | TGAGGAGGCTCCTCCTGGTCACCAGCCTGGTGGTTGTGCTGCTGTGGGAGGCAGGTGCAGTCCCAGCACCCAAGGTCCCTATCAAGATGCAAGTCAAACA | proline rich acidic protein 1 |
| PRDX1 | NM_002574.2 | PAGA | GACCCATGAACATTCCTTTGGTATCAGACCCGAAGCGCACCATTGCTCAGGATTATGGGGTCTTAAAGGCTGATGAAGGCATCTCGTTCAGGGGCCTTTT | peroxiredoxin 1 |
| PRDX6 | NM_004905.2 |  | GTGATAAGTTTCTATCAAAATGGGGAGATTGCAGAAAAGGCTTCCCTTGGCTCCCAAGGAGGTGTAGCAGGTGTGAGCAATATTAGTGCCATGTGCCTTT | peroxiredoxin 6 |
| PREX1 | NM_020820.3 | phosphatidylinositol-3,4,5-trisphosphate-dependent Rac exchange factor 1 | CGGGTTAGAGGGTCACTGGGAAACACCGGGCGGTGGCTTCTGTGATTTATTTTCTTGATGGTAACTTCTCAGAGCAGGGCGATTGGGACATCACCAGCCA | phosphatidylinositol-3,4,5-trisphosphate dependent Rac exchange factor 1 |
| PRF1 | NM_005041.3 | perforin 1 (pore forming protein) | ACTGTTTTTCAGGGAGGTGGCTGGGTTTACACGCTAATCCCGATTCACCCTGTCCAAACTGCCTAAGCCCTCCGCCATTCTCAAGCCCTGCAGTCACAGC | perforin 1 |
| PRKAA1 | NM_006251.5 | protein kinase, AMP-activated, alpha 1 catalytic subunit | AGGCTGGATGAAAAAGAAAGTCGGCGTCTGTTCCAACAGATCCTTTCTGGTGTGGATTATTGTCACAGGCATATGGTGGTCCATAGAGATTTGAAACCTG | protein kinase AMP-activated catalytic subunit alpha 1 |
| PRKAB2 | NM_005399.3 | protein kinase, AMP-activated, beta 2 non-catalytic subunit | AAGTGACTTTGAAAAGTTTTGTGGCACCTGACCCACCCCAGACACTAGGGCTATCAGAAGGTCTCCTTTTTAGCCCAGCACAGGCCCAGGCCACTTTGTC | protein kinase AMP-activated non-catalytic subunit beta 2 |
| PRKACA | NM_002730.3 | protein kinase, cAMP-dependent, catalytic, alpha,protein kinase, cAMP-dependent, alpha catalytic subunit | GAACCACTATGCCATGAAGATCCTCGACAAACAGAAGGTGGTGAAACTGAAACAGATCGAACACACCCTGAATGAAAAGCGCATCCTGCAAGCTGTCAAC | protein kinase cAMP-activated catalytic subunit alpha |
| PRKACB | NM_182948.2 | protein kinase, cAMP-dependent, catalytic, beta,protein kinase, cAMP-dependent, beta catalytic subunit | GAGTTAAAGGCAGAACTTGGACATTATGTGGAACTCCAGAGTATTTGGCTCCAGAAATAATTCTCAGCAAGGGCTACAATAAGGCAGTGGATTGGTGGGC | protein kinase cAMP-activated catalytic subunit beta |
| PRKAG1 | NM_002733.3 | protein kinase, AMP-activated, gamma 1 non-catalytic subunit | AAGACCTACAACAACCTAGATGTATCTGTGACTAAAGCCTTGCAACATCGATCACATTACTTTGAGGGTGTTCTCAAGTGCTACCTGCATGAGACTCTGG | protein kinase AMP-activated non-catalytic subunit gamma 1 |
| PRKAG2 | NM_016203.3 | protein kinase, AMP-activated, gamma 2 non-catalytic subunit | GGAATAGGAACGTACCACAACATTGCCTTCATACATCCAGACACTCCCATCATCAAAGCCTTGAACATATTTGTGGAAAGACGAATATCAGCTCTGCCTG | protein kinase AMP-activated non-catalytic subunit gamma 2 |
| PRKCA | NM_002737.2 | PKCA;protein kinase C, alpha | AAAACCATCCGCTCCACACTAAATCCGCAGTGGAATGAGTCCTTTACATTCAAATTGAAACCTTCAGACAAAGACCGACGACTGTCTGTAGAAATCTGGG | protein kinase C alpha |
| PRKDC | NM_006904.6 | HYRC,HYRC1;protein kinase, DNA-activated, catalytic polypeptide | GCAAGTTAGTGAAACAGCTGTCTCCGTAAATGGAGGAAATGTGGGGAAGCCTTGGAATGCCCTTCTGGTTCTGGCACATTGGAAAGCACACTCAGAAGGC | protein kinase, DNA-activated, catalytic subunit |
| PRLR | NM_001204318.1 |  | ACCTACCCTGATTGACTTAAAAACTGGTTGGTTCACGCTCCTGTATGAAATTCGATTAAAACCCGAGAAAGCAGCTGAGTGGGAGATCCATTTTGCTGGG | prolactin receptor |
| PROS1 | NM_000313.1 | PROS;protein S (alpha) | ATAGATGAATGCTCTGAGAACATGTGTGCTCAGCTTTGTGTCAATTACCCTGGAGGTTACACTTGCTATTGTGATGGGAAGAAAGGATTCAAACTTGCCC | protein S |
| PSEN2 | NM_000447.2 | AD4;Alzheimer disease 4 | CTGCTACAAGTTCATCCATGGCTGGTTGATCATGTCTTCACTGATGCTGCTGTTCCTCTTCACCTATATCTACCTTGGGGAAGTGCTCAAGACCTACAAT | presenilin 2 |
| PSENEN | NM_172341.1 | presenilin enhancer 2 homolog (C. elegans) | GGAAGTACTACCTGGGGGGGTTTGCTTTCCTGCCTTTTCTCTGGTTGGTCAACATCTTCTGGTTCTTCCGAGAGGCCTTCCTTGTCCCAGCCTACACAGA | presenilin enhancer, gamma-secretase subunit |
| PSMB1 | NM_002793.2 | proteasome (prosome, macropain) subunit, beta type, 1 | AAGGCAGCCATCTCGCCGTGAGACAGCAAGTGTCGCGCAGCCGTGCGATGTTGTCCTCTACAGCCATGTATTCGGCTCCTGGCAGAGACTTGGGGATGGA | proteasome subunit beta 1 |
| PSMB7 | NM_002799.2 | proteasome (prosome, macropain) subunit, beta type, 7 | GTTACATTGGTGCAGCCCTAGTTTTAGGGGGAGTAGATGTTACTGGACCTCACCTCTACAGCATCTATCCTCATGGATCAACTGATAAGTTGCCTTATGT | proteasome subunit beta 7 |
| PSMB8 | NM_004159.4 | LMP7;proteasome (prosome, macropain) subunit, beta type, 8 (large multifunctional protease 7),large multifunctional peptidase 7,proteasome (prosome, macropain) subunit, beta type, 8 | ACTCACAGAGACAGCTATTCTGGAGGCGTTGTCAATATGTACCACATGAAGGAAGATGGTTGGGTGAAAGTAGAAAGTACAGATGTCAGTGACCTGCTGC | proteasome subunit beta 8 |
| PSMC3 | NM_002804.4 | proteasome (prosome, macropain) 26S subunit, ATPase, 3 | GAGTCGGGATTTGTGGGGAGAGGTTTTCCACTGGTCAAGAGAAGGCTTTAAGAAAGACGGTATTAATCTCCCGTTGCGGCTCCCGCCTGGTCCCATCTTC | proteasome 26S subunit, ATPase 3 |
| PSMD13 | NM_175932.2 | proteasome (prosome, macropain) 26S subunit, non-ATPase, 13 | ATGCTCAACAACCTTCCTGGTGTGACATCGGTTCACAGTCGTTTCTATGATCTCTCCAGTAAATACTATCAAACAATCGGAAACCACGCGTCCTACTACA | proteasome 26S subunit, non-ATPase 13 |
| PTAFR | NM_000952.3 |  | CCTCACCGAAAAGTTCTACAGCATGCGCAGTAGCCGGAAATGCTCCCGGGCCACCACGGATACGGTCACTGAAGTGGTTGTGCCATTCAACCAGATCCCT | platelet activating factor receptor |
| PTCH1 | NM_000264.4 | NBCCS,PTCH;patched (Drosophila) homolog,patched homolog (Drosophila),patched homolog 1 (Drosophila) | CCGGAAAGCGCCGCTGTGGCTGAGAGCGAAGTTTCAGAGACTCTTATTTAAACTGGGTTGTTACATTCAAAAAAACTGCGGCAAGTTCTTGGTTGTGGGC | patched 1 |
| PTGER4 | NM_000958.2 | prostaglandin E receptor 4 (subtype EP4) | CACTACGTGGACAAGCGATTGGCGGGCCTCACGCTCTTTGCAGTCTATGCGTCCAACGTGCTCTTTTGCGCGCTGCCCAACATGGGTCTCGGTAGCTCGC | prostaglandin E receptor 4 |
| PTGS2 | NM_000963.1 | prostaglandin-endoperoxide synthase 2 (prostaglandin G/H synthase and cyclooxygenase) | GCTACAAAAGCTGGGAAGCCTTCTCTAACCTCTCCTATTATACTAGAGCCCTTCCTCCTGTGCCTGATGATTGCCCGACTCCCTTGGGTGTCAAAGGTAA | prostaglandin-endoperoxide synthase 2 |
| PTK2 | NM_005607.4 | PTK2 protein tyrosine kinase 2 | TAGGAGCACGTCTTGCTACCCTCTTTTGAAGATGTTCTCTAGCCTTCCACCAGCAGCGAGGAATTAACCCTGTGTCCTCAGTCGCCAGCACTTACAGCTC | protein tyrosine kinase 2 |
| PTK2B | NM_004103.3 | FAK2;FAK2 | CCAGTAGATGTGGAAAAGGAGGACGTGCGTATCCTCAAGGTCTGCTTCTATAGCAACAGCTTCAATCCTGGGAAAAACTTCAAACTGGTCAAATGCACTG | protein tyrosine kinase 2 beta |
| PTPA | NM_021131.4 | PPP2R4;protein phosphatase 2A, regulatory subunit B' (PR 53),protein phosphatase 2A activator, regulatory subunit 4,protein phosphatase 2 regulatory subunit 4 | CACTTTGTGGATGAGAAGGCCGTGAATGAGAACCACAAGGACTACATGTTCCTGGAGTGTATCCTGTTTATTACCGAGATGAAGACTGGCCCATTTGCAG | protein phosphatase 2 phosphatase activator |
| PTPN1 | NM_002827.3 | PTP1B | ATACAGTGCGACAGCTAGAATTGGAAAACCTTACAACCCAAGAAACTCGAGAGATCTTACATTTCCACTATACCACATGGCCTGACTTTGGAGTCCCTGA | protein tyrosine phosphatase non-receptor type 1 |
| PTPN11 | NM_002834.3 | NS1;Noonan syndrome 1,protein tyrosine phosphatase, non-receptor type 11 | TGTCAAATACTGGCCTGATGAGTATGCTCTAAAAGAATATGGCGTCATGCGTGTTAGGAACGTCAAAGAAAGCGCCGCTCATGACTATACGCTAAGAGAA | protein tyrosine phosphatase non-receptor type 11 |
| PTPN12 | NM_002835.3 |  | GACATGATTCAGGGAGCTAGAAGACACTTTAAGTTATACTGGAAAATTCAGGTGCCACTGAAAGCCAGATTTATAGTATTCCATCTTTAATATGTGGGAC | protein tyrosine phosphatase non-receptor type 12 |
| PTPN6 | NM_002831.5 |  | TGGTGCAGACGGAGGCGCAGTACAAGTTCATCTACGTGGCCATCGCCCAGTTCATTGAAACCACTAAGAAGAAGCTGGAGGTCCTGCAGTCGCAGAAGGG | protein tyrosine phosphatase non-receptor type 6 |
| PTPRC | NM_080923.2 | CD45 | GACACGGCTGACTTCCAGATATGACCATGTATTTGTGGCTTAAACTCTTGGCATTTGGCTTTGCCTTTCTGGACACAGAAGTATTTGTGACAGGGCAAAG | protein tyrosine phosphatase receptor type C |
| PYGM | NM_001164716.1 | phosphorylase, glycogen; muscle | TCCTTTGTGGATGATGAAGCTTTCATTCGGGATGTGGCCAAAGTGAAGCAGGAAAACAAGTTGAAGTTTGCTGCCTACCTAGAGAGGGAATACAAAGTCC | glycogen phosphorylase, muscle associated |
| RAB7B | NM_001164522.1 |  | ACAAGGGCTCCGATGGCTGCATCCTAGCTTTTGATGTCACCGACCTGGAGTCTTTTGAAGCCCTGGATATCTGGCGGGGTGATGTCCTGGCCAAGATTGT | RAB7B, member RAS oncogene family |
| RAC1 | NM_006908.4 | ras-related C3 botulinum toxin substrate 1 (rho family, small GTP binding protein Rac1) | CTGCATCATTTGAAAATGTCCGTGCAAAGTGGTATCCTGAGGTGCGGCACCACTGTCCCAACACTCCCATCATCCTAGTGGGAACTAAACTTGATCTTAG | Rac family small GTPase 1 |
| RAC2 | NM_002872.3 | ras-related C3 botulinum toxin substrate 2 (rho family, small GTP binding protein Rac2) | GCTGCCACAACTTGTGTACCTTCAGGGATGGGGCTCTTACTCCCTCCTGAGGCCAGCTGCTCTAATATCGATGGTCCTGCTTGCCAGAGAGTTCCTCTAC | Rac family small GTPase 2 |
| RAC3 | NM_005052.2 | ras-related C3 botulinum toxin substrate 3 (rho family, small GTP binding protein Rac3) | GCCTGAGGGCTGGCGGGGAGCAGCCCTGGACGTGTCCGCTGTTGTGTTGAGACGTGTGGTGTCCCTGAGTCGGCTGTGGGGAGCGGTGGGGGTGGGCCGG | Rac family small GTPase 3 |
| RAP1B | NM_001010942.2 |  | ATCTTTTATGACCTAGTGCGGCAAATTAACAGAAAAACTCCAGTGCCTGGGAAGGCTCGCAAAAAGTCATCATGTCAGCTGCTTTAATATACTAAATGCA | RAP1B, member of RAS oncogene family |
| RAPGEF1 | NM_005312.2 | GRF2;guanine nucleotide-releasing factor 2 (specific for crk proto-oncogene),Rap guanine nucleotide exchange factor (GEF) 1 | GGTGATTTTACTGCTCCTGAGTCAACCGGTGACCCAGAAAAACCACCTCCTCTACCAGAGAAGAAAAACAAACACATGCTGGCCTACATGCAGTTGCTGG | Rap guanine nucleotide exchange factor 1 |
| RAPGEF2 | NM_014247.2 | PDZGEF1;PDZ domain containing guanine nucleotide exchange factor (GEF) 1,Rap guanine nucleotide exchange factor (GEF) 2 | CCAGCACATAAAATCAACCAGGGACTACAGGTTCCCGCCGTGTCCCTTTATCCTTCACGGAAGAAAGTGCCCGTAAAGGATCTCCCACCTTTTGGCATAA | Rap guanine nucleotide exchange factor 2 |
| RASGRP4 | NM_170604.1 |  | TGGAGTCTGTGTTCAAGAATTATGACCCTGAAGGCCGAGGAACAATCTCTCAGGAGGACTTTGAGCGACTCTCGGGCAATTTTCCCTTCGCCTGCCATGG | RAS guanyl releasing protein 4 |
| RBX1 | NM_014248.2 |  | ATTATGGATCTTTGCATAGAATGTCAAGCTAACCAGGCGTCCGCTACTTCAGAAGAGTGTACTGTCGCATGGGGAGTCTGTAACCATGCTTTTCACTTCC | ring-box 1 |
| RELA | NM_021975.3 | NFKB3;nuclear factor of kappa light polypeptide gene enhancer in B-cells 3,v-rel avian reticuloendotheliosis viral oncogene homolog A | GAAGCATTAACTTCTCTGGAAAGGGGGGAGCTGGGGAAACTCAAACTTTTCCCCTGTCCTGATGGTCAGCTCCCTTCTCTGTAGGGAACTCTGGGGTCCC | RELA proto-oncogene, NF-kB subunit |
| RELB | NM_006509.3 | v-rel avian reticuloendotheliosis viral oncogene homolog B (nuclear factor of kappa light polypeptide gene enhancer in B-cells 3) | AGCAGGGACAGATGCGCCGGATGGATCCTGTGCTTTCCGAGCCCGTCTATGACAAGAAATCCACAAACACATCAGAGCTGCGGATTTGCCGAATTAACAA | RELB proto-oncogene, NF-kB subunit |
| RELN | NM_005045.2 |  | GGCAACCCCACCTACTACGTTCCGGGACAAGAATACCATGTGACAATTTCAACAAGCACCTTTTTTGACGGCTTGCTGGTGACAGGACTATACACATCTA | reelin |
| RETN | NM_020415.2 |  | ATGGAAGAAGCCATCAATGAGAGGATCCAGGAGGTCGCCGGCTCCCTAATATTTAGGGCAATAAGCAGCATTGGCCTGGAGTGCCAGAGCGTCACCTCCA | resistin |
| RGS7 | NM_002924.2 | regulator of G-protein signalling 7,regulator of G-protein signaling 7 | ATACGACCCGTTTCTTTTGCCACCTGACCCTTCTAACCCATGGCTGTCCGATGACACCACTTTCTGGGAACTTGAGGCAAGCAAAGAACCGAGCCAGCAG | regulator of G protein signaling 7 |
| RHO | NM_000539.2 | RP4;retinitis pigmentosa 4, autosomal dominant | ACATCTTCACCCACCAGGGCTCCAACTTCGGTCCCATCTTCATGACCATCCCAGCGTTCTTTGCCAAGAGCGCCGCCATCTACAACCCTGTCATCTATAT | rhodopsin |
| RIPK3 | NM_006871.3 | receptor-interacting serine-threonine kinase 3 | TAACAGGGCGACCGCTCGTTAACATATACAACTGCTCTGGGGTGCAAGTTGGAGACAACAACTACTTGACTATGCAACAGACAACTGCCTTGCCCACATG | receptor interacting serine/threonine kinase 3 |
| RNF111 | NM_017610.6 |  | CATTTTGAATGCTAAAAGTAGAAGCCATAGTGCACGGTCTCATAAGTGGCCTCGGACTGAGACAGAATCTGTATCGGGATTGTTAATGAAAAGACCCTGT | ring finger protein 111 |
| RNF152 | NM_173557.2 |  | AGCTCCACCTGGTCGGGGGTGTGCACTGTCATCTTGGTGGCTTGCGTCTTGGTCTTCCTCCTCGGCATCGTGCTTCACAACATGTCTTGCATTTCTAAGC | ring finger protein 152 |
| ROCK2 | NM_004850.3 |  | CAGCAGAAGAAACAGGAATTACAGGATGAACGGGACTCTTTGGCTGCCCAACTGGAGATCACCTTGACCAAAGCAGATTCTGAGCAACTGGCTCGTTCAA | Rho associated coiled-coil containing protein kinase 2 |
| RORA | NM_134261.2 | RAR-related orphan receptor A | AAAATTAACCGAGACACTTTATATGGCCCTGCACAGACCTGGAGCGCCACACACTGCACATCTTTTGGTGATCGGGGTCAGGCAAAGGAGGGGAAACAAT | RAR related orphan receptor A |
| RPS27A | NM_002954.5 |  | GAATGGCAAAATTAGTCGCCTTCGTCGAGAGTGCCCTTCTGATGAATGTGGTGCTGGGGTGTTTATGGCAAGTCACTTTGACAGACATTATTGTGGCAAA | ribosomal protein S27a |
| RPS6KA2 | NM_021135.4 | ribosomal protein S6 kinase, 90kD, polypeptide 2 | CCGCGGGTCCTCTCATACATGGCTTCTGTTTCTGCCGAGAGATCTGTTTTCCAATTATGAAGCCGGTCGGTTTGGTCAGACTCCCGACACCCACGTCCCA | ribosomal protein S6 kinase A2 |
| RPS6KB2 | NM_003952.2 | ribosomal protein S6 kinase, 70kD, polypeptide 2 | TCAAAAAGTTTCTGAAACGGAATCCCAGCCAGCGGATTGGGGGTGGCCCAGGGGATGCTGCTGATGTGCAGAGACATCCCTTTTTCCGGCACATGAATTG | ribosomal protein S6 kinase B2 |
| S100A12 | NM_005621.1 | S100 calcium-binding protein A12 (calgranulin C) | CAAGATGAACAGGTCGACTTTCAAGAATTCATATCCCTGGTAGCCATTGCGCTGAAGGCTGCCCATTACCACACCCACAAAGAGTAGGTAGCTCTCTGAA | S100 calcium binding protein A12 |
| S100A4 | NM_002961.2 | MTS1,CAPL;metastasin 1,calcium placental protein,S100 calcium-binding protein A4 (calcium protein, calvasculin, metastasin, murine placental homolog) | CAGGGACAACGAGGTGGACTTCCAAGAGTACTGTGTCTTCCTGTCCTGCATCGCCATGATGTGTAACGAATTCTTTGAAGGCTTCCCAGATAAGCAGCCC | S100 calcium binding protein A4 |
| SAA1 | NM_199161.1 | SAA | AGCTTCTTTTCGTTCCTTGGCGAGGCTTTTGATGGGGCTCGGGACATGTGGAGAGCCTACTCTGACATGAGAGAAGCCAATTACATCGGCTCAGACAAAT | serum amyloid A1 |
| SCD | NM_005063.4 | SCDOS;stearoyl-CoA desaturase opposite strand,stearoyl-CoA desaturase (delta-9-desaturase) | GTTGATTATCTTCAGCCCAGGCTTTTGCTAGATGGAATGGAAAAGCAACTTCATTTGACACAAAGCTTCTAAAGCAGGTAAATTGTCGGGGGAGAGAGTT | stearoyl-CoA desaturase |
| SCIN | NM_033128.3 |  | TGATGGTTCTGGCAAAGTGGAGATTTGGCGTGTAGAAAACAATGGTAGGATCCAAGTTGACCAAAACTCATATGGTGAATTCTATGGTGGTGACTGCTAC | scinderin |
| SDC3 | NM_014654.3 | syndecan 3 (N-syndecan) | GAGGGTGTCCCTTGTCACCAGCCTGTTTTGTCCTGGTCTCTCTGGGGTTGTTGAATCTCTCCTCTTGCCTGCCAAGTACACATGTACCCAGACTTCATTT | syndecan 3 |
| SEC24C | NM_004922.2 | SEC24 (S. cerevisiae) related gene family, member C,SEC24 family, member C (S. cerevisiae),SEC24 family member C | CTGGTGGCTCTGTCTACAAATATGCTTCCTTTCAGGTGGAGAACGACCAGGAGCGGTTCCTGAGTGACCTGCGTCGTGATGTCCAGAAGGTTGTTGGCTT | SEC24 homolog C, COPII coat complex component |
| SEC61B | NM_006808.2 | Sec61 beta subunit | GGGGCAAGTACACTCGTTCGTAGATTCAGTTACATCCATCTGTCATCTGAAGAAGGAGGAAAAAACCCAACATTTCTTGGACCAAAAGTATAGTGACTAT | SEC61 translocon beta subunit |
| SEH1L | NM_001013437.1 | SEH1-like (S. cerevisiae),SEH1-like nucleoporin | TGCTCCCAAGCACATGGGTCTTATGTTAGCAACCTGTTCCGCAGATGGTATAGTAAGAATCTATGAGGCACCAGATGTTATGAATCTCAGCCAGTGGTCT | SEH1 like nucleoporin |
| SELENOS | NM_203472.1 | VIMP;VCP-interacting membrane protein,VCP interacting membrane selenoprotein | GAAATGCAAAGAAGCCCCAGGAGGAAGACAGTCCTGGGCCTTCCACTTCATCTGTCCTGAAACGGAAATCGGACAGAAAGCCTTTGCGGGGAGGAGGTTA | selenoprotein S |
| SEM1 | NM_001201451.1 | SHFD1,SHFM1,C7orf76;split hand/foot malformation (ectrodactyly) type 1,chromosome 7 open reading frame 76,SEM1, 26S proteasome complex subunit | TGGTTCCAGAGTGAAAATAGAGCCCAGTAGAGTCTGTAGCTGATGCAATATGGACATGTAGGGTGAGTGAGAAAATGCTTTTGTTGGGTTAAGCATCTGA | SEM1 26S proteasome complex subunit |
| SERPINE1 | NM_000602.2 | PLANH1,PAI1;serine (or cysteine) proteinase inhibitor, clade E (nexin, plasminogen activator inhibitor type 1), member 1 | TGTGTTCAATAGATTTAGGAGCAGAAATGCAAGGGGCTGCATGACCTACCAGGACAGAACTTTCCCCAATTACAGGGTGACTCACAGCCGCATTGGTGAC | serpin family E member 1 |
| SERPINF1 | NM_002615.4 | PEDF;serine (or cysteine) proteinase inhibitor, clade F (alpha-2 antiplasmin, pigment epithelium derived factor), member 1,pigment epithelium-derived factor | GTCGGACCCTAAGGCTGTTTTACGCTATGGCTTGGATTCAGATCTCAGCTGCAAGATTGCCCAGCTGCCCTTGACCGGAAGCATGAGTATCATCTTCTTC | serpin family F member 1 |
| SERPING1 | NM_000062.2 | C1NH;serine (or cysteine) proteinase inhibitor, clade G (C1 inhibitor), member 1, (angioedema, hereditary),serpin peptidase inhibitor, clade G (C1 inhibitor), member 1 | GACAGAGGCGAAGGGAAGGTCGCAACAACAGTTATCTCCAAGATGCTATTCGTTGAACCCATCCTGGAGGTTTCCAGCTTGCCGACAACCAACTCAACAA | serpin family G member 1 |
| SERPINH1 | NM_001235.2 | CBP1,CBP2,SERPINH2;serine (or cysteine) proteinase inhibitor, clade H (heat shock protein 47), member 2,serine (or cysteine) proteinase inhibitor, clade H (heat shock protein 47), member 1, (collagen binding protein 1),serpin peptidase inhibitor, clade H (heat shock protein 47), member 1, (collagen binding protein 1) | ATGGTGGACAACCGTGGCTTCATGGTGACTCGGTCCTATACCGTGGGTGTCATGATGATGCACCGGACAGGCCTCTACAACTACTACGACGACGAGAAGG | serpin family H member 1 |
| SH2D1A | NM_002351.4 | IMD5,LYP;lymphoproliferative syndrome,SH2 domain protein 1A | GCTGTATCACGGTTACATTTATACATACCGAGTGTCCCAGACAGAAACAGGTTCTTGGAGTGCTGAGACAGCACCTGGGGTACATAAAAGATATTTCCGG | SH2 domain containing 1A |
| SIGLEC5 | NM_003830.3 | CD33L2;sialic acid binding Ig-like lectin 5 | GTCCTGCTGCTGCAAGGGAGATCGAACCTCGGGACAGGAGTGGTTCCTGCAGCCCTTGGTGGTGCTGGTGTCATGGCCCTGCTCTGTATCTGTCTGTGCC | sialic acid binding Ig like lectin 5 |
| SIL1 | NM_001037633.1 | MSS;Marinesco-Sjogren syndrome,SIL1 homolog, endoplasmic reticulum chaperone (S. cerevisiae) | ATGAGCTGAATGTTGTCATTGAGACTGACATGCAGATCATGGTACGGCTGATCAACAAGTTCAATAGTTCCAGCTCCAGTTTGGAAGAGAAGATTGCTGC | SIL1 nucleotide exchange factor |
| SIRT1 | NM_012238.4 | sirtuin (silent mating type information regulation 2, S. cerevisiae, homolog) 1,sirtuin (silent mating type information regulation 2 homolog) 1 (S. cerevisiae) | GGGTGTCTGTTTCATGTGGAATACCTGACTTCAGGTCAAGGGATGGTATTTATGCTCGCCTTGCTGTAGACTTCCCAGATCTTCCAGATCCTCAAGCGAT | sirtuin 1 |
| SKI | NM_003036.2 | v-ski avian sarcoma viral oncogene homolog | CCTCTGGTGCTTGGTTGAACAAGGGAATCACAAGAAAACGAAAATGCAAAAACTGAACTTCGGGGGTCGTTCTGTGCCTTCCAGCATCTTGTACAGCAAA | SKI proto-oncogene |
| SKP1 | NM_170679.2 | SKP1A;S-phase kinase-associated protein 1A (p19A) | CCCAGGTACGCAAAGAGAACCAGTGGTGTGAAGAGAAGTGAAATGTTGTGCCTGACACTGTAACACTGTAAGGATTGTTCCAAATACTAGTTGCACTGCT | S-phase kinase associated protein 1 |
| SKP2 | NM_005983.2 | S-phase kinase-associated protein 2 (p45),S-phase kinase-associated protein 2, E3 ubiquitin protein ligase | AAAATCTGCACCCGGATGTGACTGGTCGGTTGCTGTCTCAAGGGGTGATTGCCTTCCGCTGCCCACGATCATTTATGGACCAACCATTGGCTGAACATTT | S-phase kinase associated protein 2 |
| SLC25A10 | NM_012140.3 | DIC;solute carrier family 25 (mitochondrial carrier; dicarboxylate transporter), member 10 | AGGTGTTGCTGGGCTCCGTCAGCGGTTTAGCTGGAGGCTTCGTGGGGACGCCCGCAGACTTGGTCAACGTCAGGATGCAGAACGACGTGAAGCTGCCCCA | solute carrier family 25 member 10 |
| SLC25A13 | NM_001160210.1 | CTLN2;solute carrier family 25, member 13 (citrin),solute carrier family 25 (aspartate/glutamate carrier), member 13 | TGGAGCCACTGCTGTGTATCCTATCGATCTTGTAAAAACTCGAATGCAGAACCAACGATCAACTGGCTCTTTTGTGGGAGAACTCATGTATAAAAACAGC | solute carrier family 25 member 13 |
| SLC2A2 | NM_000340.1 | GLUT2;solute carrier family 2 (facilitated glucose transporter), member 2 | CAATTATGGAAATATAGTTCTGATGGGTCCCAAAAGCTTAGCAGGGTGCTAACGTATCTCTAGGCTGTTTTCTCCACCAACTGGAGCACTGATCAATCCT | solute carrier family 2 member 2 |
| SLC37A4 | NM_001164277.1 | G6PT1,G6PT2,G6PT3;glucose-6-phosphatase, transport (glucose-6-phosphate) protein 1,solute carrier family 37 (glucose-6-phosphate transporter), member 4 | CTGGCGCAGCACGCTGGCCCTATCTGGGGCACTGTGTGTGGTTGTCTCCTTCCTCTGTCTCCTGCTCATCCACAATGAACCTGCTGATGTTGGACTCCGC | solute carrier family 37 member 4 |
| SMAD2 | NM_001003652.3 | MADH2;MAD, mothers against decapentaplegic homolog 2 (Drosophila),SMAD, mothers against DPP homolog 2 (Drosophila) | GTATGTGTAAACCCTTACCACTATCAGAGAGTTGAGACACCAGTTTTGCCTCCAGTATTAGTGCCCCGACACACCGAGATCCTAACAGAACTTCCGCCTC | SMAD family member 2 |
| SMAD3 | NM_005902.3 | MADH3;MAD, mothers against decapentaplegic homolog 3 (Drosophila),SMAD, mothers against DPP homolog 3 (Drosophila) | TTAAAGGACAGTTGAAAAGGGCAAGAGGAAACCAGGGCAGTTCTAGAGGAGTGCTGGTGACTGGATAGCAGTTTTAAGTGGCGTTCACCTAGTCAACACG | SMAD family member 3 |
| SMAD4 | NM_005359.3 | MADH4;MAD, mothers against decapentaplegic homolog 4 (Drosophila),SMAD, mothers against DPP homolog 4 (Drosophila) | AGGTTGCACATAGGCAAAGGTGTGCAGTTGGAATGTAAAGGTGAAGGTGATGTTTGGGTCAGGTGCCTTAGTGACCACGCGGTCTTTGTACAGAGTTACT | SMAD family member 4 |
| SMAD6 | NR_027654.1 | MADH7,MADH6;MAD, mothers against decapentaplegic homolog 6 (Drosophila),SMAD, mothers against DPP homolog 6 (Drosophila) | GAATCTCCGCCACCTCCCTACTCTCGGCTGTCTCCTCGCGACGAGTACAAGCCACTGGATCTGTCCGATTCCACATTGTCTTACACTGAAACGGAGGCTA | SMAD family member 6 |
| SMARCC2 | NM_001130420.1 | SWI/SNF related, matrix associated, actin dependent regulator of chromatin, subfamily c, member 2 | AGTCCCTGTCTAGCCTGGTTGTACAGTTGCTACAATTTCAGGAAGAAGTTTTTGGCAAACATGTCAGCAATGCACCGCTCACTAAACTGCCGATCAAATG | SWI/SNF related, matrix associated, actin dependent regulator of chromatin subfamily c member 2 |
| SMURF1 | NM_181349.1 |  | AAGAGAATCTGCCACCAGATCGAATTTCGACCCCTGAGCTTGTTCGGACGTATGGTCCAAATTCAGATTAAGGTGGTCACCCAACCCGAGATGTCAGGAA | SMAD specific E3 ubiquitin protein ligase 1 |
| SNAI2 | NM_003068.3 | SLUG;slug homolog, zinc finger protein (chicken),snail homolog 2 (Drosophila),snail family zinc finger 2 | GCGTTTTCCAGACCCTGGTTGCTTCAAGGACACATTAGAACTCACACGGGGGAGAAGCCTTTTTCTTGCCCTCACTGCAACAGAGCATTTGCAGACAGGT | snail family transcriptional repressor 2 |
| SOD1 | NM_000454.4 | ALS,ALS1;amyotrophic lateral sclerosis 1 (adult),superoxide dismutase 1, soluble | GCCTATAAAGTAGTCGCGGAGACGGGGTGCTGGTTTGCGTCGTAGTCTCCTGCAGCGTCTGGGGTTTCCGTTGCAGTCCTCGGAACCAGGACCTCGGCGT | superoxide dismutase 1 |
| SORBS1 | NM_001034956.1 | SH3D5;SH3-domain protein 5 (ponsin) | CCTCTCTTAGATCCTGAGTGAGACAAATACAGAAATGACCCATTCCCTGCCCACCAGAAACTCAGAGGTGATTGGGGAGACTGACACAGGAAAATGAACT | sorbin and SH3 domain containing 1 |
| SOS1 | NM_005633.2 | GINGF;gingival fibromatosis, hereditary, 1,son of sevenless homolog 1 (Drosophila) | TACCGGAGTACACTGGAAAGGATGCTTGATGTAACAATGCTACAGGAAGAGAAAGAGGAGCAGATGAGGCTGCCTAGTGCTGATGTTTATAGATTTGCAG | SOS Ras/Rac guanine nucleotide exchange factor 1 |
| SP1 | NM_003109.1 |  | AGCCCTGGTGCTACTTGCTTGAAGTTTTCAGTGTAAGTACCCTGATGCCTTTTGGACCTTGGGATCAGATCAAGAGTTTTGGAGATCAGGTACCAAGGAA | Sp1 transcription factor |
| SP3 | NM_003111.3 |  | AGTGTCCAGTGTTCAATATCAAGTGATACCACAGATCCAGTCAGCAGATGGTCAGCAGGTTCAAATTGGTTTCACAGGCTCTTCAGATAATGGGGGTATA | Sp3 transcription factor |
| SPIB | NM_003121.3 | Spi-B transcription factor (Spi-1/PU.1 related) | CTTTGTCATGTACAGACTCCCTGGGATCCTCATGTTTTGGGTGACAGGACCTATGGACCACTATACTCGGGGAGGCAGGGTAGCAGTTCTTCCAGAATCC | Spi-B transcription factor |
| SPOP | NM_001007226.1 | speckle-type POZ protein | CCTCCACAGTGCAGATCAGTTGAAAACTCAGGCAGTGGATTTCATCAACTATCATGCTTCGGATGTCTTGGAGACCTCTGGGTGGAAGTCAATGGTGGTG | speckle type BTB/POZ protein |
| SPOPL | NM_001001664.1 | speckle-type POZ protein-like | GATTCAGTCTCACCCTCATTTAGTAGCAGAAGCCTTTCGAGCACTAGCATCTGCACAGTGTCCACAGTTTGGCATTCCACGCAAACGGCTAAAACAGTCC | speckle type BTB/POZ protein like |
| SPP1 | NM_000582.2 | BNSP,OPN;osteopontin,bone sialoprotein I | CGCCTTCTGATTGGGACAGCCGTGGGAAGGACAGTTATGAAACGAGTCAGCTGGATGACCAGAGTGCTGAAACCCACAGCCACAAGCAGTCCAGATTATA | secreted phosphoprotein 1 |
| SRC | NM_005417.3 | SRC1;v-src avian sarcoma (Schmidt-Ruppin A-2) viral oncogene homolog | GGCATGAGAAGCTGGTGCAGTTGTATGCTGTGGTTTCAGAGGAGCCCATTTACATCGTCACGGAGTACATGAGCAAGGGGAGTTTGCTGGACTTTCTCAA | SRC proto-oncogene, non-receptor tyrosine kinase |
| SREBF1 | NM_001005291.1 |  | TTCGCTTTCTGCAACACAGCAACCAGAAACTCAAGCAGGAGAACCTAAGTCTGCGCACTGCTGTCCACAAAAGCAAATCTCTGAAGGATCTGGTGTCGGC | sterol regulatory element binding transcription factor 1 |
| SSR2 | NM_003145.3 | signal sequence receptor, beta (translocon-associated protein beta) | ACCTGACCTTGCAGTACAACATCTACAATGTTGGCTCAAGTGCTGCATTAGACGTGGAACTATCTGATGATTCCTTCCCTCCAGAAGACTTTGGCATTGT | signal sequence receptor subunit 2 |
| SSR4 | NM_006280.1 | signal sequence receptor, delta | CCTATGAGGTTAGATTCTTCGACGAGGAGTCCTACAGCCTCCTCAGGAAGGCTCAGAGGAATAACGAGGACATTTCCATCATCCCGCCTCTGTTTACAGT | signal sequence receptor subunit 4 |
| STAT1 | NM_139266.1 | signal transducer and activator of transcription 1, 91kD,signal transducer and activator of transcription 1, 91kDa | ACAGTGGTTAGAAAAGCAAGACTGGGAGCACGCTGCCAATGATGTTTCATTTGCCACCATCCGTTTTCATGACCTCCTGTCACAGCTGGATGATCAATAT | signal transducer and activator of transcription 1 |
| STAT3 | NM_003150.3 | signal transducer and activator of transcription 3 (acute-phase response factor) | AAAGAAGGAGGCGTCACTTTCACTTGGGTGGAGAAGGACATCAGCGGTAAGACCCAGATCCAGTCCGTGGAACCATACACAAAGCAGCAGCTGAACAACA | signal transducer and activator of transcription 3 |
| STAT5A | NM_003152.2 | STAT5 | GAGACAGAGAGAGAGAAAGAGAGAGTGTGTGGGTCTATGTAAATGCATCTGTCCTCATGTGTTGATGTAACCGATTCATCTCTCAGAAGGGAGGCTGGGG | signal transducer and activator of transcription 5A |
| STAT5B | NM_012448.3 |  | AAGGAGAAGCCCTTCATCAGATGCAAGCGTTATATGGCCAGCATTTTCCCATTGAGGTGCGGCATTATTTATCCCAGTGGATTGAAAGCCAAGCATGGGA | signal transducer and activator of transcription 5B |
| STK4 | NR_147975.1 |  | CAGCTGAGGAACCCGCCGCGCCGGCAGCTGAAAAAGTTGGATGAAGATAGTTTAACCAAACAACCAGAAGAAGTATTTGATGTCTTAGAGAAACTTGGAG | serine/threonine kinase 4 |
| SUFU | NM_001178133.1 | suppressor of fused homolog (Drosophila) | ATCAGCTTCGGCCTGAGTGATCTCTATGGTGACAACAGAGTCCATGAGTTTACAGGAACAGATGGACCTAGTGGTTTTGGCTTTGAGTTGACCTTTCGTC | SUFU negative regulator of hedgehog signaling |
| SUGT1 | NM_001130912.1 | SGT1, suppressor of G2 allele of SKP1 (S. cerevisiae) | ATGGTTCTGATGAAGTGAAACGTGCCATGAACAAATCCTTTATGGAGTCGGGTGGTACAGTTTTGAGTACCAACTGGTCTGATGTAGGTAAAAGGAAAGT | SGT1 homolog, MIS12 kinetochore complex assembly cochaperone |
| SYK | NM_003177.5 | spleen tyrosine kinase | TCCTACGCCCTGTGCCTGCTGCACGAAGGGAAGGTGCTGCACTATCGCATCGACAAAGACAAGACAGGGAAGCTCTCCATCCCCGAGGGAAAGAAGTTCG | spleen associated tyrosine kinase |
| TBC1D4 | NM_014832.2 | TBC1 domain family, member 4 | GCAGTTTCTGGCTTTACAGTACCGACTCAGACACAGATTGCCTAATAAACAACAGCCTCCTGACATATCCTATAAGGAACTTTTGAAGCAGCTCACTGCT | TBC1 domain family member 4 |
| TBL1XR1 | NM_024665.4 | transducin (beta)-like 1X-linked receptor 1 | GGGCAAGATGTTCCAAGCAACAAGGATGTCACATCTCTAGATTGGAATAGTGAAGGTACACTTCTAGCAACTGGTTCCTATGATGGGTTTGCCAGAATAT | transducin beta like 1 X-linked receptor 1 |
| TBX21 | NM_013351.1 |  | ACACAGGAGCGCACTGGATGCGCCAGGAAGTTTCATTTGGGAAACTAAAGCTCACAAACAACAAGGGGGCGTCCAACAATGTGACCCAGATGATTGTGCT | T-box 21 |
| TCF7L1 | NM_031283.1 | TCF3;transcription factor 7-like 1 (T-cell specific, HMG-box) | TGCAGCTCTGCCATTGTGACATTTCCTGTTACCCAGCCCAAGTTTTCATCGTCTGCTCAATACCGTGGGTTCTTCTTCGTCCTCTGTCCTCTGCCCAGTG | transcription factor 7 like 1 |
| TCF7L2 | NM_001146274.1 | TCF4;transcription factor 7-like 2 (T-cell specific, HMG-box) | ATATGGTCCCACCACATCATACGCTACACACGACGGGCATTCCGCATCCGGCCATAGTCACACCAACAGTCAAACAGGAATCGTCCCAGAGTGATGTCGG | transcription factor 7 like 2 |
| TCL1A | NR_049726.1 | T-cell leukemia/lymphoma 1A | CCCACCCAGATAGGCCCAAGCCTGCTGCCTATCATGTGGCAGCTCTACCCTGATGGACGATACCGATCCTCAGACTCCAGTTTCTGGCGCTTAGTGTACC | T cell leukemia/lymphoma 1A |
| TEK | NM_000459.3 | VMCM;venous malformations, multiple cutaneous and mucosal,TEK tyrosine kinase, endothelial | CCCGTTAATCACTATGAGGCTTGGCAACATATTCAAGTGACAAATGAGATTGTTACACTCAACTATTTGGAACCTCGGACAGAATATGAACTCTGTGTGC | TEK receptor tyrosine kinase |
| TGFB1 | NM_000660.3 | TGFB,DPD1;transforming growth factor, beta 1 | TATATGTTCTTCAACACATCAGAGCTCCGAGAAGCGGTACCTGAACCCGTGTTGCTCTCCCGGGCAGAGCTGCGTCTGCTGAGGCTCAAGTTAAAAGTGG | transforming growth factor beta 1 |
| TGFB1I1 | NM_001042454.1 |  | AATCATGTCTCAGTTCCCATCTAGCAAGGTGGCTTCAGGAGAGCAGAAGGAGGACCAGTCTGAAGATAAGAAAAGACCCAGCCTCCCTTCCAGCCCGTCT | transforming growth factor beta 1 induced transcript 1 |
| TGFBR1 | NM_004612.2 | MSSE,ESS1;multiple self-healing squamous epithelioma,transforming growth factor beta receptor I | GAATCCTTCAAACGTGCTGACATCTATGCAATGGGCTTAGTATTCTGGGAAATTGCTCGACGATGTTCCATTGGTGGAATTCATGAAGATTACCAACTGC | transforming growth factor beta receptor 1 |
| TGFBR2 | NM_001024847.1 | MFS2;transforming growth factor, beta receptor II (70/80kDa),transforming growth factor beta receptor II | ATTTGGAGAATGTTGAGTCCTTCAAGCAGACCGATGTCTACTCCATGGCTCTGGTGCTCTGGGAAATGACATCTCGCTGTAATGCAGTGGGAGAAGTAAA | transforming growth factor beta receptor 2 |
| THBS1 | NM_003246.2 |  | ACCCTCGTCACATAGGCTGGAAAGATTTCACCGCCTACAGATGGCGTCTCAGCCACAGGCCAAAGACGGGTTTCATTAGAGTGGTGATGTATGAAGGGAA | thrombospondin 1 |
| THBS2 | NM_003247.2 |  | AAACATCCTTGCAAATGGGTGTGACGCGGTTCCAGATGTGGATTTGGCAAAACCTCATTTAAGTAAAAGGTTAGCAGAGCAAAGTGCGGTGCTTTAGCTG | thrombospondin 2 |
| THBS3 | NM_001252607.1 |  | CTGCGGGATGATATACGAGACCAGGTGAAGGAAATGTCCCTGATCCGAAACACCATTATGGAGTGTCAGGTGTGCGGCTTCCATGAGCAGCGTTCCCACT | thrombospondin 3 |
| TIMP1 | NM_003254.2 | TIMP,CLGI;tissue inhibitor of metalloproteinase 1 (erythroid potentiating activity, collagenase inhibitor) | CGTGGGGACACCAGAAGTCAACCAGACCACCTTATACCAGCGTTATGAGATCAAGATGACCAAGATGTATAAAGGGTTCCAAGCCTTAGGGGATGCCGCT | TIMP metallopeptidase inhibitor 1 |
| TIMP2 | NM_003255.4 | tissue inhibitor of metalloproteinase 2 | GAAGGAGCCCCATCAATCCTATTAATCCTCAGAATTCCAGTGGGAGCCTCCCTCTGAGCCTTGTAGAAATGGGAGCGAGAAACCCCAGCTGAGCTGCGTT | TIMP metallopeptidase inhibitor 2 |
| TJP1 | NM_003257.3 |  | CACATTTTTCTTAGGGAAGGATACAAAAGCATGTGAGACTGGTTCCATGGCCTCTTCAGATCTCTAACTTCACCATATTACCACAGACATACTAACCAGC | tight junction protein 1 |
| TJP2 | NM_004817.2 | DFNA51;deafness, autosomal dominant 51 | AGCCAATGATAGCTGGTTTGGCAGCTTAAAGGACACTATTCAGCATCAGCAAGGAGAAGCGGTTTGGGTCTCTGAAGGAAAGATGGAAGGGATGGATGAT | tight junction protein 2 |
| TLN1 | NM_006289.3 | TLN | TTTTGGAGATTACCAAGATGGCTATTACTCAGTACAGACAACTGAAGGGGAGCAGATTGCACAGCTCATTGCCGGCTACATCGATATCATCCTGAAGAAG | talin 1 |
| TLR1 | NM_003263.3 | toll-like receptor 1 | TCAACCAGGAATTGGAATACTTGGATTTGTCCCACAACAAGTTGGTGAAGATTTCTTGCCACCCTACTGTGAACCTCAAGCACTTGGACCTGTCATTTAA | toll like receptor 1 |
| TLR2 | NM_003264.3 | toll-like receptor 2 | CAATGATGCTGCCATTCTCATTCTTCTGGAGCCCATTGAGAAAAAAGCCATTCCCCAGCGCTTCTGCAAGCTGCGGAAGATAATGAACACCAAGACCTAC | toll like receptor 2 |
| TLR4 | NM_138554.2 | toll-like receptor 4 | ACTCAGAAAAGCCCTGCTGGATGGTAAATCATGGAATCCAGAAGGAACAGTGGGTACAGGATGCAATTGGCAGGAAGCAACATCTATCTGAAGAGGAAAA | toll like receptor 4 |
| TLR6 | NM_006068.2 | toll-like receptor 6 | TGTCTGGAGGTGCCTCCATTATCCTCATGCCTTCAGGAAAGACTTAACAAAAACAATGTTTCATCTGGGGAACTGAGCTAGGCGGTGAGGTTAGCCTGCC | toll like receptor 6 |
| TLR8 | NM_016610.2 | toll-like receptor 8 | TTTAACTGATAGCCTATCTGACTTTACATCTTCCCTTCGGACACTGCTGCTGAGTCATAACAGGATTTCCCACCTACCCTCTGGCTTTCTTTCTGAAGTC | toll like receptor 8 |
| TM6SF2 | NM_001001524.2 |  | TGGCATTGATGAGCGCCCTAATCCTGGGTCTGCTTTTCGTGGCGGTCTACAGCTTGTCCCATGGCGAGGTCTCCTATGACCCACTCTATGCTGTCTTCGC | transmembrane 6 superfamily member 2 |
| TNF | NM_000594.2 | TNFA;tumor necrosis factor (TNF superfamily, member 2) | AGCAACAAGACCACCACTTCGAAACCTGGGATTCAGGAATGTGTGGCCTGCACAGTGAAGTGCTGGCAACCACTAAGAATTCAAACTGGGGCCTCCAGAA | tumor necrosis factor |
| TNFRSF17 | NM_001192.2 | BCMA;tumor necrosis factor receptor superfamily, member 17 | TCTGACCATTGCTTTCCACTCCCAGCTATGGAGGAAGGCGCAACCATTCTTGTCACCACGAAAACGAATGACTATTGCAAGAGCCTGCCAGCTGCTTTGA | TNF receptor superfamily member 17 |
| TNFSF10 | NM_003810.2 | tumor necrosis factor (ligand) superfamily, member 10 | GGGGGGACCCAGCCTGGGACAGACCTGCGTGCTGATCGTGATCTTCACAGTGCTCCTGCAGTCTCTCTGTGTGGCTGTAACTTACGTGTACTTTACCAAC | TNF superfamily member 10 |
| TNFSF14 | NM_003807.3 | tumor necrosis factor (ligand) superfamily, member 14 | GGCGTGTCAGCCCTGCTCCAGACACCTTGGGCATGGAGGAGAGTGTCGTACGGCCCTCAGTGTTTGTGGTGGATGGACAGACCGACATCCCATTCACGAG | TNF superfamily member 14 |
| TNN | NM_022093.1 |  | GCGGTATGAGGTGAGAGTGGATTTACAGACTGCCAATGAATCTGCCTATGCTATATATGATTTCTTCCAAGTGGCCTCCAGCAAGGAGCGGTATAAGCTG | tenascin N |
| TPR | NM_003292.2 | translocated promoter region (to activated MET oncogene) | TCTAGTCACTCTGATCTTGGCCAGCTTGCTTCTCAAGGAGGTTTAGGAATGTATGAAACACCCCTGTTCCTAGCTCATGAAGAAGAGTCAGGTGGCCGAA | translocated promoter region, nuclear basket protein |
| TPSAB1/B2 | NM_003294.3 |  | GCAGGTGAAGGTCCCCATAATGGAAAACCACATTTGTGACGCAAAATACCACCTTGGCGCCTACACGGGAGACGACGTCCGCATCGTCCGTGACGACATG | tryptase alpha/beta 1 |
| TRADD | NM_003789.2 | TNFRSF1A-associated via death domain | TGTTCCAGGGTCAGCCTGTAGTGAATCGGCCGCTGAGCCTGAAGGACCAACAGACGTTCGCGCGCTCTGTGGGTCTCAAATGGCGCAAGGTGGGGCGCTC | TNFRSF1A associated via death domain |
| TRAF2 | NM_021138.3 |  | GTGGCCCTTCAACCAGAAGGTGACCTTAATGCTGCTCGACCAGAATAACCGGGAGCACGTGATTGACGCCTTCAGGCCCGACGTGACTTCATCCTCTTTT | TNF receptor associated factor 2 |
| TRAF6 | NM_145803.2 | TNF receptor-associated factor 6, E3 ubiquitin protein ligase | TTGCAAAACGTGAGATTCTTTCTCTGATGGTGAAATGTCCAAATGAAGGTTGTTTGCACAAGATGGAACTGAGACATCTTGAGGATCATCAAGCACATTG | TNF receptor associated factor 6 |
| TRAT1 | NM_016388.2 | TCRIM;TCRIM | ACAGAGGACACAGAAGGACTTGGCAGCAGGGTGATGACCTGATCATTTGTTGATGGGATGGTGGCTTACCTCTTATTCACAGCTTACACTTATGCATGCC | T cell receptor associated transmembrane adaptor 1 |
| TRIB3 | NM_021158.3 | C20orf97;chromosome 20 open reading frame 97,tribbles homolog 3 (Drosophila) | ATATTCCCTGCTCACAGAGATGACAAACTGGCATCCTTGAGCTGACAACACTTTTCCATGACCATAGGTCACTGTCTACACTGGGTACACTTTGTACCAG | tribbles pseudokinase 3 |
| TRRAP | NM_003496.2 | transformation/transcription domain-associated protein | GAAATGGTTGGTATGATAACAACGATTGCTGTGAAAGTCAACCCGGAGCGTGAGGACAGTGAGACTCGAACACATTCCATCATTCCGAGGGGATCACTTT | transformation/transcription domain associated protein |
| TTN | NM_133432.1 | CMD1G;cardiomyopathy, dilated 1G (autosomal dominant) | TTTATCTCTGAGACCAGACAGAGCGATGCAGGAGAATACACCTTTGTGGCAGGAAGGAACAGGAGTTCTGTCACTCTCTATGTCAATGCTCCTGAACCGC | titin |
| TXN | NM_003329.2 |  | CAGCCAAGATGGTGAAGCAGATCGAGAGCAAGACTGCTTTTCAGGAAGCCTTGGACGCTGCAGGTGATAAACTTGTAGTAGTTGACTTCTCAGCCACGTG | thioredoxin |
| TXN2 | NM_012473.3 |  | CTGGTGGCCTGACTGTAACACCCAACCCAGCCCGGACAATATACACCACGAGGATCTCCTTGACAACCTTTAATATCCAGGATGGACCTGACTTTCAAGA | thioredoxin 2 |
| TXNDC5 | NM_030810.2 |  | AGCAGATTGAAATGCAAAAACCCACACCTCTGGAAGATACCTTCACGGCCGCTGCTGGAGCTTCTGTTGCTGTGAATACTTCTCTCAGTGTGAGAGGTTA | thioredoxin domain containing 5 |
| UBA52 | NM_003333.3 |  | CCCTGCACCTGGTGTTGCGCCTGCGAGGTGGCATTATTGAGCCTTCTCTCCGCCAGCTTGCCCAGAAATACAACTGCGACAAGATGATCTGCCGCAAGTG | ubiquitin A-52 residue ribosomal protein fusion product 1 |
| UBE2D2 | NM_181838.1 | ubiquitin-conjugating enzyme E2D 2 (homologous to yeast UBC4/5),ubiquitin-conjugating enzyme E2D 2 (UBC4/5 homolog, yeast),ubiquitin-conjugating enzyme E2D 2 | CAAAAGTACTCTTGTCCATCTGTTCTCTGTTGTGTGATCCCAATCCAGATGATCCTTTAGTGCCTGAGATTGCTCGGATCTACAAAACAGATAGAGAAAA | ubiquitin conjugating enzyme E2 D2 |
| UBE2N | NM_003348.3 | ubiquitin-conjugating enzyme E2N (homologous to yeast UBC13),ubiquitin-conjugating enzyme E2N (UBC13 homolog, yeast),ubiquitin-conjugating enzyme E2N | AAGAAACACTGATCTGATCATTTGGGATTTGCTGAGGCATTTGTGAGTCTTCCTTATAAACCTGATGAGCAGATCTCAACTATCTAGCTTGTGTGTCATC | ubiquitin conjugating enzyme E2 N |
| UBE4B | NM_006048.4 | ubiquitination factor E4B (homologous to yeast UFD2),ubiquitination factor E4B (UFD2 homolog, yeast) | TCTAGTTTGGGAGCCTCTGGTGGAGCAAGTAATTGGGATTCCTACAGTGACCATTTCACCATTGAAACCTGCAAAGAGACAGATATGCTGAACTACCTCA | ubiquitination factor E4B |
| UQCRB | NM_006294.3 | UQBP | GAGAGGGATGGTTAGAAAGTGAGTAAGACAGGTTGCTCTTTGGAAAACAAGGAAAATCCCTTTATTAGAACAAGGCATGAAATTCTGCCACTAGGTGGCG | ubiquinol-cytochrome c reductase binding protein |
| UQCRFS1 | NM_006003.2 |  | GATCAGATTGGGTCCTGCTCCTCTCAACCTTGAAGTCCCCACGTATGAGTTCACCAGTGACGATATGGTGATTGTTGGTTAAGAGACTTGGACTCAAGTC | ubiquinol-cytochrome c reductase, Rieske iron-sulfur polypeptide 1 |
| UQCRH | NM_006004.2 |  | TCGATCACATACAGAAGAGGATTGCACGGAGGAGCTCTTTGACTTCTTGCATGCGAGGGACCATTGCGTGGCCCACAAACTCTTTAACAACTTGAAATAA | ubiquinol-cytochrome c reductase hinge protein |
| VAMP8 | NM_003761.4 |  | GAAGCCGACTAGGCGAATTCACTTACTGACCGGCCTGGGCTGCTCTGAGACATGGAGGAAGCCAGTGAAGGTGGAGGAAATGATCGTGTGCGGAACCTGC | vesicle associated membrane protein 8 |
| VAV1 | NM_005428.2 | VAV;vav 1 oncogene,vav 1 guanine nucleotide exchange factor | CCCTGCAACGGTTCCTGAAACCTCAAGACATTGAGATCATCTTTATCAACATTGAGGACCTGCTTCGTGTTCATACTCACTTCCTAAAGGAGATGAAGGA | vav guanine nucleotide exchange factor 1 |
| VCAM1 | NM_001078.3 |  | CAGACTTCCCTGAATGTATTGAACTTGGAAAGAAATGCCCATCTATGTCCCTTGCTGTGAGCAAGAAGTCAAAGTAAAACTTGCTGCCTGAAGAACAGTA | vascular cell adhesion molecule 1 |
| VEGFA | NM_001025366.1 | VEGF;vascular endothelial growth factor | GAGTCCAACATCACCATGCAGATTATGCGGATCAAACCTCACCAAGGCCAGCACATAGGAGAGATGAGCTTCCTACAGCACAACAAATGTGAATGCAGAC | vascular endothelial growth factor A |
| VEGFB | NM_003377.3 | VRF | TGCCGGAAGCTGCGAAGGTGACACATGGCTTTTCAGACTCAGCAGGGTGACTTGCCTCAGAGGCTATATCCCAGTGGGGGAACAAAGAGGAGCCTGGTAA | vascular endothelial growth factor B |
| VIM | NM_003380.2 |  | GAGGAGATGCTTCAGAGAGAGGAAGCCGAAAACACCCTGCAATCTTTCAGACAGGATGTTGACAATGCGTCTCTGGCACGTCTTGACCTTGAACGCAAAG | vimentin |
| WAS | NM_000377.2 | IMD2,THC;thrombocytopenia 1 (X-linked),Wiskott-Aldrich syndrome (eczema-thrombocytopenia),Wiskott-Aldrich syndrome | GGCCGAGGAGCACCAGCGGTTCAGCAGAACATACCCTCCACCCTCCTCCAGGACCACGAGAACCAGCGACTCTTTGAGATGCTTGGACGAAAATGCTTGA | WASP actin nucleation promoting factor |
| WWC1 | NM_001161662.1 | WW, C2 and coiled-coil domain containing 1 | GAGAGATGGTTCACCTCCAGCACGAGCTGCAGTTCAAAGAGCGTGGCTTTCAGACCCTGAAGAAAATCGATAAGAAAATGTCTGATGCTCAGGGCAGCTA | WW and C2 domain containing 1 |
| XAF1 | NM_199139.2 |  | GGCTTACTGCCTGCGGTTCCTGGTCCTGTGTCCGGAGTGTGAGGAGCCTGTCCCCAAGGAAACCATGGAGGAGCACTGCAAGCTTGAGCACCAGCAGGCC | XIAP associated factor 1 |
| XBP1 | NM_005080.2 | XBP2 | GGAGTTAAGACAGCGCTTGGGGATGGATGCCCTGGTTGCTGAAGAGGAGGCGGAAGCCAAGGGGAATGAAGTGAGGCCAGTGGCCGGGTCTGCTGAGTCC | X-box binding protein 1 |
| XCL1/2 | NM_003175.3 | SCYC2;small inducible cytokine subfamily C, member 2,chemokine (C motif) ligand 2 | GTAGTCTCTGGCACCCTGTCCGTCTCCAGCCAGCCAGCTCATTTCACTTTACACCCTCATGGACTGAGATTATACTCACCTTTTATGAAAGCACTGCATG | X-C motif chemokine ligand 2 |
| XIAP | NM_001167.3 | API3,BIRC4;baculoviral IAP repeat-containing 4,X-linked inhibitor of apoptosis, E3 ubiquitin protein ligase | GAGCAACTATAAATCACTTGAGGTTCTGGTTGCAGATCTAGTGAATGCTCAGAAAGACAGTATGCAAGATGAGTCAAGTCAGACTTCATTACAGAAAGAG | X-linked inhibitor of apoptosis |
| YWHAB | NM_003404.4 | YWHAA;tyrosine 3-monooxygenase/tryptophan 5-monooxygenase activation protein, alpha polypeptide,tyrosine 3-monooxygenase/tryptophan 5-monooxygenase activation protein, beta polypeptide | GAACAGGGGCATGAACTCTCCAACGAAGAGAGAAATCTGCTCTCTGTTGCCTACAAGAATGTGGTAGGCGCCCGCCGCTCTTCCTGGCGTGTCATCTCCA | tyrosine 3-monooxygenase/tryptophan 5-monooxygenase activation protein beta |
| YWHAE | NM_006761.4 | tyrosine 3-monooxygenase/tryptophan 5-monooxygenase activation protein, epsilon polypeptide | GATACGCTGAGTGAAGAAAGCTATAAGGACTCTACACTTATCATGCAGTTGTTACGTGATAATCTGACACTATGGACTTCAGACATGCAGGGTGACGGTG | tyrosine 3-monooxygenase/tryptophan 5-monooxygenase activation protein epsilon |
| YWHAG | NM_012479.3 | tyrosine 3-monooxygenase/tryptophan 5-monooxygenase activation protein, gamma polypeptide | CTCATCTGTCTCGGCTCTGCATGTTTTCCAGGGTGTAGCCTACAGACATGGAACAGTGTAAATCCCAGACTGACAGACTTAGAACCTGAGGTCTCATTCA | tyrosine 3-monooxygenase/tryptophan 5-monooxygenase activation protein gamma |
| YWHAQ | NM_006826.2 | tyrosine 3-monooxygenase/tryptophan 5-monooxygenase activation protein, theta polypeptide,protein, theta | GAAAAGCACTTGCCATCTCTGTCTAGGGGTCACAAATTGAAATGGCTCCTGTATCACATACGGAGGTCTTGTGTATCTGTGGCAACAGGGAGTTTCCTTA | tyrosine 3-monooxygenase/tryptophan 5-monooxygenase activation protein theta |
| ZFYVE16 | NM_001105251.2 | zinc finger, FYVE domain containing 16 | CAAAGGACCTGAATAAGCCAGATGTTCCAGATACAATAGAAAGTGAACCCAGCACAGCAGATACCGTTGTTCCAATCACTTGTGCTATAGATTCTACAGC | zinc finger FYVE-type containing 16 |

Supplementary Table: Genes Analysed in the Nanostring Human Fibrosis V2.0 Panel
